# From Inception to Innovation: 20 Years of Nanoarchitectonics

**DOI:** 10.1002/asia.202500836

**Published:** 2025-08-31

**Authors:** Katsuhiko Ariga, Jingwen Song, Kohsaku Kawakami

**Affiliations:** ^1^ Research Center for Materials Nanoarchitectonics National Institute for Materials Science (NIMS) 1‐1 Namiki Tsukuba 305‐0044 Japan; ^2^ Graduate School of Frontier Sciences The University of Tokyo 5‐1‐5 Kashiwa‐no‐ha Kashiwa 277–8561 Japan; ^3^ Research Center for Macromolecules and Biomaterials National Institute for Materials Science (NIMS) 1‐1 Namiki Tsukuba 305‐0044 Japan; ^4^ Graduate School of Sciences and Technology University of Tsukuba 1‐1‐1 Tennodai Tsukuba 305–8577 Japan

**Keywords:** Interface, Layer‐by‐layer, Liquid interface, Manipulation, Nanoarchitectonics

## Abstract

The term “nanoarchitectonics” emerged as the 21st century approached, and it has been in use for around 20 years. We here look back accomplishments of nanoarchitectonics. However, this review will explore several typical topics and their trends instead of a comprehensive description in a chronicle‐like manner. Atomic switches and local probe chemistry handle basic‐level nanoarchitectonics involving atomic/molecular manipulations. Even within these typical approaches to nanoarchitectonics, the latest research focuses on artificial intelligence. Innovation is also coming to fundamental nanoarchitectonics. Nanoarchitectonics of two‐dimensional (2D) and layered materials are characterized by their potential for various practical applications. This lends nanoarchitectonics a sense of innovation with a view to practical applications. Nanoarchitectonics at liquid interfaces have broad applicability to a range of systems, from simple molecules to complex biological systems. The ability to control molecular structure through macroscopic movements that can be used for mass production paves the way for nanoarchitectonics to be applied in industry. The number of papers claiming to be in this field has increased significantly in recent years, as has the scope of its applications. Given these limitless advancements, one might say that nanoarchitectonics is a method for everything in materials science.

## Graphical abstract

Although nanotechnology was founded in the 20th century, the term nanoarchitectonics was first coined at the turn of the 21st century. Therefore, nanoarchitectonics has a history of about 20 years. In this review article, we will provide an overview of nanoarchitectonics through examining some research examples on manipulation‐based nanoarchitectonics of atoms and molecules, layered nanoarchitectonics, and nanoarchitectonics at liquid interfaces.

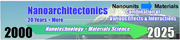

## 1 Introduction

**Figure 1:**
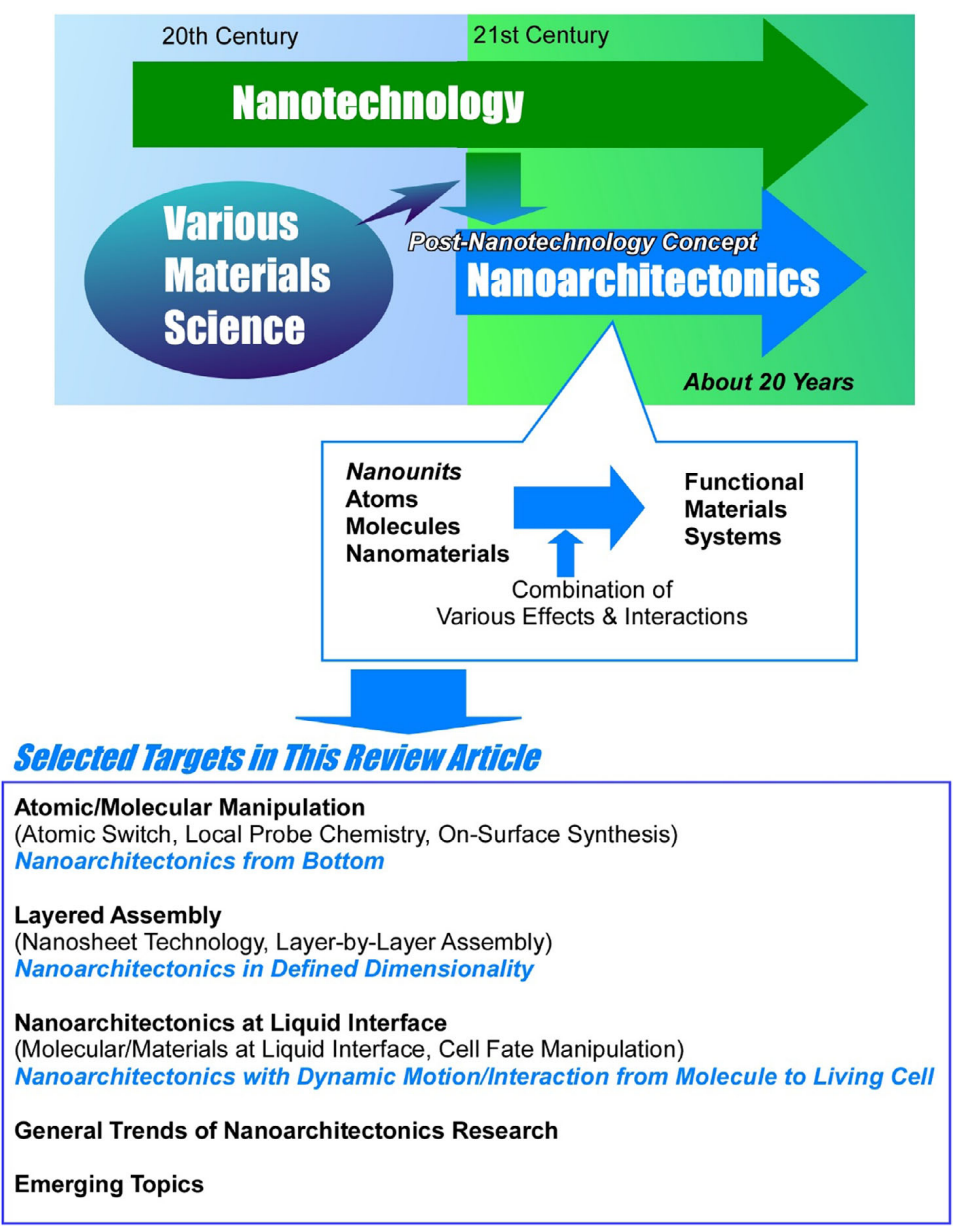
Outline and history of the nanoarchitectonics concept (top) and targets of this review article (bottom).

The concept of nanotechnology has had a significant impact on the development of chemistry, physics, and materials science from the 20th to the 21st century. Understanding and controlling precise nanoscale structures significantly impacts a wide range of properties and functionality of materials.^[^ ^1^ ^]^ Indeed, nanotechnology is a powerful driver of this trend. Rather than referring to specific techniques, nanotechnology has tasks as a concept that promotes science and technology generally at the nano level. Recently, nanoarchitectonics has emerged as a potential successor to nanotechnology.^[^ ^2^ ^]^ Nanoarchitectonics involves constructing functional materials from nanoscale units (Figure 1). While nanotechnology was founded by Richard Feynman in the mid‐20th century,^[^ ^3^ ^]^ the term nanoarchitectonics was first coined by Masakazu Aono at the turn of the 21st century.^[^ ^4^ ^]^ Therefore, nanoarchitectonics has a history of about 20 years. In this review article, we will provide an overview of nanoarchitectonics through examining some research examples.

### 1.1 Nanoarchitectonics as an Emerging Concept

By way of introduction, we will briefly explain the background to the emergence of the concept of nanoarchitectonics. Humanity's social development depends on an abundance of functional materials. To this end, it is essential to develop scientific disciplines that can freely create desired functional materials. In the 20th century, the following scientific fields for creating materials have been developed and with continuous contributions: organic chemistry,^[^ ^5^ ^]^ inorganic chemistry,^[^ ^6^ ^]^ coordination chemistry,^[^ ^7^ ^]^ polymer chemistry,^[^ ^8^ ^]^ supramolecular chemistry,^[^ ^9^ ^]^ biochemistry,^[^ ^10^ ^]^ and other types of material chemistry.^[^ ^11^ ^]^ These fields continue to advance today, resulting in the development of functional materials that address energy,^[^ ^12^ ^]^ environmental,^[^ ^13^ ^]^ and biomedical^[^ ^14^ ^]^ issues. They also facilitate the development of new technologies, such as devices^[^ ^15^ ^]^ and sensors.^[^ ^16^ ^]^

These developments in material science highlight the importance of controlling nanostructures. Phenomena such as quantum effects specific to nanoscale dimensions,^[^ ^17^ ^]^ enhanced efficiency based on nanostructures, and the emergence of novel functions. These effects are created through the arrangement of fine structures, which are not observed in conventional bulk materials. This is strongly supported by nanotechnology, the collective term for the science and technology of manipulating matter at the nanoscale. Nano‐level analysis and control techniques have made a significant contribution to the development of functional materials with controlled nanostructures, a process that continues to this day. These include techniques for observing^[^ ^18^ ^]^ and manipulating^[^ ^19^ ^]^ atoms and molecules, as well as analyzing unique phenomena in the nanoscale region.^[^ ^20^ ^]^ With these accumulating efforts, nanotechnology has made a significant contribution to the development of functional materials.

The challenge lies in intentionally creating functional structures and materials with controlled nanostructures. To address this, two complementary strategies have emerged: 1) top‐down approaches utilizing micro‐ and nanofabrication techniques, and 2) bottom‐up methods employing supramolecular chemistry principles.^[^ ^21^ ^]^ Particularly in bottom‐up approaches, self‐assembly and self‐organization have become pivotal concepts for constructing functional material architectures.^[^ ^22^ ^]^ In many cases, these processes are based on the principle of simple equilibrium and its energy‐free shift. However, self‐organization processes involving energies far from equilibrium are also important in overcoming the limitations of equilibrium processes.^[^ ^23^ ^]^ The principles of high entropy, non‐equilibrium, and irreversibility can be incorporated into the traditional self‐assembly framework. For instance, through directed and guided assembly,^[^ ^24^ ^]^ researchers can now craft molecules or assembly units with controlled arrangement, position, and spacing. Meanwhile, instructed assembly,^[^ ^25^ ^]^ which can induce dynamic control where chemical cues, enzymatic triggers, or ligand–receptor interactions act as molecular switches to initiate and guide the assembly process. The organized features persist even after energy dissipation has ceased. Introducing non‐equilibrium processes, such as the immobilization of assemblies on solid surfaces and the microfabrication of materials using self‐organization processes, allows spatiotemporal control. Assembly emphasizing spatial localization, so‐called localized assembly,^[^ ^26^ ^]^ enables target molecules to be collected at a specific location, resulting in functional structures of varying complexity with greater precision. Alongside conceptual approaches, several concrete material chemistry processes have been developed, including the creation of metal–organic frameworks (MOFs)^[^ ^27^ ^]^ through coordination chemistry, covalent organic frameworks (COFs)^[^ ^28^ ^]^ through polymer chemistry, and porous materials through template synthesis,^[^ ^29^ ^]^ thin‐film techniques such as self‐assembled monolayers (SAMs),^[^ ^30^ ^]^ the Langmuir–Blodgett (LB) method,^[^ ^31^ ^]^ and layer‐by‐layer (LbL) assembly^[^ ^32^ ^]^ through interfacial localized assembly in materials chemistry.

Accordingly, various methods have been employed to create functional materials using nanostructure control. The concept of nanoarchitectonics has emerged to integrate these methods. Just as nanotechnology comprehensively covers nano‐level phenomena and operations, nanoarchitectonics provides a comprehensive approach to constructing functional materials from nanounits.^[^ ^33^ ^]^ As a post‐nanotechnology concept, nanoarchitectonics effectively combines nanotechnology and various materials sciences.^[^ ^34^ ^]^ Therefore, nanoarchitectonics systematically coordinates key processes including: atoms/molecules manipulation, chemical/physical transformations, self‐assembly and self‐organization, external fields alignment, micro‐ and nano‐fabrication, and biochemical processes.^[^ ^35^ ^]^

### 1.2 Origin of Nanoarchitectonics

So, let's take a brief look at the historical background of how the term “nanoarchitectonics” entered the field of scientific research.^[^ ^36^ ^]^ Before this term was coined, the concept of architectonics (architecting) was recognized as important in nanoscale science during the transition from the 20th to the 21st century. This concept emerged simultaneously in many countries. In the United States, for example, Heath and his colleagues at the University of California, Los Angeles (UCLA) published a paper titled “Architectonic quantum dot solids” in 1999.^[^ ^37^ ^]^ Here, the term “architectonic” is defined as “having an organized and unified structure that suggests an architectural design” in Merriam‐Webster's WWW dictionary.^[^ ^38^ ^]^ The term was first proposed by Dr R. S. Williams of Hewlett–Packard Labs.

Masakazu Aono was the first person to use the term “nanoarchitectonics,” a combination of “nano” and “architectonics,” in the scientific field. In 2000, he organized a conference called “The 1st International Symposium on Nanoarchitectonics Using Suprainteractions” in Tsukuba, Japan. This was the first instance of the term “nanoarchitectonics” being used in the scientific communities. Later, in 2007, Aono founded the World Premier International Research Center for Materials Nanoarchitectonics (WPI‐MANA) at the National Institute for Materials Science (NIMS) in Tsukuba. In the same city, Toshimi Shimizu established the Interfacial Nanoarchitectonics Research Centre at the National Institute of Advanced Industrial Science and Technology (AIST) in 2001. The concept of nanoarchitectonics first emerged in Tsukuba, Japan, around 2000.

This is not a coincidence that occurred in a particular place; rather, it is a historical inevitability resulting from the progress of research. The term “nanoarchitectonics” emerged in various places around the world in the early 2000s. In 2003, a research center called Functional Engineered Nano Architectonics (FENA) was established at UCLA. Peidong Yang's group at the University of California, Berkeley, wrote a doctoral thesis titled “Toward metal nanoarchitectonics: Shape‐controlled synthesis and assembly of metal nanoparticles” (Franklin Jongmyung Kim, 2005). Jiaxing Huang is said to have suggested the use of the term nanoarchitectonics here.^[^ ^39^ ^]^ The term was first used in the title of an academic paper in 2003. Stefan Hecht, from Freie Universität Berlin, Germany, published a paper titled “Welding, organizing, and planting organic molecules on substrate surfaces—Promising approaches toward nanoarchitectonics from the bottom up”.^[^ ^40^ ^]^

As mentioned above, the term “nanoarchitectonics” was first coined in Tsukuba, Japan, by Masakazu Aono and others. However, similar developments were also occurring in the United States and Europe at the same time, reflecting the inevitable progress of research. Nanoarchitectonics is a research concept born out of necessity in the 21st century. It is a concept that succeeded nanotechnology, which emerged in the 20th century.

### 1.3 Objectives of This Review Article

Research into nanoarchitectonics, which began at the beginning of the 21st century, has a history of over 20 years. Taking this history into account, this review will explain the trends in nanoarchitectonics. As explained above, nanoarchitectonics is a comprehensive concept, and it is nearly impossible to cover everything about it. For this reason, it is not a good idea to summarize the past 20 years of nanoarchitectonics in a chronicle‐like manner. Therefore, this review will pick up several typical topics and explore their trends.

First, we will focus on the achievements of nanoarchitectonics in the field of ultrasmall‐scale systems. This involves the manipulation of atoms and molecules. This section will focus on the atomic switch in particular, as this converts atomic/molecular manipulation into device‐level functionality. Waser and Aono, the founders of nanoarchitectonics, play a central role in this technology.^[^ ^41^ ^]^ In this category, we will also examine local probe chemistry and on‐surface synthesis.^[^ ^42^ ^]^ This fusion of probe microscopy—a typical example of nanotechnology—and organic synthesis—a traditional field of materials chemistry—is a key development in nanoarchitectonics. This well represents the direction of nanoarchitectonics, which aims to combine different fields.

Organizing structures in layers is a very rational material construction method. The next section will therefore discuss layered assembly. A representative example of a core component structure is a 2D nanosheet.^[^ ^43^ ^]^ We will then discuss nanosheet technology. Another example of layered construction is LbL assembly. Although this technology appears to be well established,^[^ ^44^ ^]^ new developments are still ongoing. This section will explore the latest trends in LbL assembly.

Nanoarchitectonics takes place in a variety of media. The diversity of structures formed reflects the freedom of movement during this process. In the next section, we will explain nanoarchitectonics at liquid interfaces^[^ ^45^ ^]^ that exhibit these characteristics. This section will show how various substances transform into a wide variety of forms through the construction with high freedom at the liquid interface. In a slightly different and advanced system, we will discuss controls of living cells at liquid interfaces to demonstrate their high potential in complex systems.^[^ ^46^ ^]^ Thus, we demonstrate that liquid interfaces are useful not only for simple substances but also for complex functional systems like living cells.

In addition to these standard topics, the next section provides an overview of the scope of nanoarchitectonics and explores how this concept can advance materials science. It also touches upon some recently emerging topics. The final section draws on these discussions to explore the challenges and future directions of nanoarchitectonics.

## 2 Atomic/Molecular Manipulation

### 2.1 Atomic Switch

The creation of devices that control the movement and arrangement of atoms is a clear demonstration of the effectiveness of nanoarchitectonics, which involves building functional systems from the nanoscale. Atomic switches are a prime example of this concept. These are nanoscale devices that switch on and off by controlling the movement of several atoms. Atomic switches are typically operated by controlling the movement of metal atoms using the properties of a material called a solid electrolyte. Thanks to their small size, low power consumption, non‐volatility, and radiation resistance, atomic switches are expected to find application in a variety of fields. Potential applications may include IoT devices and radiation environments such as outer space and medical settings.

Terabe et al.'s pioneering paper outlines the principles of the atomic switch.^[^ ^47^ ^]^ The device involves creating a gap of approximately 1 nm between a solid electrolyte electrode and a metal electrode. The device is operated by controlling the formation and annihilation of metal clusters within this gap. When a negative voltage is applied to the metal electrode, metal atoms precipitate from the surface of the solid electrolyte electrode, forming clusters of metal between the two electrodes and turning the switch on. Conversely, when a positive voltage is applied to the metal electrode, the precipitated metal atoms dissolve into the solid electrolyte electrode, causing the metal clusters to disappear and turning the switch off. This new principle is expected to accelerate the development of computer electronics with this new concept.

Atomic switch systems are being used not only for simple switching functions, but also for devices that mimic synapses in living organisms. In pioneering research, Ohno, Hasegawa, and their colleagues developed a synaptic device in which the frequency of the electrical signal input controls the conductance.^[^ ^48^ ^]^ In this study, they applied a small pulse voltage to mimic the action potential observed during synaptic activity in the brain. Inputting a small pulse voltage to the synaptic device formed unstable silver atomic bridges that then naturally disappeared. When a pulse voltage was applied at a low frequency, for example, every 20 s, the conductance temporarily increased and then decreased toward the initial value. This behavior corresponds to short‐term plasticity. Conversely, when a pulse voltage was applied at a high frequency, such as every 2 s, the increased state of conductance persisted for an extended period. This corresponds to long‐term potentiation. An atomic switch whose switching mechanism is based on the formation of an atomic cluster bridge may be able to mimic synaptic functions that are based on chemical signals.

**Figure 2:**
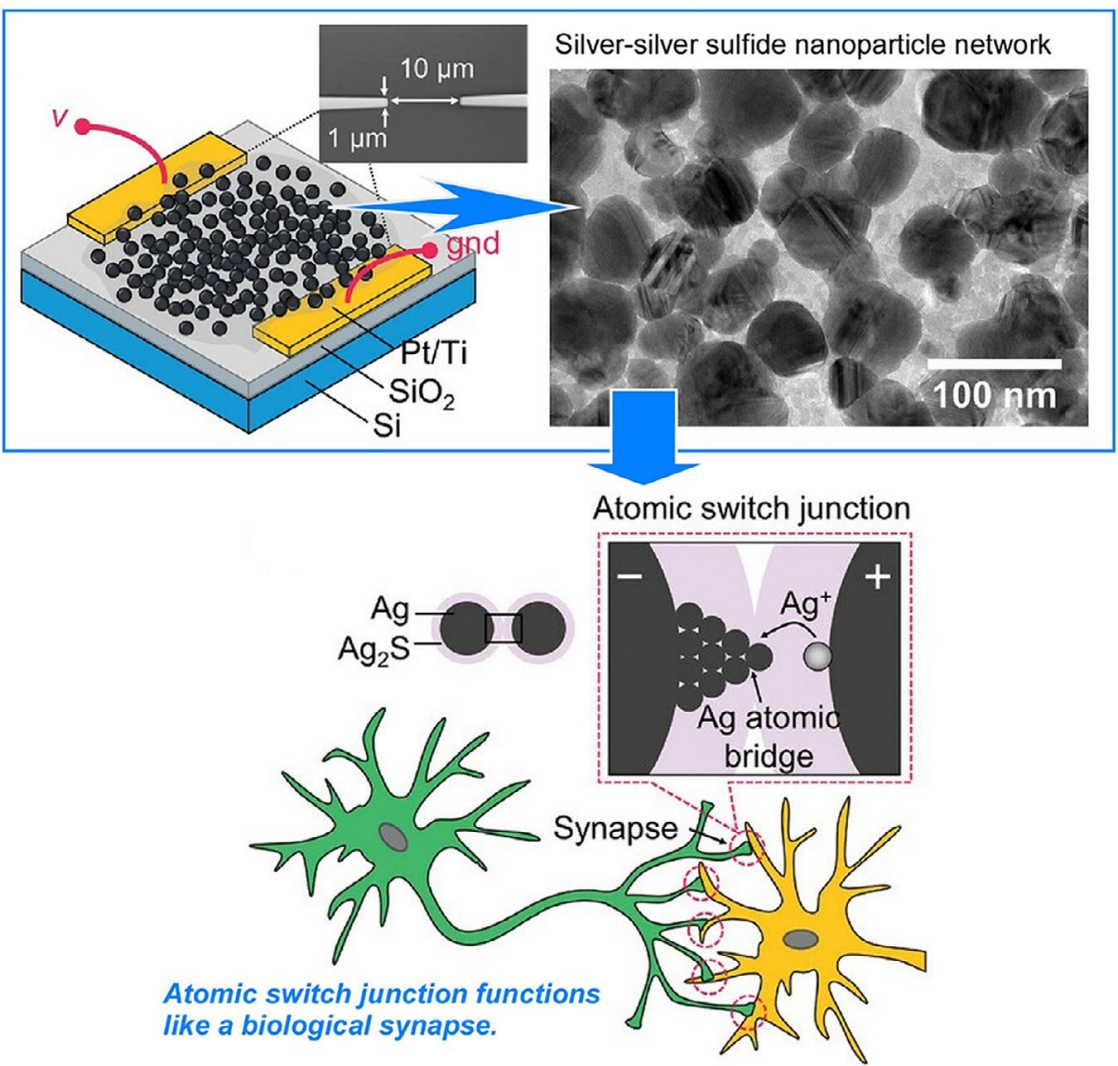
Construction of self‐assembled silver–silver sulfide nanoparticle networks of atomic switches to mimic biological synapses. Reproduced under terms of the CC‐BY license from Ref. [49], 2024 Wiley‐VCH.

Srikimkaew et al. applied the artificial synapse organization to a network system (Figure 2).^[^ ^49^ ^]^ They demonstrated that a self‐assembled silver‐silver sulfide nanoparticle network, based on atomic switches, can mimic the plasticity of biological synapses. The nanoparticles organized themselves into a network comprising over 10^3^ randomly interconnected atomic switch interfaces. Exploiting the collective dynamics of switch networks offers unique advantages. This approach enables the exploration of collective behaviors and emergent properties that are not evident in individual elements, facilitating the development of more versatile memory and learning functions. The device can perform functions similar to those of biological synapses, such as short‐term plasticity, paired‐pulse facilitation, and long‐term potentiation. Repetitive pulse stimulation can induce a transition from short‐term plasticity to long‐term potentiation, a process that is associated with learning and memory formation. The transient change in short‐term plasticity is accentuated by a spontaneous relaxation effect due to its volatility. After stimulation, the conductance of the device relaxed to its initial value with a constant decay time, while the steady‐state conductance increased. Conductance potentiation occurred when the interval between pulses was shorter than the relaxation time, corresponding to short‐term synaptic facilitation. Prolonged stimulation could extend the retention time of the device, comparable to the transition from short‐term plasticity to long‐term potentiation in biological systems. For instance, the lifetime of long‐term potentiation following 100 pulses of stimulation was as long as 40 min. These behaviors mimic the device's ability to forget newly acquired information after long‐term storage, in a manner similar to human memory.

**Figure 3:**
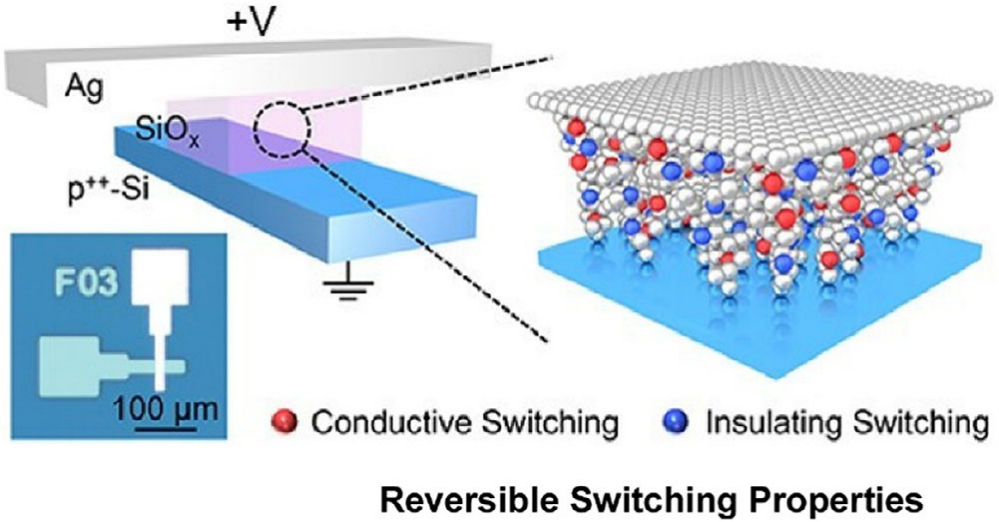
Ag‐based filamentary memristor: vertical cross‐point structure with active Ag and inactive p++‐Si electrodes sandwiching a 2.7‐nm‐thick SiOx dielectric film to exhibit reversible switching properties, which are attributed to the formation and rupture of nanoscale Ag conductive filaments. Reprinted with permission from Ref. [50] Copyright 2023 American Chemical Society.

Resistive random access memory is a key technology for data storage and neuromorphic computing. Its core is based on nanoscale conductive filament dynamics. The memristor studied by Ge and colleagues employs a vertical cross‐point structure, with active Ag and inactive p^++^‐Si electrodes sandwiching a 2.7‐nm‐thick SiO_x_ dielectric film (Figure 3).^[^ ^50^ ^]^ This memristor exhibits reversible switching properties, which are attributed to the formation and rupture of nanoscale Ag conductive filaments. Ag‐based filamentary memristors exhibit relatively constant conductance, accompanied by significant current fluctuations following soft breakdown. A nanoscale percolation network forms where the insulating and conductive switching of Ag atoms is dynamically balanced. These switching events collectively occur as an avalanche, consistent with avalanche criticality. Remarkably, these atomic switching events follow scale‐free avalanche dynamics, with exponents that satisfy the criticality criterion. This work builds on the results obtained from memristor‐based electronic synapses, artificial neurons, and other neuromorphic devices. Combining these results will contribute to the development of low‐cost, biologically plausible neuromorphic systems.

**Figure 4:**
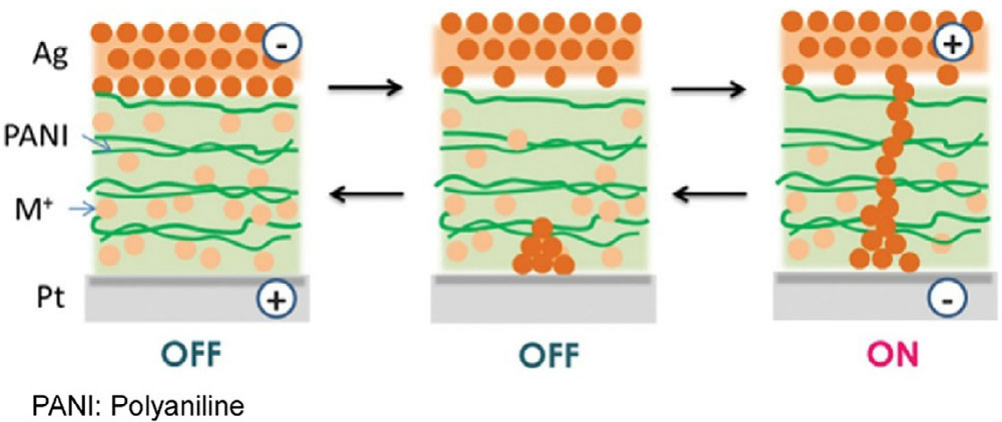
Metal‐ion‐doped devices consisting of Ag/metal‐ion‐doped polyaniline/Pt sandwich structures to exhibit repeated resistive switching between high (on) and low (off) conductance states in the presence of both Ag^+^ and Cu^2+^ ion doping. Reproduced under terms of the CC‐BY license from Ref. [51], 2023 Taylor & Francis.

The nanoscale structure and resulting neuromorphic properties of polyaniline‐based atomic switches were also explored. This system offers a new physical foundation for developing next‐generation nanoarchitectonics‐based computing systems. Gimzewski, Stieg, and their colleagues fabricated metal‐ion‐doped devices consisting of Ag/metal‐ion‐doped polyaniline/Pt sandwich structures using an in situ wet process (Figure 4).^[^ ^51^ ^]^ These devices exhibited repeated resistive switching between high (on) and low (off) conductance states in the presence of both Ag^+^ and Cu^2+^ ion doping. The memory capacity of these devices was monitored over time. By adjusting the duration of the “on” state using the applied bias voltage, both short‐term and long‐term memory were achieved. Evidence of memristor behavior and quantized conductance was also observed, which was interpreted in terms of metallic filaments forming bridges between the metal‐doped polymer layers. This class of metal‐doped polymers could facilitate the fabrication of neuromorphic building blocks while maintaining compatibility with modern complementary metal‐oxide‐semiconductor (CMOS) processes and hardware technologies.

**Figure 5:**
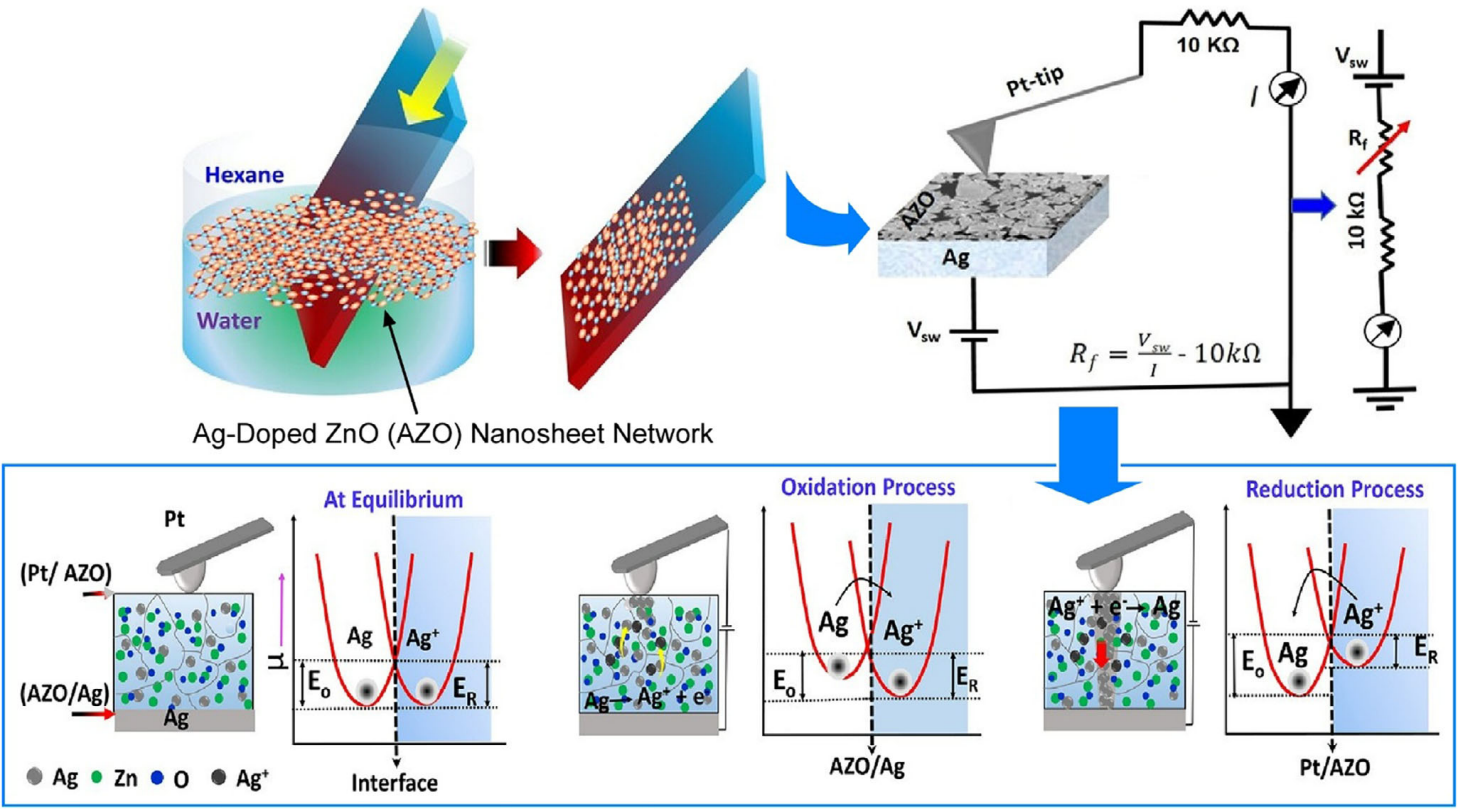
Preparation of uniform, large‐area films of Ag‐doped nanosheet networks through self‐assembly at the hexane–water interface with approximately 1 µm in size and 60–100 nm in thickness for atomic switch operation with Pt/Ag‐doped ZnO/Ag structures, creating a conducting path for “on” state. Reprinted with permission from Ref. [52] Copyright 2023 American Chemical Society.

The production‐based development of nanoarchitectonics for atomic switches is also being considered. The development of a simple, inexpensive method of obtaining large‐area 2D semiconducting nanosheets would be highly desirable for use in mass‐produced devices. Kumar, Nayak, and colleagues reported a method of fabricating Ag‐doped ZnO nanosheet networks through self‐assembly at the hexane–water interface.^[^ ^52^ ^]^ They demonstrated how to produce uniform, large‐area films of these networks through self‐assembly at the hexane–water interface by controlling the solute/solvent ratio (Figure 5). These self‐assembled films consist of uniformly tiled nanosheets measuring approximately 1 µm in size and 60–100 nm in thickness. Using these films in Pt/Ag‐doped ZnO/Ag structures achieves atomic switch operation. Reduced Ag atoms nucleate and form filaments towards the Ag electrode, creating a conducting path and entering the “on” state. When the polarity of the bias voltage is reversed, the state is turned off. In general, liquid–liquid interface‐assisted self‐assembly provides networked nanosheet films with a single‐layer tiling structure more effectively than conventional methods such as drop casting or spin coating.

**Figure 6:**
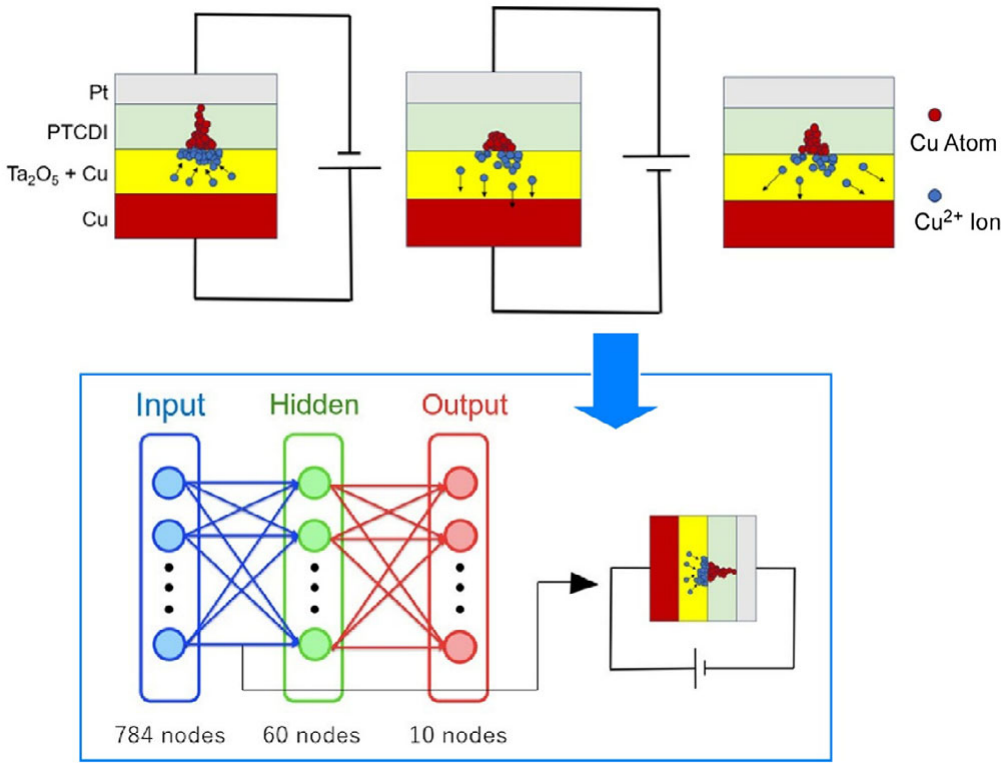
Simulation of the effect of the characteristics of atomic switches on the accuracy and power consumption of deep learning: As using a network consisting of an input layer (784 nodes), a hidden layer (60 nodes), and an output layer (10 nodes). Reprinted with permission from Ref. [53] Copyright 2024 IOP Science.

In terms of cutting‐edge science and technology, the application of atomic switches and neuromorphic devices to artificial intelligence is also being considered. Hardware‐based deep learning using neuromorphic elements is attracting a great deal of attention as an alternative to standard von Neumann computational architecture. Atomic switches can learn and can be used as synaptic devices for deep learning. However, concerns have been raised about using atomic switches in this field, including inaccuracies in resistance control and the autonomous decay of weights. These characteristics may cause unintended changes in weights during the learning process. Tomatsuri and Hasegawa simulated the effect of the characteristics of atomic switches on the accuracy and power consumption of deep learning.^[^ ^53^ ^]^ As shown in Figure 6, they adopted a network consisting of an input layer, a hidden layer, and an output layer. The input layer comprises 784 nodes representing pixels, while the output layer comprises 10 nodes, each corresponding to a digit. A single hidden layer consisting of 60 nodes was used. They found that weight decay, a unique atomic switch characteristic, has the potential to counteract neural network degradation.

### 2.2 Local Probe Chemistry

The atomic switch mentioned above is an example of the development of device functions through nanoarchitectonics, which relies on the manipulation of atoms. Another form of nanoarchitectonics focuses on the manipulation of molecules rather than atoms. One example of this is local probe chemistry, which uses the tip of a probe microscope to control organic reactions. Another approach is on‐surface synthesis, whereby molecules are synthesized on a surface while being observed with a probe microscope. These approaches can be considered a fusion of nanotechnology and organic chemistry. In this section, we will discuss nanoarchitectonics based on the manipulation of molecules.

Recent advances in state‐of‐the‐art probe microscopy have enabled the observation of single‐molecule chemical reactions and the direct observation of the internal structure of the products via tip‐induced reactions. Kawai, Kubo, Foster, and colleagues performed local probe chemical reactions using an atomic force microscopy (AFM) tip to synthesize three‐dimensional graphene nanoribbons, demonstrating their potential as a site for local probe chemical reactions through the use of tip‐induced assembly.^[^ ^54^ ^]^ By applying a bias voltage, the out‐of‐plane C─Br σ bond was broken to produce radical species. These radical species were then mechanically terminated using a single bromine atom and a C_60_ fullerene molecule. These processes were carried out while recording the tunnelling current. Quantum chemical calculations confirm that C_60_ molecules can be easily manipulated by scanning at lower voltages. This makes it an ideal platform for local probe chemistry. Long‐lived radicals can be obtained via the tunnelling current and stabilized by bromine atoms or C_60_ molecules. Such direct addition reactions are essential for synthesizing single compounds in precise, site‐specific ways.

**Figure 7:**
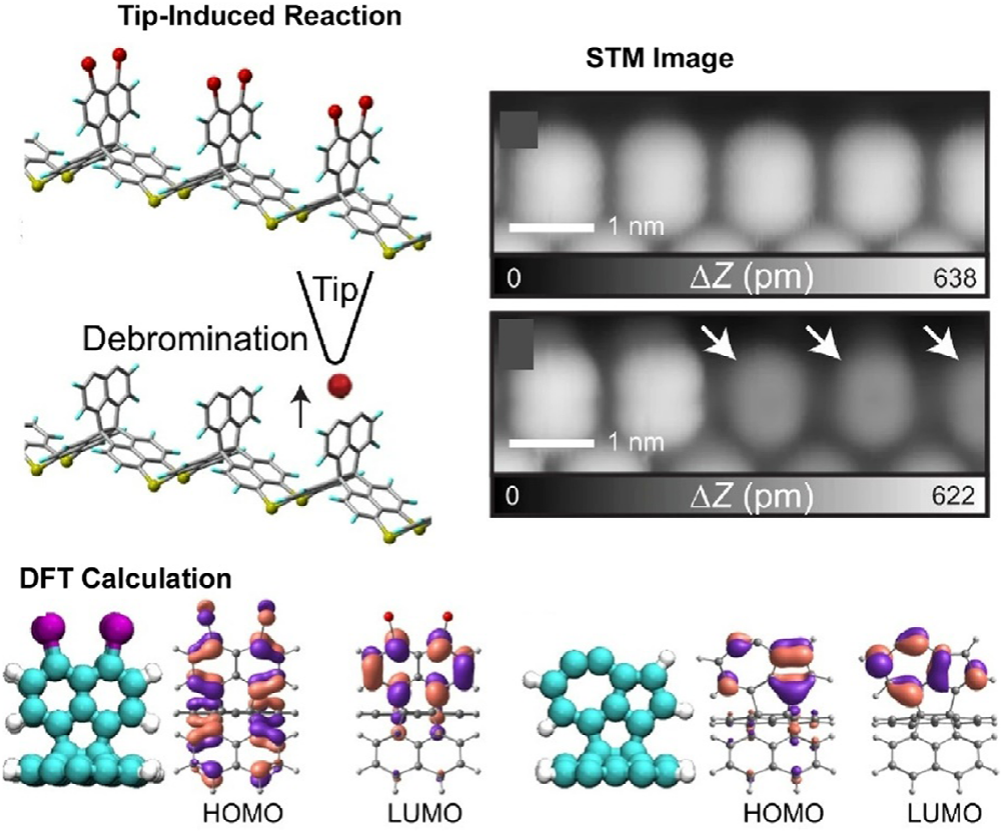
Formation and properties of dehydroazulene isomers and diradical units within three‐dimensional organometallic compounds on an Ag(111) surface using a combination of low‐scanning tunnelling microscopy (STM) and density functional theory (DFT) calculations. Reproduced under terms of the CC‐BY license from Ref. [55], 2023 Springer‐Nature.

One‐dimensional molecular arrays containing stereoisomers are difficult to synthesize. However, these structures have great potential for use in optical, electronic, and magnetic molecular devices. Kawai, Kubo, Foster, and their colleagues used a combination of low‐scanning tunnelling microscopy (STM) and density functional theory (DFT) to study the formation and properties of dehydroazulene isomers and diradical units within three‐dimensional organometallic compounds on an Ag(111) surface.^[^ ^55^ ^]^ The C─Br bond protruding from the surface was examined in detail using a combination of low‐temperature STM and DFT calculations (Figure 7). First, a tip‐induced voltage pulse forms a diradical species via the homolytic decomposition of two C─Br bonds in the naphthyl group. This is then transformed into a chiral dehydroazulene moiety. The electronic properties of the resulting radical species, obtained by sequentially removing bromine atoms using tunnelling current, were investigated using scanning tunnelling spectroscopy (STS). The diradical species is energetically unstable during debromination and therefore undergoes immediate isomerization to form dehydroazulene. The reaction rate can be adjusted by altering the tip‐sample gap, enabling the array units to be switched between three diradical configurations and two dehydroazulene structures in a locally probed, controlled manner. The chiral dehydroazulene and diradical structures can be switched by controlling the application of bias voltage. Furthermore, the diradical site has been found to host an open‐shell singlet that undergoes an inelastic spin transition from antiferromagnetic to ferromagnetic coupling. Therefore, by extending the local chemical reactions analyzed at the single‐molecule level, they can control the unit structures within the molecular array. In addition to tip‐induced stereoisomerization, this systematic isomerization is important for developing nanochemistry for constructing molecular systems on a molecule‐by‐molecule basis.

**Figure 8:**
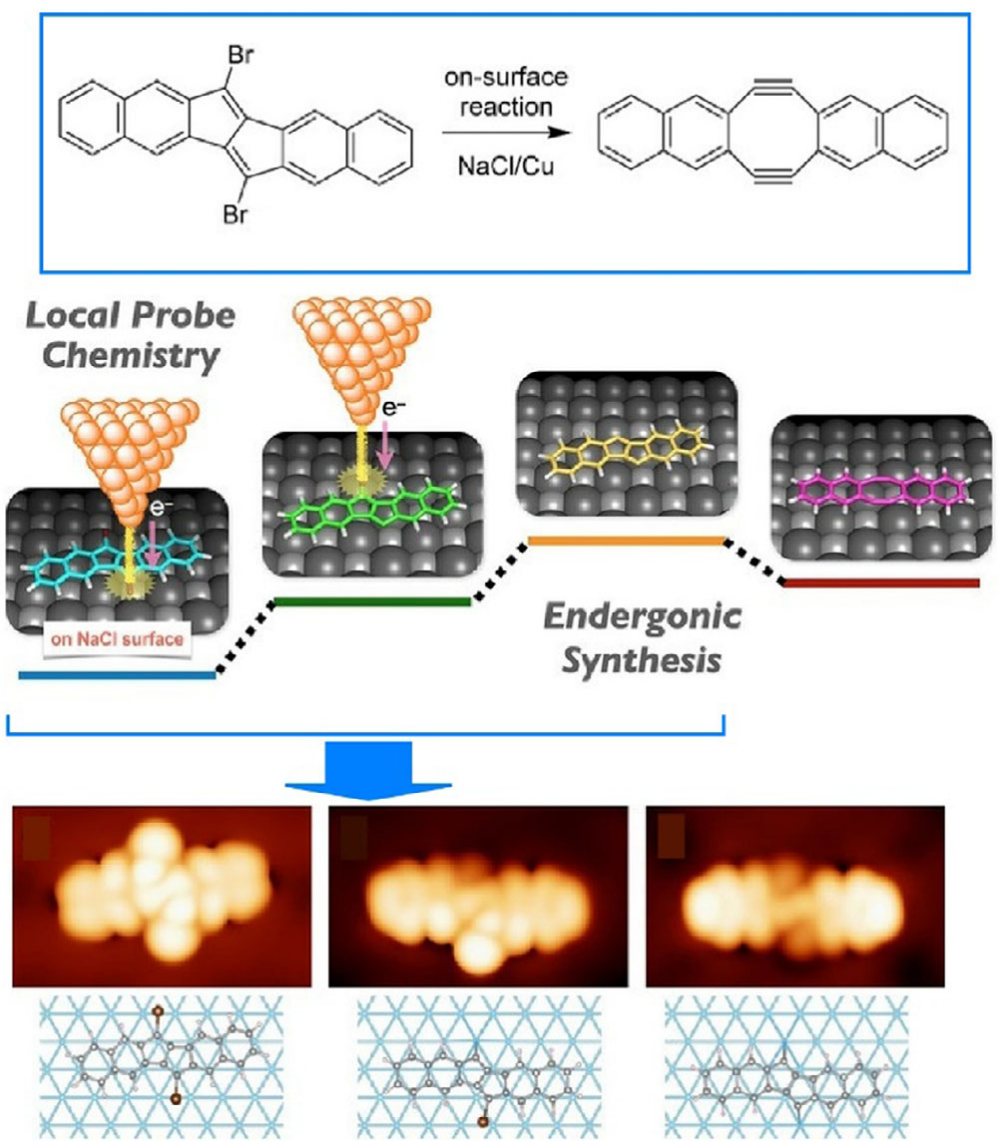
An energy absorption reaction synthesizing the Sondheimer–Wong diyne analogue (6,7,14,15‐tetradehydrocycloocta[1,2‐b:5,6‐b“]dinaphthalene) from (6,13‐dibromopentaleno[1,2‐b:4,5‐b”]dinaphthalene), which can be observed at the molecular level by detecting a significant change in contrast in the STM topography. Reprinted with permission from Ref. [56] Copyright 2020 Wiley‐VCH.

The surface scanning probe microscopy (SPM) approach allows local probe reactions to synthesize and analyze unstable, short‐lived products, which are useful for exploring nanoarchitectonics processes at the molecular level. Kawai, Kantorovich, Ito, and colleagues investigated local probe reactions on a thin NaCl film on a copper (Cu(111)) surface at 4.3 K.^[^ ^56^ ^]^ Specifically, they observed an energy absorption reaction synthesizing the Sondheimer–Wong diyne analogue (6,7,14,15‐tetradehydrocycloocta[1,2‐b:5,6‐b“]dinaphthalene) from (6,13‐dibromopentaleno[1,2‐b:4,5‐b”]dinaphthalene). This multistep reaction is an overall electron absorption reaction. It is driven by the temporal charging and discharging of molecules positioned at the nanometer‐scale junction between the Cu substrate and the Cu tip, located just beneath the ultrathin NaCl film. Such nanoarchitectonics at the molecular level can be observed by detecting a significant change in contrast in the STM topography (Figure 8). The disappearance of the spot induced by the Br atom and the sudden change in current each correspond to a single debromination. Two successive debrominations induced by the tip lead to the formation of a diradical with dangling bonds. It has been demonstrated that the second debromination frequently occurs immediately following the first. The structures of the synthesized compounds were identified by combining high‐resolution differential conductance maps and DFT calculations. The corresponding STM simulation images were consistent with the experimental results, and the detailed processes were identified. A plausible mechanism was suggested whereby the reaction is promoted by briefly charging the molecule negatively via tunnelling current. At the same time, the energies of the debrominated species and the Sondheimer–Wongein species are significantly lowered relative to the negatively charged molecular species. While the reaction direction is generally reversed in solution chemistry, low‐temperature local chemistry offers the unique flexibility to control the radical state and resulting Sondheimer‐Wongein formation.

**Figure 9:**
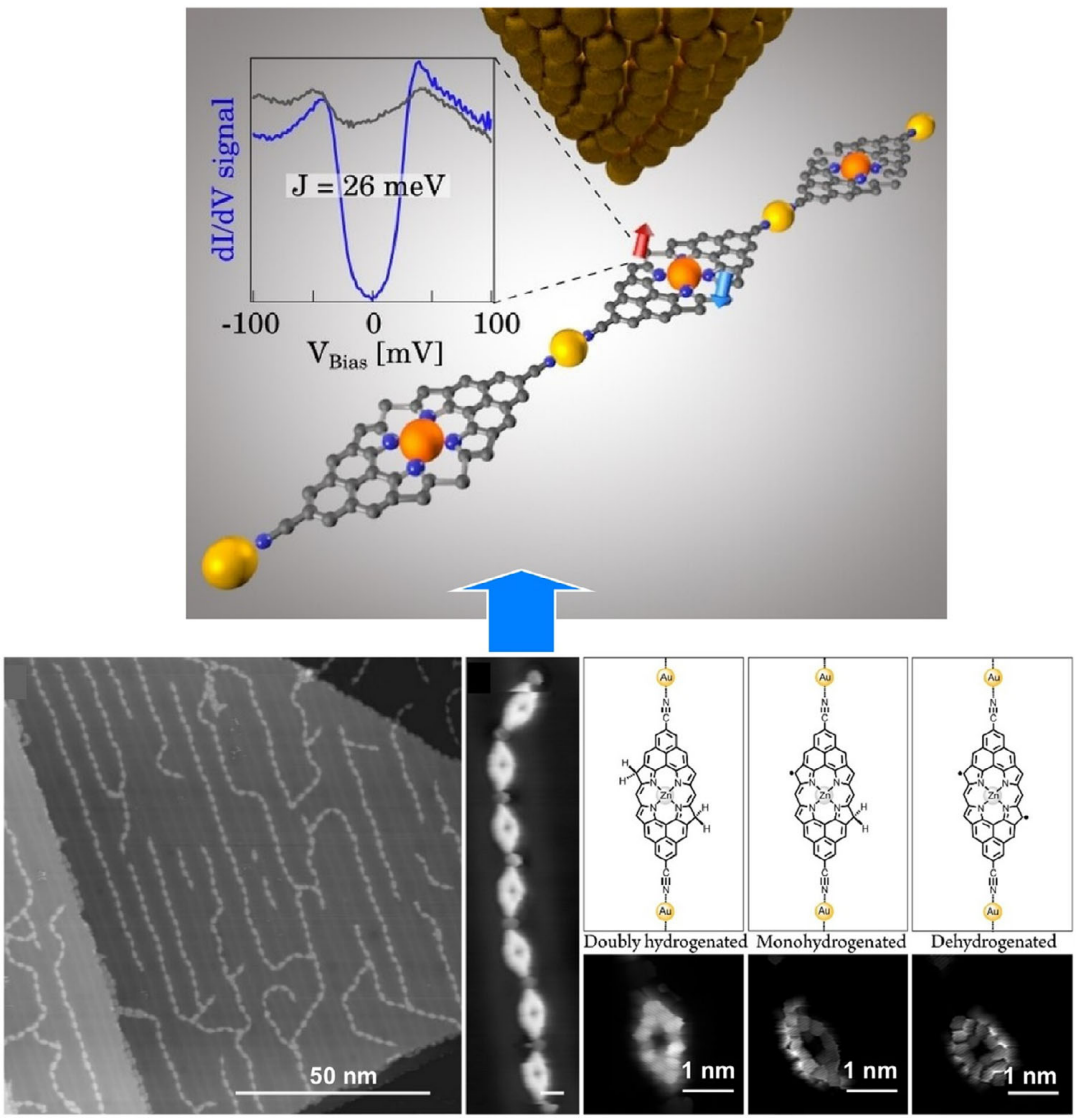
Combination of surface synthesis and coordination chemistry for molecular nanoarchitectonics of π‐electron magnetic porphyrin species, where the formation of Au–CN‐coordinated porphyrin monomers, covalent porphyrin dimers, and one‐dimensional porphyrin polymers was revealed by a combination of STM studies and theoretical calculations. Reproduced under terms of the CC‐BY license from Ref. [57], 2024 Wiley‐VCH.

Magnetic compounds containing π‐electrons on surfaces can be a powerful platform for probing spin interactions at the atomic level. The organization of these compounds and the elucidation of their magnetic properties are key research objectives. To this end, Tenorio et al. employed a combination of surface synthesis and coordination chemistry to examine the molecular nanoarchitectonics of π‐electron magnetic porphyrin species.^[^ ^57^ ^]^ Combined SPM, spectroscopy, and theoretical simulations produced organometallic porphyrin polymers with open‐shell structures (Figure 9). A porphyrin precursor containing a trans‐configured carbonitrile group was deposited on an Au(111)/mica surface and converted into a nonmagnetic species via a surface‐promoted reaction. The formation of Au─CN‐coordinated porphyrin monomers, covalent porphyrin dimers, and one‐dimensional porphyrin polymers was revealed by a combination of STM studies and theoretical calculations. No magnetic exchange coupling was observed between adjacent units, indicating that laterally condensed porphyrin units can be converted to open‐shell species that do not exhibit interunit magnetic exchange interactions, yet still retain the magnetic properties of the building blocks. These findings pave the way for new developments in nanomagnetism and coordination chemistry, particularly in the design of metal–organic nanostructures containing π‐electron magnets.

**Figure 10:**
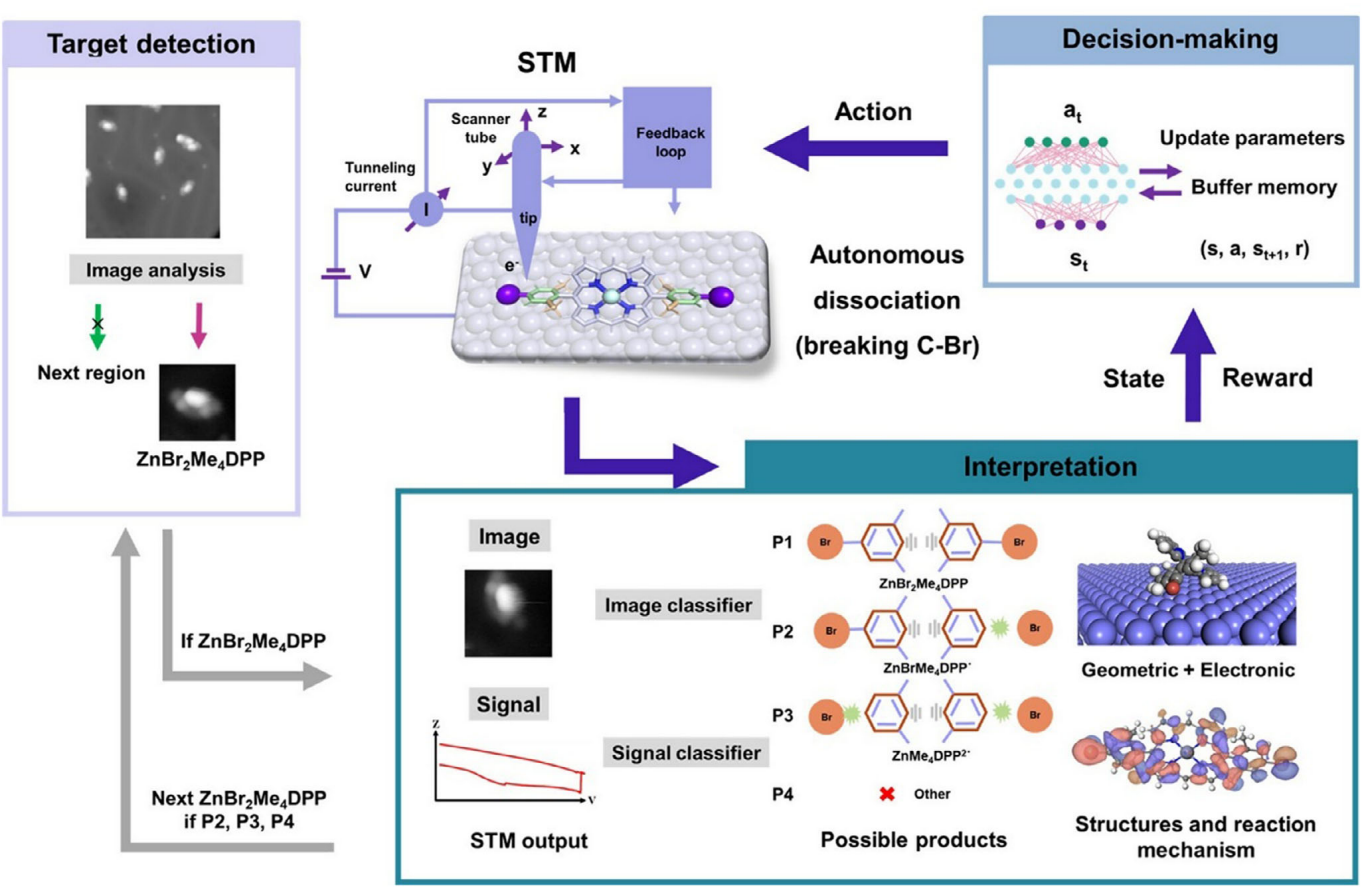
Example scheme of auto on‐surface synthesis consists of three key modules: target detection, decision‐making, and interpretation. Reproduced under terms of the CC‐BY license from Ref. [58], 2024 American Chemical Society.

Artificial intelligence is also being introduced to the field of molecular nanoarchitectonics on surfaces. Controlling chemical reactions and providing a route to precise atomic and molecular construction is challenging, requiring efficient and autonomous SPM techniques to learn optimal strategies. Wu, Liljeroth, Foster, and their colleagues developed software to automate the removal of bromine from hundreds of Zn(II)‐5,15‐bis(4‐bromo‐2,6‐dimethylphenyl)porphyrins on Au(111).^[^ ^58^ ^]^ The system uses a neural network model to interpret STM output and a deep reinforcement learning model to optimize operating parameters (Figure 10). The system consists of three components: a target detection component that searches for and identifies target fragments based on STM images; a model that interprets STM output during operation; and a deep reinforcement learning agent that makes decisions and selects SPM parameters. Analysis of the large data sets accumulated from experiments enables hidden physical information to be discovered, 3D molecular structures to be explored, and reaction mechanisms to be elucidated. Improved tips, optimized bias voltage patterns, or incorporating AFM signals into the workflow to provide atomic‐resolution scan images could potentially enhance the selectivity and accuracy of the models.

In this section, we present some examples of nanoarchitectonics involving the manipulation of atoms and molecules. The atomic switch is a prime example of nanoarchitectonics, in which the movement of atoms and the formation of clusters determine the function of the device. Research into the atomic switch was initiated and developed in the laboratory of Aono et al., who are proponents of nanoarchitectonics. Consequently, the history of the atomic switch coincides with the development of nanoarchitectonics. Local probe chemistry and on‐surface chemistry, presented as another category, enable organic synthesis to be controlled, observed, and interpreted as real images using chip technology—a typical nanotechnological process. This fusion of nanotechnology and organic chemistry reflects the idea of nanoarchitectonics. Interestingly, research themes related to artificial intelligence are emerging in the latest research on both atomic switches and on‐surface synthesis. It is thought that artificial intelligence's involvement in manipulating atoms and molecules, and thus in nanoarchitectonics, is becoming apparent.

## 3 Layered Assembly

When considering an unlimited number of nanounits, assembling them within free spaces into functional materials is not always easy. One way to simplify this process is to reduce dimensionality. In other words, an LbL organization strategy is easier to devise than a three‐dimensional one. In this context, organizing 2D materials by stacking layers appears to be a more effective approach. Accordingly, research into the high functionality of 2D materials and LbL organization is very active and plays an important role in nanoarchitectonics. In this section, we will discuss two representative examples of this research: nanosheet technology and LbL assembly.

### 3.1 Nanosheet Technology

**Figure 11:**
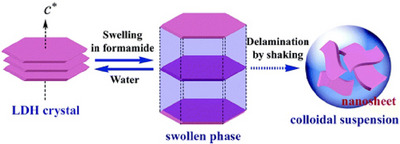
Investigation into the synthesis, anion exchange and exfoliation of cobalt‐aluminium layered double hydroxide (LDH) leading to uniform, large, hexagonal Co–Al–CO3 LDH platelets using the homogeneous precipitation method with urea hydrolysis under reflux conditions. Reprinted with permission from Ref. [60] Copyright 2006 American Chemical Society.

Various 2D materials, ranging from familiar substances to novel ones, can be regarded as nanosheets.^[^ ^59^ ^]^ As basic nanosheet technology, Sasaki and his team conducted a systematic investigation into the synthesis, anion exchange, and exfoliation of cobalt‐aluminium layered double hydroxide (LDH) (Figure 11).^[^ ^60^ ^]^ They also prepared LDH nanosheet composite membranes using the LbL assembly method with the aid of polyanion, exploring their properties. First, they synthesized uniform, large, hexagonal Co–Al–CO_3_ LDH platelets using the homogeneous precipitation method with urea hydrolysis under reflux conditions. They found that the exchange products of various anion forms exhibit different exfoliation behaviors in formamide. LDH exfoliation follows two separate processes: rapid swelling, followed by gradual exfoliation. Swelling occurs instantly, and exfoliation of the highly swollen phase proceeds gradually through continuous vibration. Exfoliation yielded a transparent, pink suspension containing well‐defined nanosheets with lateral sizes of up to 2 µm. The nanosheets were then layered onto a quartz glass substrate using an anionic polymer, poly(sodium styrene‐4‐sulfonate). Magnetic circular dichroism measurements revealed that the Co‐Al LDH nanosheets behaved as ferromagnetic nanolayers at room temperature and that the multilayer structure exhibited an exceptional magneto‐optical response in the ultraviolet–visible spectrum. This Co–Al–CO_3_ LDH synthesis method can also be extended to other transition metal‐containing LDHs (e.g., Fe–Al, Zn–Al, Ni–Al) to produce large platelet crystals.

Reduced graphene oxide is another widely used nanosheet material.^[^ ^61^ ^]^ For instance, Nguyen, Ruoff, and their colleagues reduced a colloidal suspension of exfoliated graphene oxide sheets using hydrazine hydrate.^[^ ^62^ ^]^ Reducing the exfoliated graphene oxide sheets with hydrazine in water produced a material whose graphitic properties were comparable to those of pure graphite. At the nanoscale, this carbon‐based material comprises thin graphene sheets and has a high surface area. The hydrazine treatment creates unsaturated and conjugated carbon atoms, which can conduct electricity. Reduced graphene oxide sheets are being explored for various applications.

**Figure 12:**
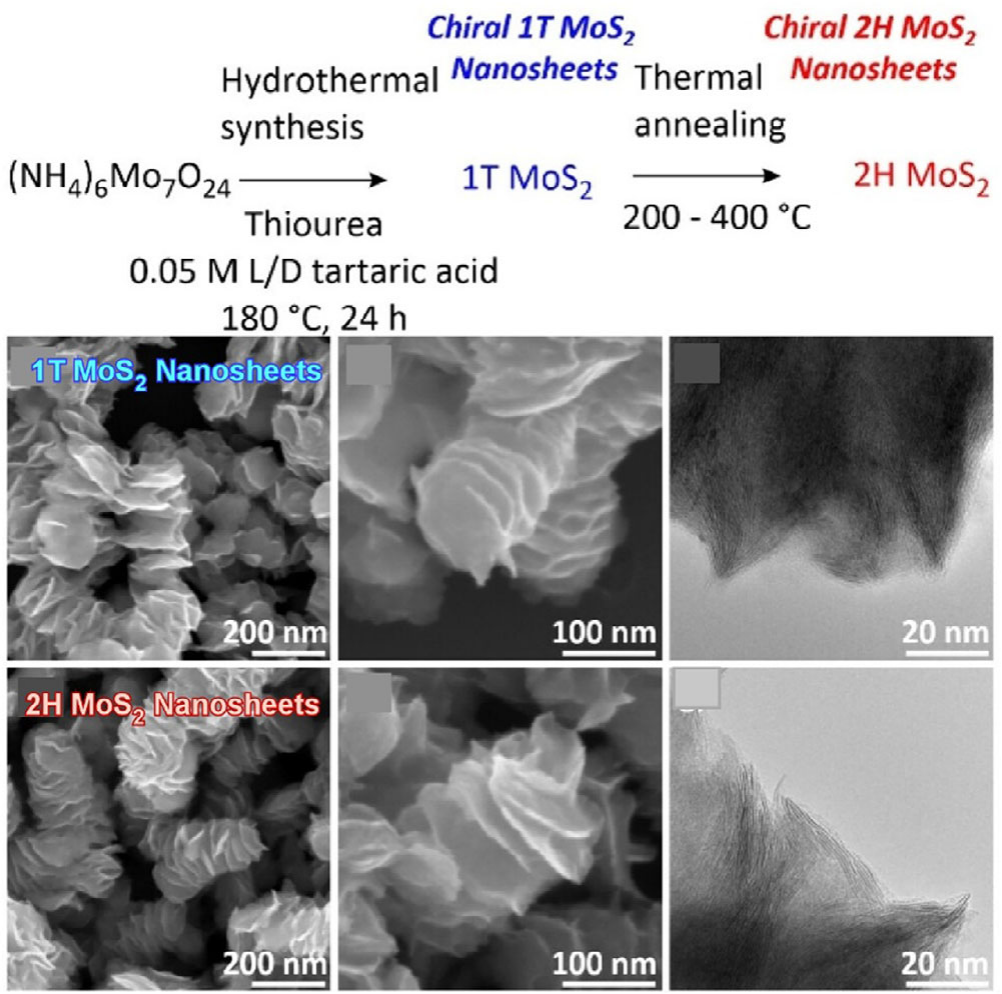
Produced semiconducting 2H molybdenum disulfide (MoS_2_) nanosheets using phase engineering‐based nanoarchitectonics, where the chiral, metastable, metallic 1T‐MoS_2_ nanosheets were converted into the thermodynamically stable 2H phase via a thermal annealing process that was specifically designed to maintain the chiral morphology during the metallic‐to‐semiconductor phase transition. Reprinted with permission from Ref. [63] Copyright 2025 Wiley‐VCH.

In addition to traditional nanoarchitectonics involving nanosheets, more advanced techniques such as controlling the chirality of the structure have been employed in recent years. Branzi, Gun'ko, and their colleagues have produced semiconducting 2H molybdenum disulfide (MoS_2_) nanosheets with high chiroptical activity using phase engineering‐based nanoarchitectonics (Figure 12).^[^ ^63^ ^]^ MoS_2_ is a transition metal dichalcogenide with promising properties for a wide range of applications, from optoelectronics to spintronics. The first step involved preparing metastable metallic 1T MoS_2_ nanosheets with chiral morphology by hydrothermal synthesis. When these nanocrystals were grown under hydrothermal conditions in the presence of tartaric acid, symmetry breaking was controlled, causing the nanosheets to grow along the screw dislocation axis and form nanorods. These chiral, metastable, metallic 1T MoS_2_ nanosheets were then converted into the thermodynamically stable 2H phase via a thermal annealing process that was specifically designed to maintain the chiral morphology during the metallic‐to‐semiconductor phase transition. This method enables the fabrication of semiconducting 2H chiral nanosheets while preserving the chiral morphology of the initial 1T MoS_2_ precursor. This method paves the way for developing new synthetic methods for nanoarchitectonics for various chiral nanomaterials. Further advances can be made in designing new transition metal dichalcogenide‐based chiral nanostructures with tunable chiroptical properties.

**Figure 13:**
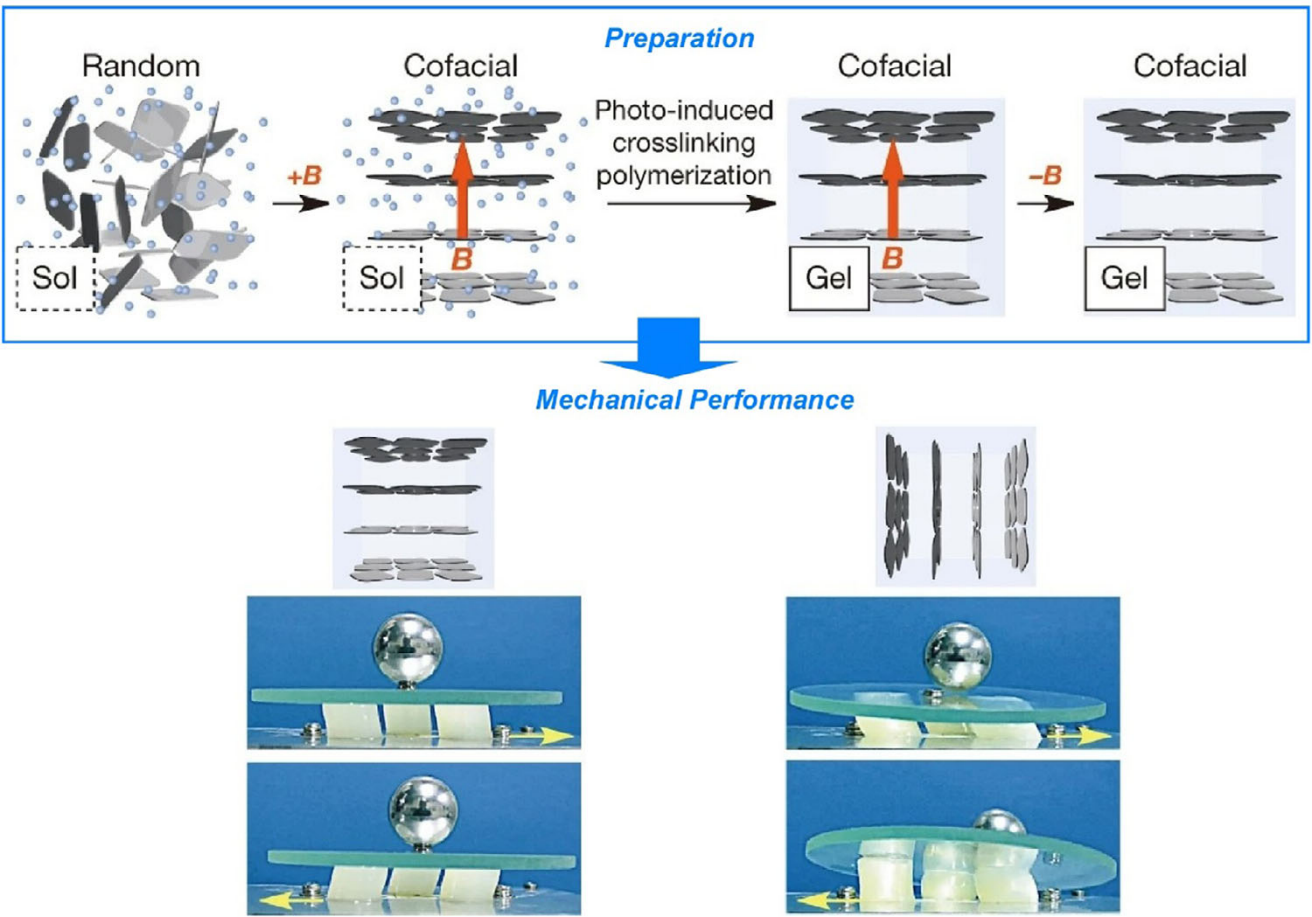
Composite hydrogels with anisotropic mechanical properties, which are governed by the electrostatic repulsion of the negatively charged monolayer titanate nanosheets embedded within them, with exposure to a strong magnetic field, in which planar‐oriented charged nanosheets embedded in this hydrogel reduce friction in one direction while increasing it in the perpendicular direction, providing excellent vibration isolation. Reprinted with permission from Ref. [64] Copyright 2014 Springer‐Nature.

A fundamental feature of nanosheets is their structural anisotropy. This can be reflected in the mechanical properties of materials that incorporate them. Ishida, Aida, and their colleagues developed composite hydrogels with anisotropic mechanical properties, which are governed by the electrostatic repulsion of the negatively charged monolayer titanate nanosheets embedded within them (Figure 13).^[^ ^64^ ^]^ When exposed to a strong magnetic field, the nanosheets align. This induces quasicrystalline structural order with uniformly large interplanar nanosheet spacing over macroscopic length scales. The nanosheets are easily deformed by shear forces applied parallel to them, but resist compressive forces applied perpendicular to them. This mechanism differs from that of many other polymer‐based composites, in which inorganic fillers interact with the polymer matrix to enhance mechanical properties. Conversely, the example studied here resembles articular cartilage, which achieves unparalleled functional efficiency by exploiting electrostatic repulsion to allow virtually frictionless mechanical movement within joints, even under high compression. Planar‐oriented charged nanosheets embedded in this hydrogel reduce friction in one direction while increasing it in the perpendicular direction, providing excellent vibration isolation. The hydrogel resists ion penetration and retains its mechanical anisotropy even when immersed in saline. It is therefore expected to be useful for potential biomedical applications.

**Figure 14:**
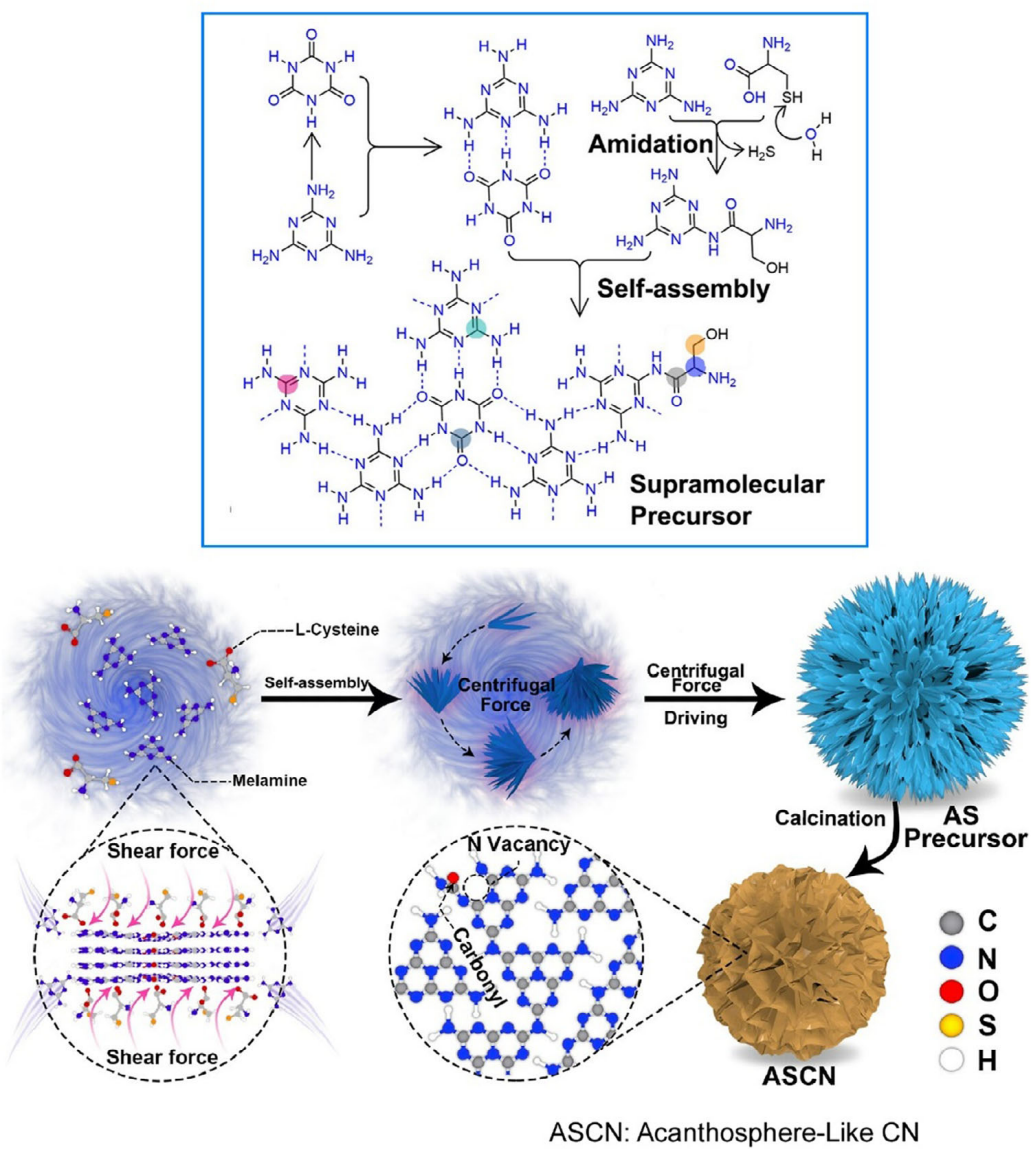
Preparation of acanthosphere‐like carbon nitride nanosheets containing nitrogen vacancies and functionalized with a carbonyl group using fluid shear stress‐induced molecular of melamine and L‐cysteine supramolecular precursors. Reprinted with permission from Ref. [65] Copyright 2023 American Chemical Society.

Polymeric carbon nitrides show promise as cocatalyst‐free composite photocatalysts. However, improvements are still needed in terms of catalytic sites and quantum efficiency. Jiao, Jiang, Fu, and colleagues reported a method of preparing acanthosphere‐like carbon nitride nanosheets containing nitrogen vacancies and functionalized with a carbonyl group using fluid shear stress‐induced molecular assembly (Figure 14).^[^ ^65^ ^]^ This provides an easy way to break down the layered stacks of nanosheets and build hierarchical assemblies for coupled photocatalysis. Fluid shear stress‐modulated self‐assembly of melamine and L‐cysteine produces acanthosphere‐like supramolecular precursors that are assembled in a thorn‐like configuration. Shear stress destroys the interlayer stacking interactions and separates the stacked structures into ultrathin layers. These layers are then reassembled into acanthosphere bundles by centrifugal force. This method has several advantages, including a high yield, a simple procedure, and the elimination of the need for intercalation reagents. The catalyst's ultrathin structure provides more exposed active sites and improves the separation of charge carriers. Enhanced activity and increased turnover numbers were observed in the reduction of oxygen to H_2_O_2_ and the oxidation of 4‐methoxybenzyl alcohol to anisaldehyde. This structure has several advantages. The optimized band structure provides effective light absorption and a suitable driving force for the catalytic reaction, thereby suppressing side reactions. Nitrogen vacancies and carbonyls induce electron redistribution and heterogeneity, promoting the spatial separation of charge carriers and leading to the formation of dual active sites. The assembly structure of ultrathin nanosheets facilitates the adsorption of reactive species, thereby improving charge transport and the durability of the combined photocatalyst. The nanoarchitectonics of these nanosheets, which utilize fluid shear stress, form ultrathin structures that maximize the utilization of electrons and holes in redox reactions, converting solar energy into valuable chemicals.

**Figure 15:**
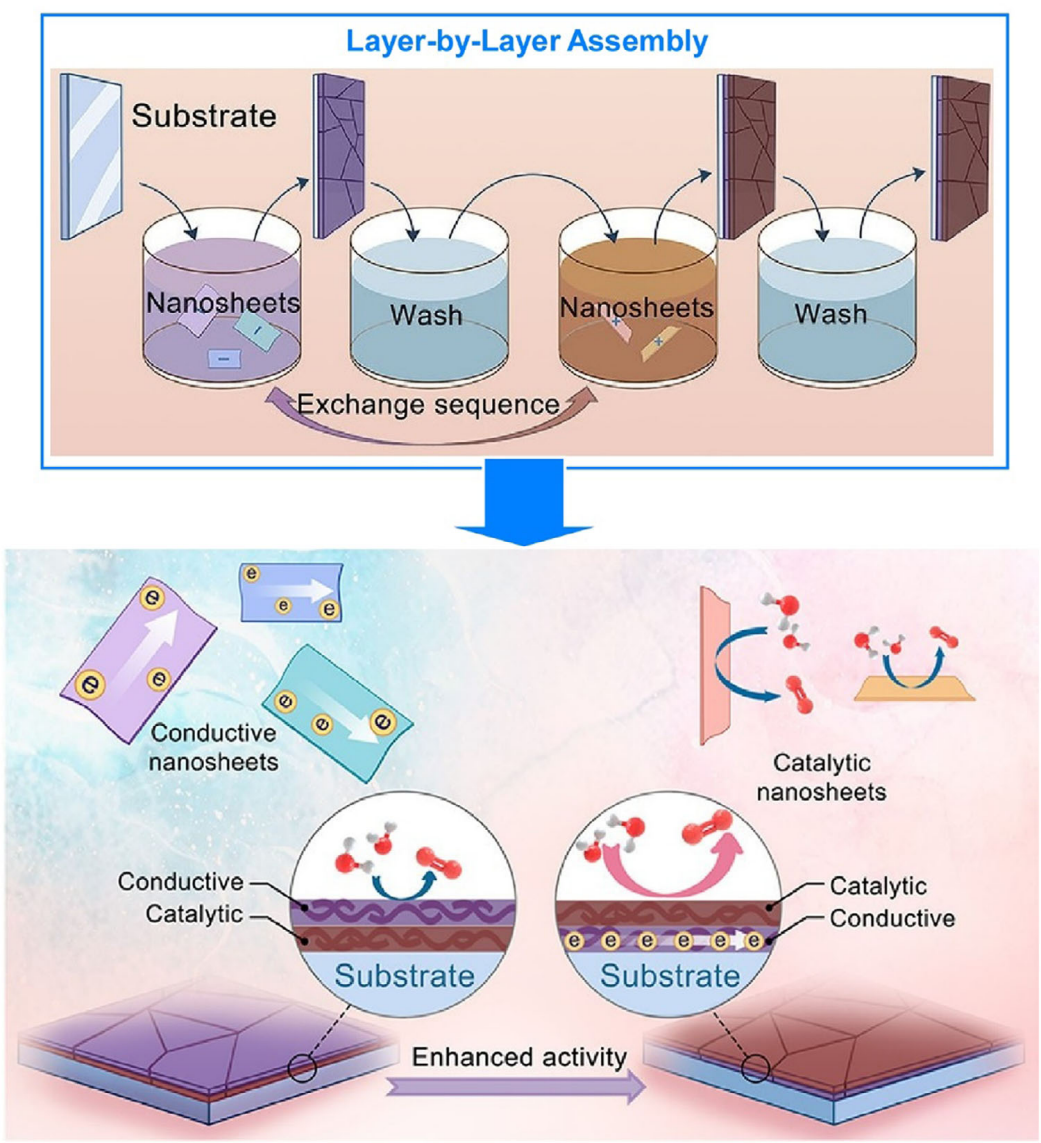
LbL assembly of oxide nanosheets, reduced graphene oxide, and layered double hydroxide nanosheets to investigate the correlation between nanosheet sequence and function in the oxygen evolution reaction (OER), where the highest OER activity was achieved in a bilayer film of conductive nanosheets under catalytically active layered double hydroxide (LDH) nanosheets. Reprinted with permission from Ref. [66] Copyright 2022 American Chemical Society.

The assembly morphology of nanosheets, as mentioned above, is important, as is nanoarchitectonics regarding the organized structure of multiple types of nanosheets. In particular, the aggregation or restacking of different types of 2D nanosheets to form heterostructured nanocomposites is important in the development of high‐performance electrode materials and catalysts. Liu, Ma, and their colleagues performed LbL assembly using oxide nanosheets, reduced graphene oxide, and LDH nanosheets, investigating the correlation between nanosheet sequence and function in the oxygen evolution reaction (OER) (Figure 15).^[^ ^66^ ^]^ The highest OER activity was achieved in a bilayer film of conductive RuO2.1 nanosheets under catalytically active NiFe LDH nanosheets (NiFe LDH_(Td/Oh)) with mixed octahedral/tetrahedral coordination. DFT calculations and simulations revealed that the improved catalytic performance is due to the large electronic coupling effect in the heterostructure. The exposed surface layer serves as the main active center. In the heterostructure, this enhanced catalytic performance was shown to result from significant interfacial electronic coupling, whereby electrons transfer from the exposed layer to the underlying layer. Rational nanosheet arrangement strategies at the nanoscale are crucial for developing 2D heterostructured nanocomposites for highly efficient water oxidation and other heterogeneous catalysis processes.

**Figure 16:**
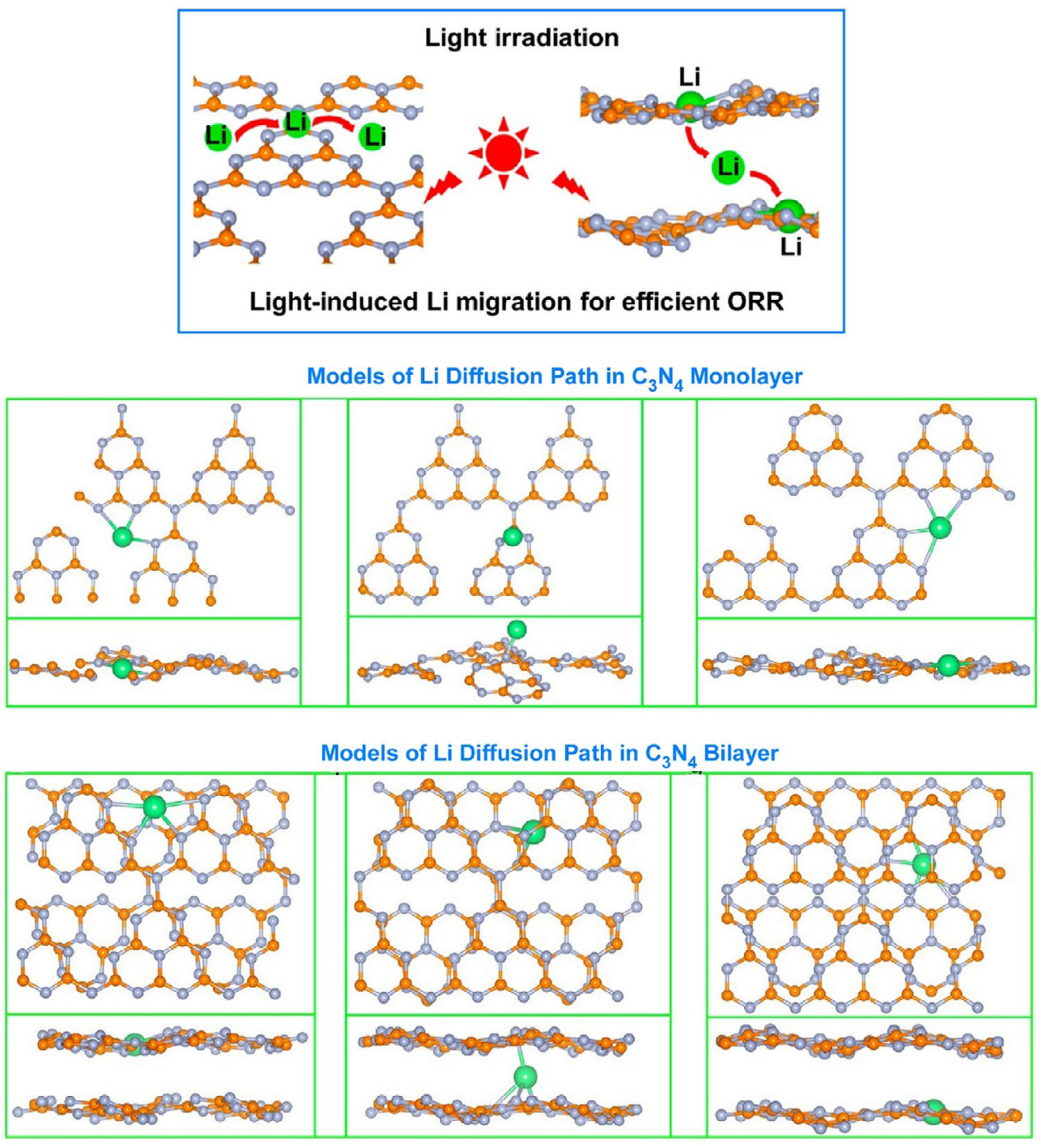
Movements of the lithium metal atoms upon light irradiation within the single‐layer C_3_N_4_ and diffused between the multi‐layer C_3_N_4_ (top) with DFT calculations for Li diffusion path (bottom). Reprinted with permission from Ref. [67] Copyright 2024 American Chemical Society.

Dynamic structural changes in nanosheet assembly structures can affect their functionality. The structure and electronic state of the active center of single‐atom catalysts change significantly during dynamic catalytic processes. The active centers of metal atoms have not yet been fully demonstrated using dynamic methods. Zhuang et al. created extremely light lithium metal atoms on C_3_N_4_ nanosheets to serve as dynamic active centers and achieve highly efficient photocatalytic reactions.^[^ ^67^ ^]^ Upon light irradiation, the lithium metal atoms moved within the single‐layer C_3_N_4_ and diffused between the multi‐layer C_3_N_4_. This resulted in a significant change to the local coordination environment of the lithium (Figure 16). This movement altered the local coordination environment of Li in the C_3_N_4_ nanosheets, significantly enhancing the photocatalytic activity. DFT calculations showed that this dynamic Li coordination structure contributes to the ultrahigh photocatalytic activity. To determine the most favorable Li‐coordinated C_3_N_4_ structure for H_2_O_2_ evolution, reaction paths were calculated for various structures. The Li‐coordinated C_3_N_4_ structure was found to outperform the pure C_3_N_4_ structure in terms of H_2_O_2_ evolution. Additionally, the Li‐coordinated bilayer C_3_N_4_ structure was more effective than the Li‐coordinated monolayer C_3_N_4_ structure, and the Li‐coordinated initial and final C_3_N_4_ structures were more effective than the transition structures. These findings offer a fresh perspective on the pivotal role of dynamic active centers in catalytic reactions. This is a good example of the dynamic function of nanoarchitectonics nanosheet structures.

**Figure 17:**
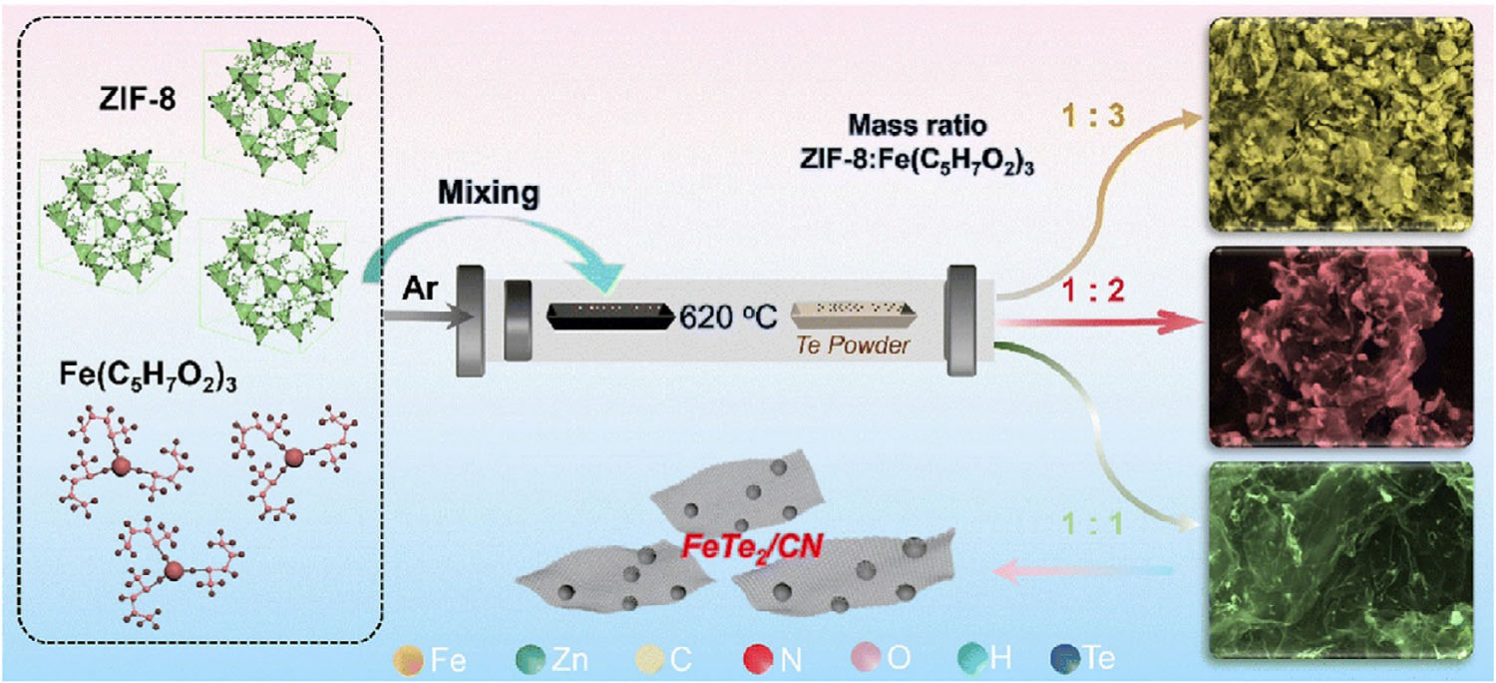
A scalable preparation method of porous, ultrathin, nitrogen‐doped carbon nanosheets decorated with ultrafine FeTe_2_ nanoparticles using a mild, modifier‐free synthesis strategy from metal‐organic frameworks (MOFs) as the raw material, where nitrogen‐doped carbon nanosheets were synthesized at 620 °C using metal telluride nanoparticles grown in situ with high‐purity tellurium powder, with ZIF‐8 acting as the carbon source. Reprinted with permission from Ref. [68] Copyright 2025 Royal Society of Chemistry.

The nanoarchitectonics approach, which enables the control of nanosheet pore structure and surface properties, is also important. Carbon materials prepared using precursors such as MOFs, rather than conventional 2D materials, are attracting attention too. 2D nanocarbon‐based materials with a controllable pore structure and a hydrophilic surface have great potential for various applications. However, it is difficult for conventional 2D carbon nanomaterials to achieve this goal. Yang, Zhou, Chen, and colleagues have demonstrated a scalable method of preparing porous, ultrathin, nitrogen‐doped carbon nanosheets decorated with ultrafine FeTe_2_ nanoparticles using a mild, modifier‐free synthesis strategy from MOFs as the raw material (Figure 17).^[^ ^68^ ^]^ Nitrogen‐doped carbon nanosheets (FeTe_2_/CN) were synthesized at 620 °C using metal telluride nanoparticles grown in situ with high‐purity tellurium powder, with ZIF‐8 acting as the carbon source. Lithium polysulfides are efficiently captured via synergistic adsorption through the lithium affinity sites of nitrogen‐doped carbon and the sulfur affinity sites of FeTe_2_. The combination of the enhanced electrical conductivity of carbon nanotube nanosheets and the strong spin‐state effect of FeTe_2_ facilitates electron transfer, lowers the energy barrier, and improves the rate of sulfur redox reactions. Like graphene, nitrogen‐doped carbon nanosheets can provide a large number of active sites and mitigate volume expansion during cycling. Thanks to these properties, lithium–sulfur batteries based on the cathodes demonstrate high initial capacity, exceptional rate capability, and excellent stability. The material is extremely lightweight and flexible, making it promising for use in flexible batteries. This work may provide valuable insights into designing transition metal chalcogenide 2D material hosts with exceptional activity.

### 3.2 Recent Progress of LbL Assembly

In the previous section, we focused on nanosheets as components of layered structures. LbL assembly is often used for this purpose, and it has been reported that different types of nanosheets can be used to nanoarchitect materials with the highest performance in a rational way. In addition to being a simple method, LbL assembly is applicable to a wide variety of materials and can introduce various interactions.^[^ ^69^ ^]^ It is an excellent method for organizing layered materials composed of various components, not limited to nanosheets. Although LbL assembly predates nanoarchitectonics, the method is still evolving. Below, we provide an overview of such trends.

**Figure 18:**
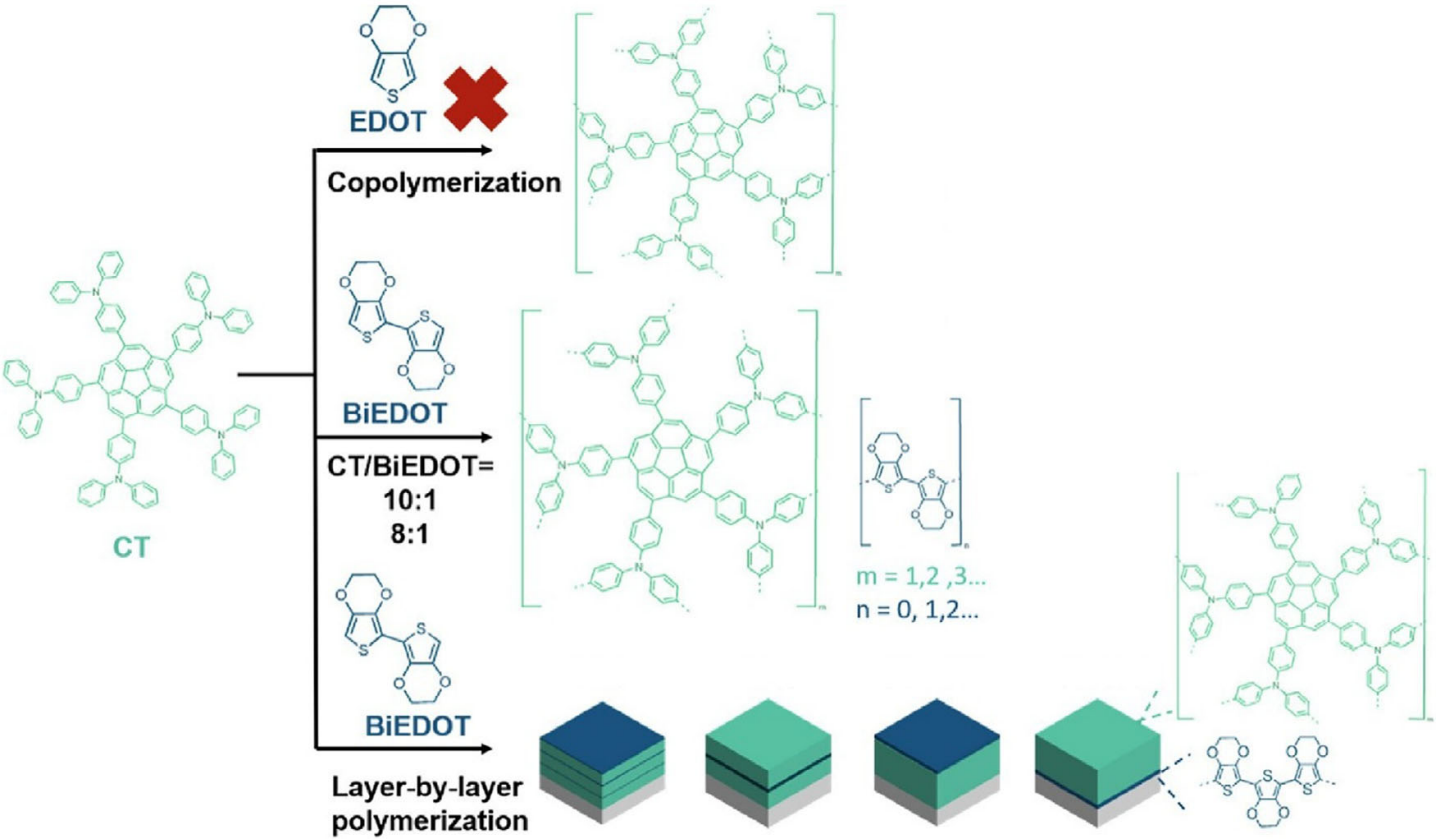
Electrochemical and electrochromic properties of polymers synthesized by electrochemical copolymerization and layer‐by‐layer polymerizations of corannulene‐(triphenylamine)_5_ and 3,4‐ethylenedioxythiophene, in the latter of which a variety of layer structures are possible. Reproduced under terms of the CC‐BY license from Ref. [70], 2025 American Chemical Society.

LbL assembly is a highly flexible methodology that can incorporate a wide range of other related techniques. For instance, some LbL assembly approaches incorporate polymerization reactions. Yu and Luo compared the electrochemical and electrochromic properties of polymers synthesized by electrochemical copolymerization and LbL polymerizations of corannulene‐(triphenylamine)_5_ and 3,4‐ethylenedioxythiophene (Figure 18).^[^ ^70^ ^]^ In electrochemical copolymerization, the oxidation potential of 3,4‐ethylenedioxythiophene is higher than that of corannulene‐(triphenylamine)_5_, which makes copolymerization impossible. Consequently, a dimer of 3,4‐ethylenedioxythiophene with an equivalent oxidation potential to corannulene‐(triphenylamine)_5_ was synthesized to facilitate copolymerization. In the LbL polymerization method, however, the concentration of each monomer in the electrolyte, the number of CV cycles of each monomer, i.e., the number of layers and their order, must be carefully considered. A variety of layer structures is possible. Layered nanoarchitectonics have great potential to impart new electrochromic properties to the resulting polymer. LbL polymerization produces films that exhibit different electrochemical behavior from conventional copolymer films. Furthermore, these properties can be altered by adjusting the number of cycles and the deposition sequence. Moreover, LbL films exhibit higher near‐infrared absorption and superior optical properties compared to conversional copolymer films. The LbL polymerization method has demonstrated the feasibility of this innovative approach to constructing electrochromic films.

**Figure 19:**
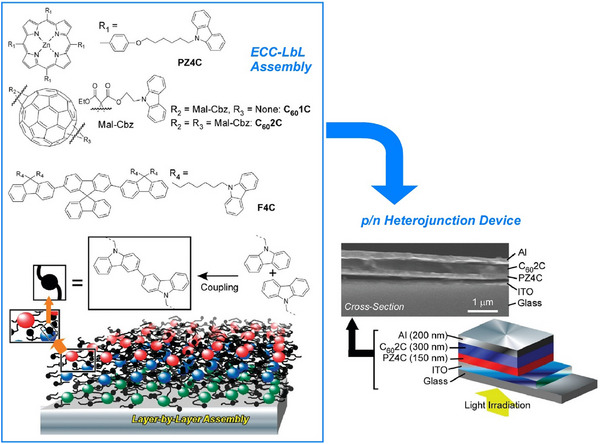
Electrochemical‐coupling layer‐by‐layer (ECC‐LbL) assembly in both homo‐ and hetero‐assemblies with the desired thickness and sequence: assembling principle (left) and application to a prototype p/n heterojunction device (right). Reprinted with permission from Ref. [71a] Copyright 2011 American Chemical Society.

Methods of nanoarchitectonics have also been developed that utilize electrochemical coupling reactions to freely construct functional layers. Li et al. have proposed a method called electrochemical‐coupling layer‐by‐layer (ECC‐LbL) assembly (Figure 19).^[^ ^71^ ^]^ ECC‐LbL assembly enables the layering of functional units such as porphyrins, fullerenes, and fluorenes in both homo‐ and hetero‐assemblies with the desired thickness and sequence. Dimerization of *N*‐alkylcarbazoles was selected as a coupling reaction that can be induced by electrochemical stimulation from the electrode surface. *N*‐alkylcarbazoles and their dimers exhibit high hole transport mobility, enabling electrochemical signals to be transmitted to the top layer of the film. Molecular thin films can be assembled stepwise through this electrochemical coupling reaction. This simple and inexpensive process can produce various layer arrangements using conventional electrochemical equipment, and nano‐thin film structures can be constructed simply by manipulating the external potential. Functional units can be covalently immobilized on thin films in a designable arrangement to ensure efficient interlayer electronic interactions. For example, one attractive potential application of heterolayer films is in thin‐film optoelectronic devices, the usefulness of which has been demonstrated. A prototype p/n heterojunction device was fabricated using the ECC‐LbL assembly method, with donor and acceptor layers assembled between electrodes. Its photovoltaic properties were then investigated. The device exhibited a photovoltaic effect under white light illumination. Further improvement of the device performance could be achieved through appropriate modification of the film structure and composition. As the coupling reaction sites are independent of functional groups, this method can be used in molecules containing many different types of organic functional groups. Consequently, the ECC‐LbL approach is anticipated to be applicable to a variety of organic devices. In addition to the ECC‐LbL approach, the other approach that enables similar features was reported as an electro‐click approach for LbL assemblies.^[^ ^32e^ ^]^ This electro‐click assembly is effective for the fabrication of a synapse‐like device. At the same time, there are more possibilities of electro‐click assembly in LbL films and capsules.

**Figure 20:**
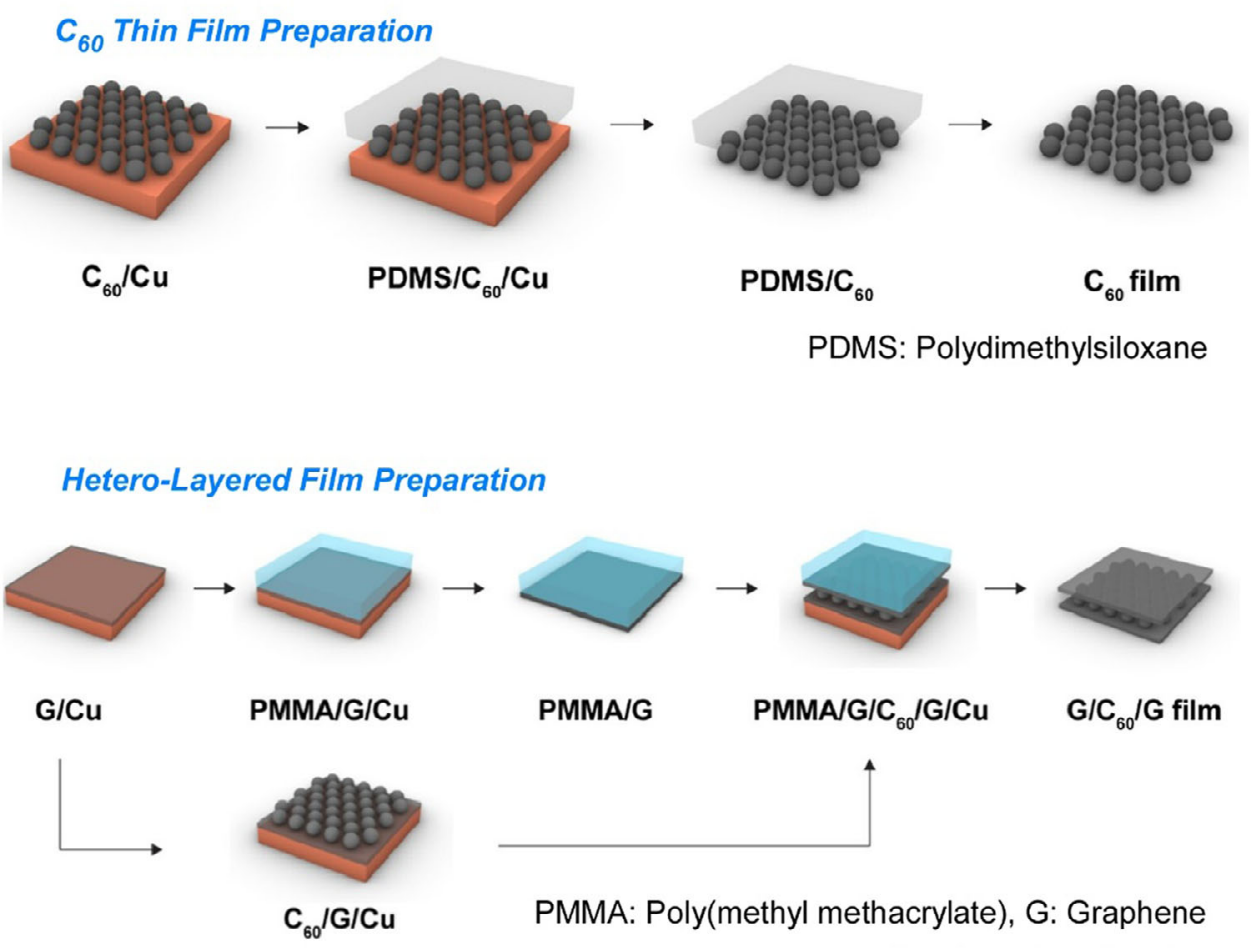
Large‐area transfer of nanometer‐thin C_60_ films: the solid‐phase transfer of nanometer‐thin C_60_ films with centimeter‐scale lateral sizes and controlled thicknesses ranging from 1 to 20 nm onto various substrates (top) and centimeter‐wide graphene/C60/graphene heterostructures fabricated by layering C_60_ and graphene films in the layer‐by‐layer fashion (bottom). Reprinted with permission from Ref. [72] Copyright 2025 American Chemical Society.

Other operations, such as transfer processes, have also been incorporated to fabricate layered structures. Chen and colleagues reported a study titled “Large‐area transfer of nanometer‐thin C_60_ films,” which incorporated a film transfer process (Figure 20).^[^ ^72^ ^]^ This method involves the solid‐phase transfer of nanometer‐thin C_60_ films with centimeter‐scale lateral sizes and controlled thicknesses ranging from 1 to 20 nm onto various substrates. Centimeter‐wide graphene/C_60_/graphene heterostructures can be fabricated by layering C_60_ and graphene films in the layer‐by‐layer fashion. A Cu foil was used as the substrate, and C_60_ layers were thermally evaporated onto graphene films grown on Cu foils using chemical vapor deposition. The stamping method was then employed to transfer the as‐deposited C_60_ films. This transfer mechanism is based on the difference in adhesion between the C_60_ and the substrate, and the C_60_ and the stamp. This versatile transfer method can also be used to transfer chemically modified C_60_ films, such as oxygenated C_60_ films and C_60_Pd_n_ organometallic polymer films. Direct solid‐phase transfer of C_60_ and C_60_Pd_n_ films onto electrode surfaces allows their electrocatalytic performance in the hydrogen evolution reaction to be evaluated directly. Indeed, transferring large‐area C_60_ thin films allows for the production of artificial materials made from multidimensional carbon allotropes. Additionally, thin films or heterostructures can be prepared and transferred from other molecular building blocks, including different fullerene types such as C_70_ and various endohedral fullerenes. With their well‐defined molecular structures and high scalability, fullerenes are promising fundamental building blocks for creating a variety of carbon materials. Fabricating and transferring large‐area films with precisely controlled thickness and morphology onto desired surfaces is highly promising for designing and developing fullerene‐based materials and devices.

**Figure 21:**
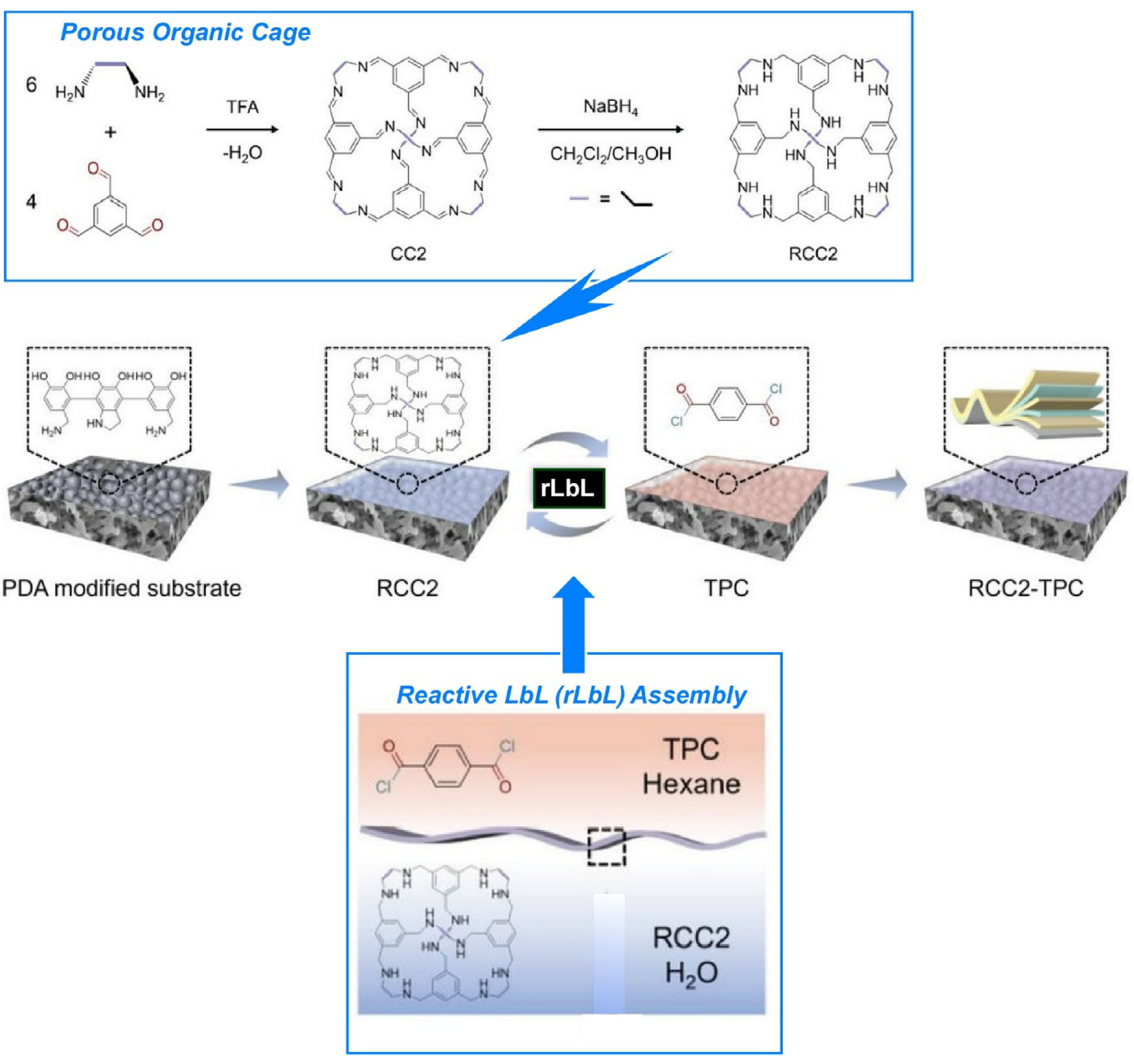
Fabrication of an ion‐sieving membrane through reactive Layer‐by‐Layer (rLbL) assembly (bottom) of porous organic cage (top). Reprinted with permission from Ref. [73] Copyright 2024 Wiley‐VCH.

Introducing reactions into the LbL process can result in the production of more stable and practical thin films. For instance, membranes with sub‐nanometer‐sized pores, produced using this approach, could be used in the treatment of saline wastewater and the recovery of resources. Zhao and colleagues fabricated a novel ion‐sieving membrane through the reactive layering of reduced porous organic cages (Figure 21).^[^ ^73^ ^]^ First, the surface of a polysulfone substrate was modified via a self‐polymerization reaction involving dopamine. This polydopamine layer provides a hydrophilic, uniform surface, which is favorable for the formation of defect‐free selective layers. The reduced [4 + 6] imine cages were then stacked in a thin layer by reactive LbL assembly to fabricate the ion‐sieving membrane. During this process, the secondary amine groups react with the acyl chloride groups of terephthaloyl chloride at the interface, forming a continuous, colorless membrane. The resulting ion‐sieving membranes' physical and chemical properties can be controlled by adjusting the concentration of the reduced [4 + 6] imine cages. With excellent Li^+^/Mg^2+^ selectivity, lithium extraction from simulated salt lake brine was achieved using a two‐stage nanofiltration process that reduced the Mg^2+^/Li^+^ mass ratio from 40 to 0.3. This further demonstrates the potential for application in lithium resource recovery.

**Figure 22:**
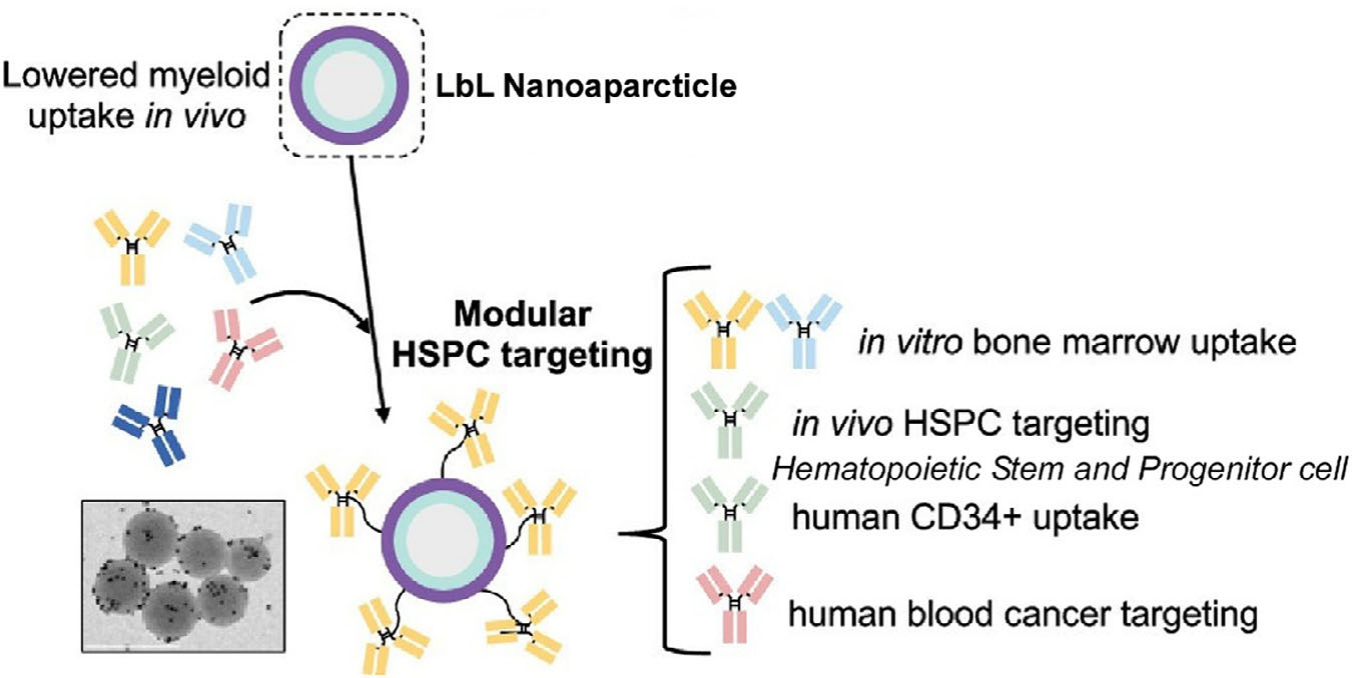
Preparation of LbL nanoparticles that target hematopoietic stem and progenitor cells (HSPCs) through antibody functionalization, reducing off‐target uptake by circulating bone marrow cells. Reprinted with permission from Ref. [75] Copyright 2025 American Chemical Society.

LbL assembly is not limited to creating planar multilayer structures. It is also widely recognized as a useful method for creating modified nanoparticles and microcapsule membranes by layering the components onto nano‐ and microparticles.^[^ ^74^ ^]^ Taking advantage of the characteristics of the LbL method, modified nanoparticles and microcapsules with various functions can be fabricated for medical use. Efficient delivery of drugs and genes to hematopoietic stem and progenitor cells is a major challenge. LbL nanoparticles can be highly targeted by modifying their surface properties. Hammond and colleagues developed LbL nanoparticles that target hematopoietic stem and progenitor cells through antibody functionalization, reducing off‐target uptake by circulating bone marrow cells (Figure 22).^[^ ^75^ ^]^ First, they investigated surface polymers that could minimize the undesirable interaction between circulating bone marrow cells and LbL nanoparticles. Polyacrylic acid, a bioinert polymer, was found to exhibit excellent stealth properties in vivo. The surface was then modified using antibodies that target hematopoietic stem and progenitor cells, and promising candidate antibodies were systematically compared. In vitro studies with mouse bone marrow cells demonstrated the effect of LbL nanoparticle uptake and antibody trafficking patterns. Antibodies that caused maximum binding were then identified. To evaluate the potential application of these targets, the enhanced binding of LbL nanoparticles was confirmed in vitro using human CD34 + cells. Designs were also explored to enhance LbL nanoparticle binding in human leukaemia and lymphoma cell lines. Overall, these results suggest that this modular LbL nanoparticle platform, which can target hematopoietic stem and progenitor cells in a disease‐dependent manner, has therapeutic application potential. This system may be useful for designing systematic studies to develop optimized drug delivery systems with therapeutic potential.

In this section, we demonstrate nanoarchitectonics using 2D materials (nanosheets) and material organization by LbL assembly, a powerful method of layered nanoarchitectonics. By limiting the direction of organization to a certain extent, such as a layered structure, the research appears to be more focused, with many developments having been made. Even when limited to a layered structure, diverse components can be used to create a wide variety of functions. In particular, layered structures have many unique functional designs, such as optimization by layering order and functions that reflect the high aspect ratio of 2D materials, such as anisotropy. Due to increasing interest in 2D materials and the high versatility of LbL assembly, many functional material systems have been developed. These systems allow fine adjustment of functions and are advantageous for developing practical materials. A major feature of layered nanoarchitectonics is that it has various potential applications. Further developments with an eye towards practical use are expected.

## 4 Nanoarchitectonics at Liquid Interface

In the development of functional materials through nanoarchitectonics, a wide variety of materials can be constructed thanks to the freedom to select components and the range of assembly methods available. Some assembly processes are dynamic and flexible, and attempts are being made to fine‐tune the assembly process. The highly dynamic properties of the environment in which nanoarchitectonics is carried out can maximize the advantages of such dynamic natures. One such environment is a liquid interface. In this section, we focus on nanoarchitectonics at liquid interfaces.^[^ ^76^ ^]^ Specifically, we will discuss two very different systems: the assembly of various molecules and materials, and the assembly of living cells.

### 4.1 Liquid Interfacial Molecular/Materials Nanoarchitectonics

**Figure 23:**
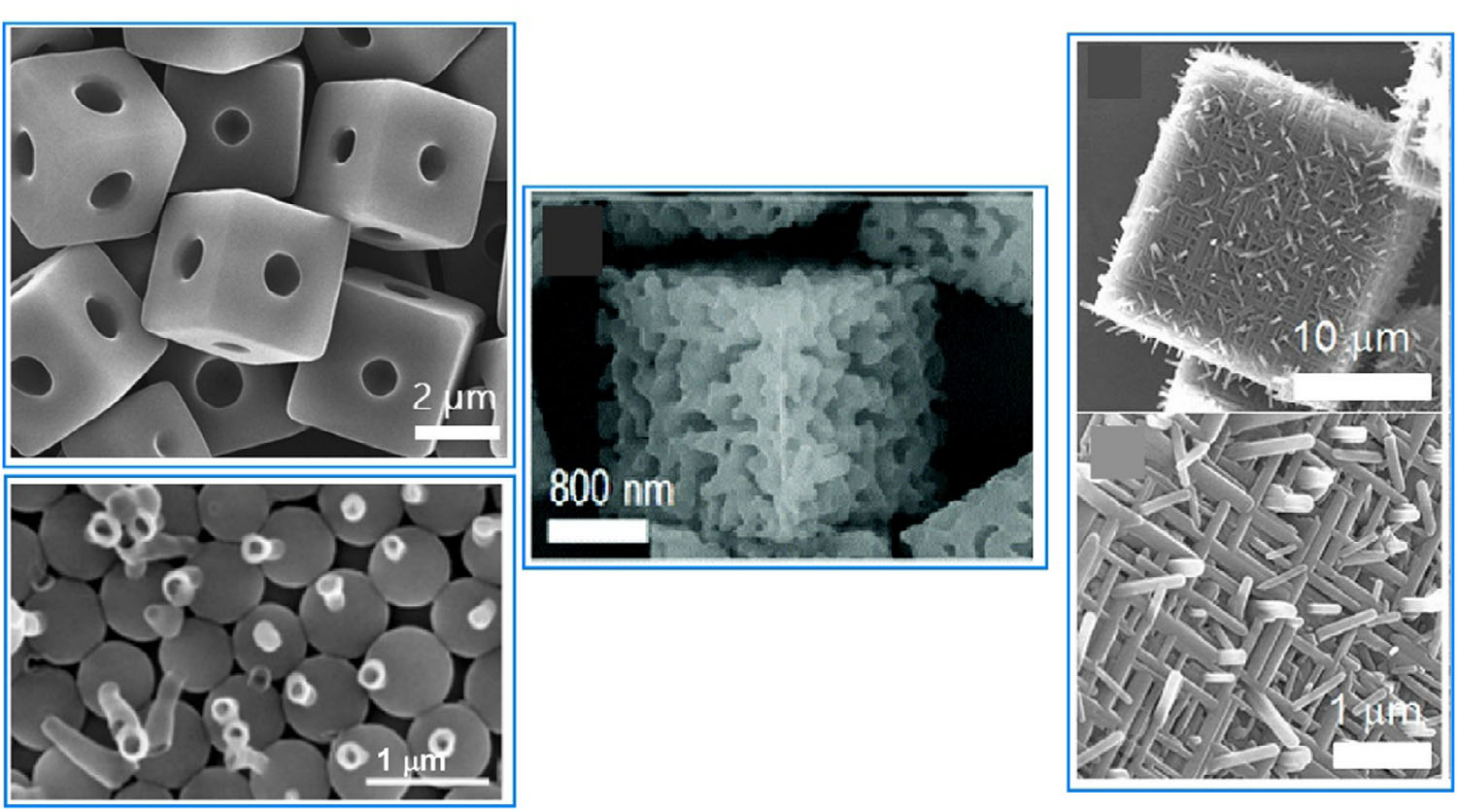
A variety of hierarchical structures through interfacial assembly of fullerene molecules. Reprinted with permission from Ref. 81d. Copyright 2017 American Chemical Society, from Ref. 81c. Copyright 2016 American Chemical Society, from Ref. 81e. Copyright 2020 Royal Society of Chemistry, and from Ref. [81b] Copyright 2016 American Chemical Society.

The first example of nanoarchitectonics at liquid interfaces involves fullerenes as components. Using fullerenes in nanoarchitectonics demonstrates the potential for generating various structures from simple components. The potential applications of this technology are vast. These approaches used fullerenes (C_60_, C_70_, etc.), which are the simplest in shape and are made of a single element, as the original molecules to be assembled. To assemble and organize molecular units into interesting shapes, it is important to create delicate conditions in which the molecules are on the verge of gathering. One method is liquid–liquid interfacial precipitation, whereby fullerene molecules are dissolved in a solvent with high fullerene solubility and then a second immiscible solvent with low fullerene solubility is added.^[^ ^77^ ^]^ The interface between these liquids is in a delicate state, where the fullerene molecules are likely to assemble. From there, fullerene aggregates of various shapes can form. There are an infinite number of solvent combinations, so experiments can be tested with a variety of solvent combinations as desired. Self‐assembled structures such as rod‐like structures,^[^ ^78^ ^]^ tubes,^[^ ^79^ ^]^ and hexagonal or rhombic nanosheets^[^ ^80^ ^]^ can be obtained. Furthermore, various processes can be employed to nanoarchitectonically produce a variety of hierarchical structures,^[^ ^81^ ^]^ as shown in Figure 23.

**Figure 24:**
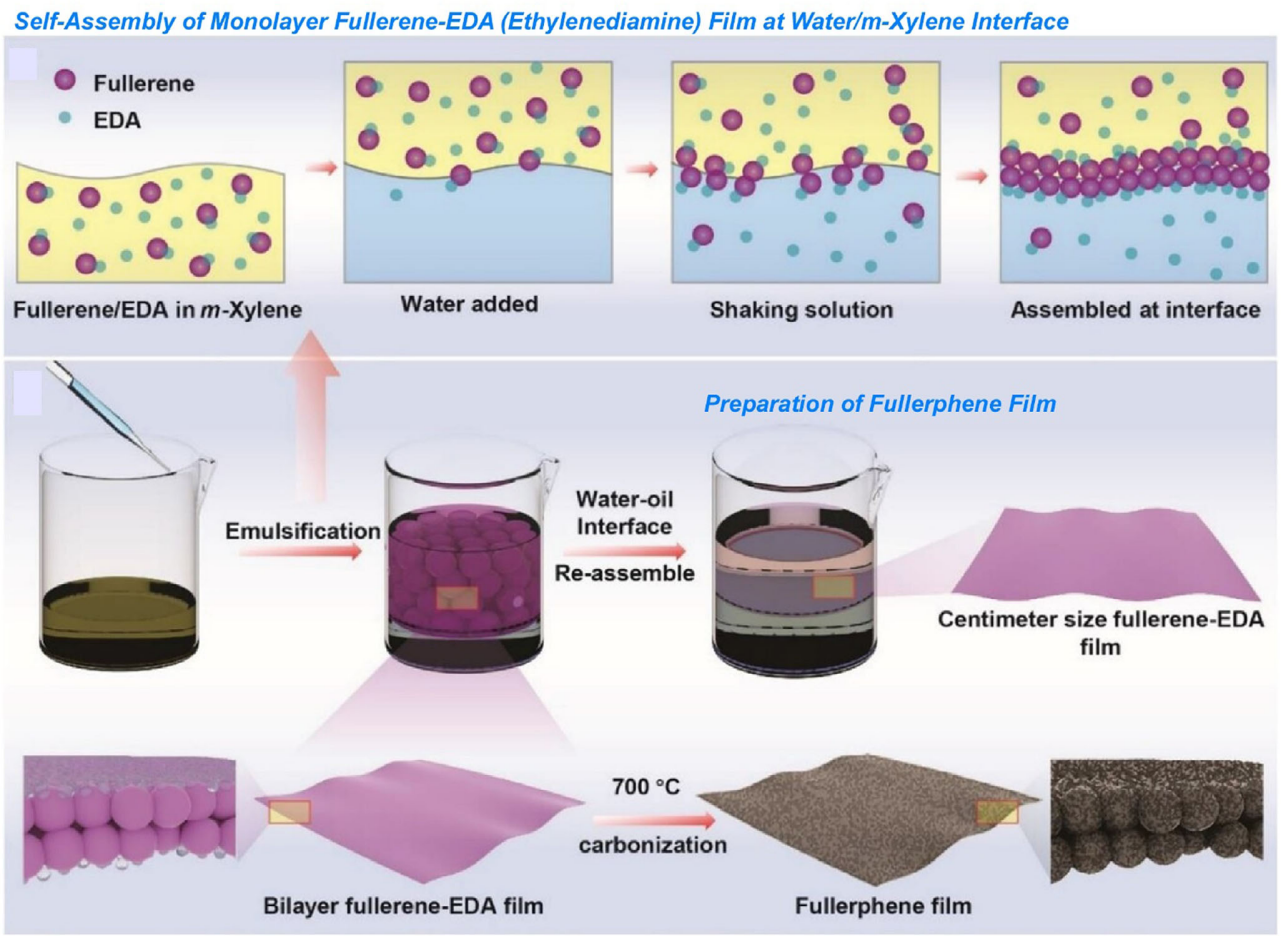
Bottom‐up fabrication of a novel nitrogen‐doped 2D carbon material, fullerphene, by interfacial nanoarchitectonics of C_60_ fullerene. Reprinted with permission from Ref. [83] Copyright 2022 Wiley‐VCH.

Also, 2D carbon materials can be created from fullerenes using a process called liquid interface nanoarchitectonics. Ultrathin 2D nanoporous carbon materials, in particular, are important for the selective recognition of guest molecules as they provide superior sensitivity and high spatial resolution in sensing applications. Song et al. reported the bottom‐up fabrication of a novel nitrogen‐doped 2D carbon material, fullerphene, by interfacial nanoarchitectonics of C_60_ fullerene (Figure 24).^[^ ^82^ ^]^ This provides a simple yet versatile method of forming macroscopically extended carbon ultrathin films (≈ 2 nm). First, a large‐area, molecular‐level‐thick 2D fullerene‐ethylenediamine film was fabricated by the self‐assembly and crosslinking of fullerene and ethylenediamine (EDA) molecules at the water/m‐xylene liquid–liquid interface. This was then carbonized at 700 °C to produce a carbon nanofilm with a thickness at the nanometer level. This carbon thin film is called fullerphene because it is a graphene‐like film made from fullerenes using a bottom‐up approach. Although fullerphene is a 2D carbon film, it is nitrogen‐doped and has a hierarchical micro/mesoporous structure on the surface. It has an ultrafine porous nanostructure consisting of sp^2^‐bonded carbon atoms doped with pyrrole‐type and quaternary nitrogen‐based atoms. Therefore, it exhibits superior sensitivity to formic acid vapor compared to other common low‐molecular‐weight carboxylic acid molecules. Its sensitivity to formic acid is much higher than that to acetic acid, demonstrating that 2D fullerphene provides an attractive platform for identifying carboxylic acids at the single‐carbon‐atom level.

In addition to fullerenes, various functional molecules and materials can be organized at liquid interfaces through nanoarchitectonics. Studies have also analyzed the details of such processes. Conductive polymer thin films have a wide range of applications, including in bioelectronics, energy harvesting and storage, and drug delivery technology. Electrosynthesis at a polarizable liquid/liquid interface using aqueous oxidants and organic‐soluble monomers provides a single‐step method for fabricating free‐standing, scalable conductive polymer thin films of poly(3,4‐ethylenedioxythiophene) at room temperature. Gamero‐Quijano, Scanlon, and colleagues used potentiodynamic cyclic voltammetry to investigate this process in detail.^[^ ^83^ ^]^ They demonstrated that interfacial electrosynthesis involving ion exchange, electron transfer, and proton adsorption charge transfer processes is completely different at the liquid–liquid interface than at the conventional solid electrode/electrolyte interface. Electrochemical behaviors typical of conducting polymer electropolymerization, such as stable charge accumulation and the appearance of nucleation loops at each cycle, were observed. This study demonstrates the importance of thoroughly understanding the mechanistic origins using the classical potentiodynamic technique of cyclic voltammetry. This allows the electrochemical monitoring of the effects of experimental variables, as well as the correlation of changes in physical properties such as morphology, crystallinity, electrical conductivity, and catalytic activity in the resulting conducting polymer thin films.

**Figure 25:**
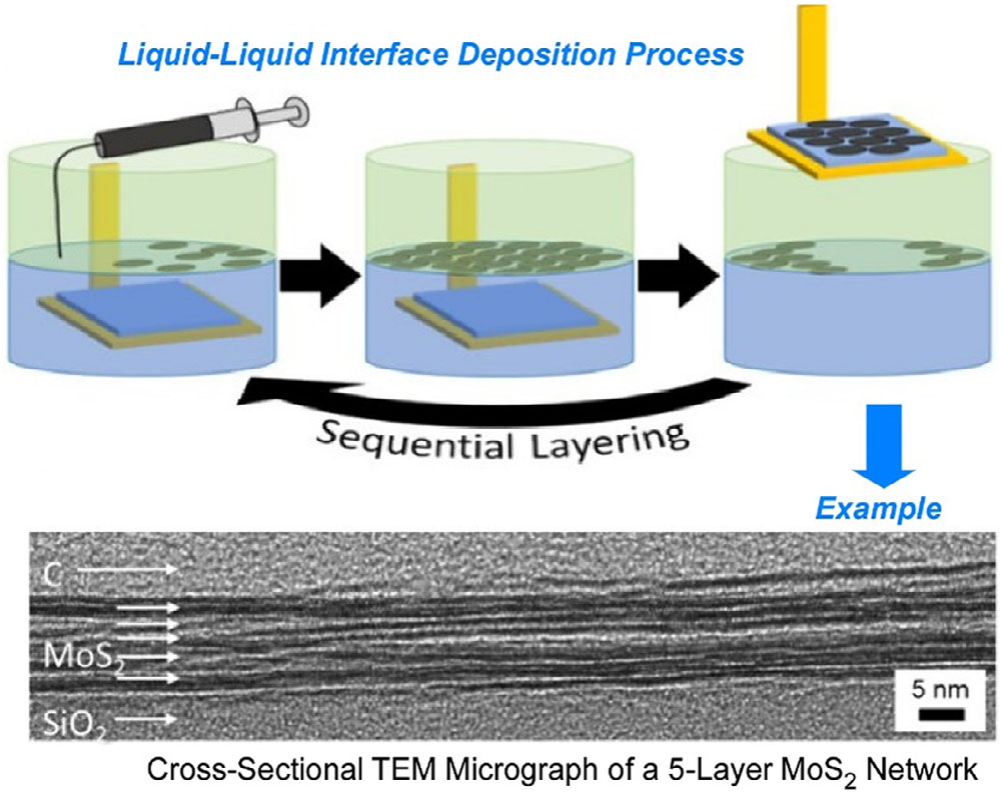
A method to maximize nanosheet alignment and network uniformity, and reduce junction resistance, by utilizing advanced liquid interface deposition processes to maximize nanosheet alignment and network uniformity, and reduce junction resistance. Reproduced under terms of the CC‐BY license from Ref. [84], 2024 American Chemical Society.

Functional materials, such as nanosheets, are also of interest in the context of liquid interface nanoarchitectonics. From a practical perspective, solution‐processable 2D materials show great promise for various printed electronics applications. A key step in implementing 2D materials in printed electronics applications is achieving junction/nanosheet resistance of less than 1. To meet this demand, Coleman et al. demonstrated a method to maximize nanosheet alignment and network uniformity, and reduce junction resistance, by utilizing advanced liquid interface deposition processes (Figure 25).^[^ ^84^ ^]^ They used this approach with 2D materials such as graphene and MoS_2_ as model materials. It becomes a method for depositing large‐area, densely tiled nanosheet networks through assembly at immiscible liquid–liquid interfaces, such as the water‐hexane interface. Nanosheets trapped at the high‐energy water‐hexane interface are thermodynamically stable, as the tendency for them to overlap is minimized, and they are tightly confined at the interface. An isopropyl alcohol ink is then injected at the interface, which lowers the interfacial tension at that location. This creates a tension gradient that causes the nanosheets to come together and form a monolayer with primarily edge‐to‐edge contacts. Finally, the interface‐assembled monolayer is stabilized by capillary forces between the nanosheets as it is deposited onto the substrate. A single deposition process results in a highly aligned monolayer comprising edge‐connected individual nanosheets. Subsequent depositions enable the formation of highly organized multilayer structures. This process contrasts with spray‐coating and spin‐coating, where random coverage is usually described by a Poisson distribution. This deposition process can also be applied to non‐layered, quasi‐2D materials, such as silver nanosheets. These materials exhibit electrical conductivity close to that of bulk silver for networks less than 100 nm thick. The electrical performance of these networks is dependent on thickness and can be systematically measured, achieving junction resistance/nanosheet resistance of less than 1. This technique could be useful for printed electronics based on 2D materials.

**Figure 26:**
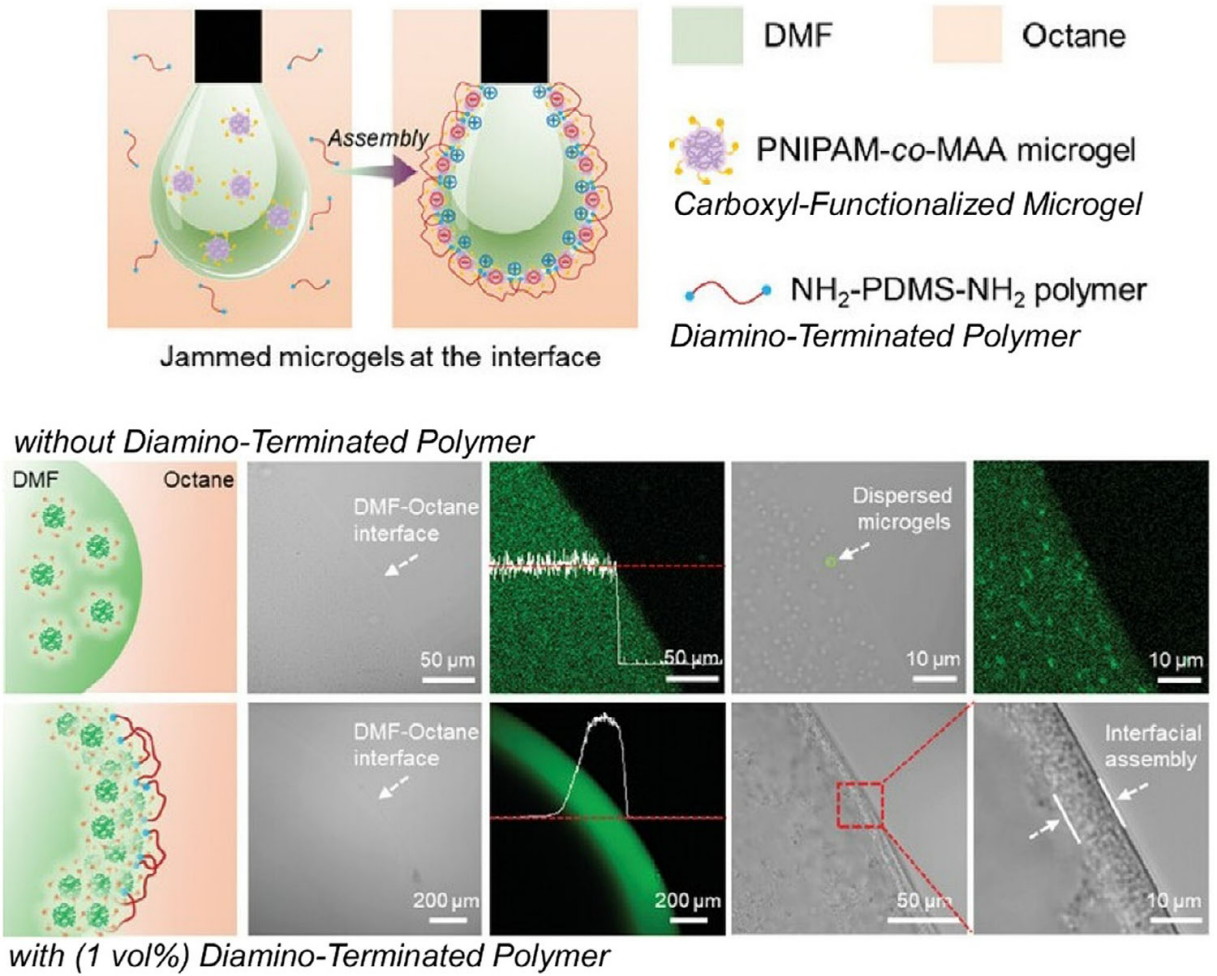
Formation of stabilized non‐aqueous Pickering emulsions via the self‐assembly of complementary, carboxyl‐functionalized microgels and diamino‐terminated, telechelic polymer ligands (top) and assembly and jamming of microgel–polymer complexes at non‐aqueous liquid–liquid interfaces (bottom). Reproduced under terms of the CC‐BY license from Ref. [85], 2025 Wiley‐VCH.

The type of liquid interface also significantly impacts the fabrication of structures. Compared with the commonly used oil–water system, a substantial decrease in interfacial tension occurs at the oil–oil interface, hindering the efficient adsorption and assembly of particles. Kwok, Jiang, Ngai, and colleagues demonstrated how microgels and polymer ligands can be assembled and anchored at non‐aqueous liquid–liquid interfaces to fabricate non‐aqueous Pickering emulsions and reconfigurable droplet networks (Figure 26).^[^ ^85^ ^]^ They stabilized non‐aqueous Pickering emulsions via the self‐assembly of complementary, carboxyl‐functionalized microgels and diamino‐terminated, telechelic polymer ligands. They also demonstrated the assembly and jamming of microgel‐polymer complexes at non‐aqueous liquid–liquid interfaces. After forming monolayer assemblies at the DMF‐octane interface, the microgels migrated from the bulk DMF phase to the interface, eventually forming a multilayer structure with a thickness of about 10 µm. An asymmetric Janus bilayer formed at the oil–oil interface from the microgel‐polymer complexes. Maintaining sufficient strength, flexibility, and semipermeability enabled the reconfigurable droplet network to selectively adsorb and permeate specific molecules. Using the non‐aqueous Pickering emulsion as a template, heterogeneous organogels and microgel‐based colloidosomes can be fabricated using covalent or non‐covalent cross‐linking methods. This approach opens up new avenues for designing and fabricating stimuli‐responsive non‐aqueous microreactors and organogels.

**Figure 27:**
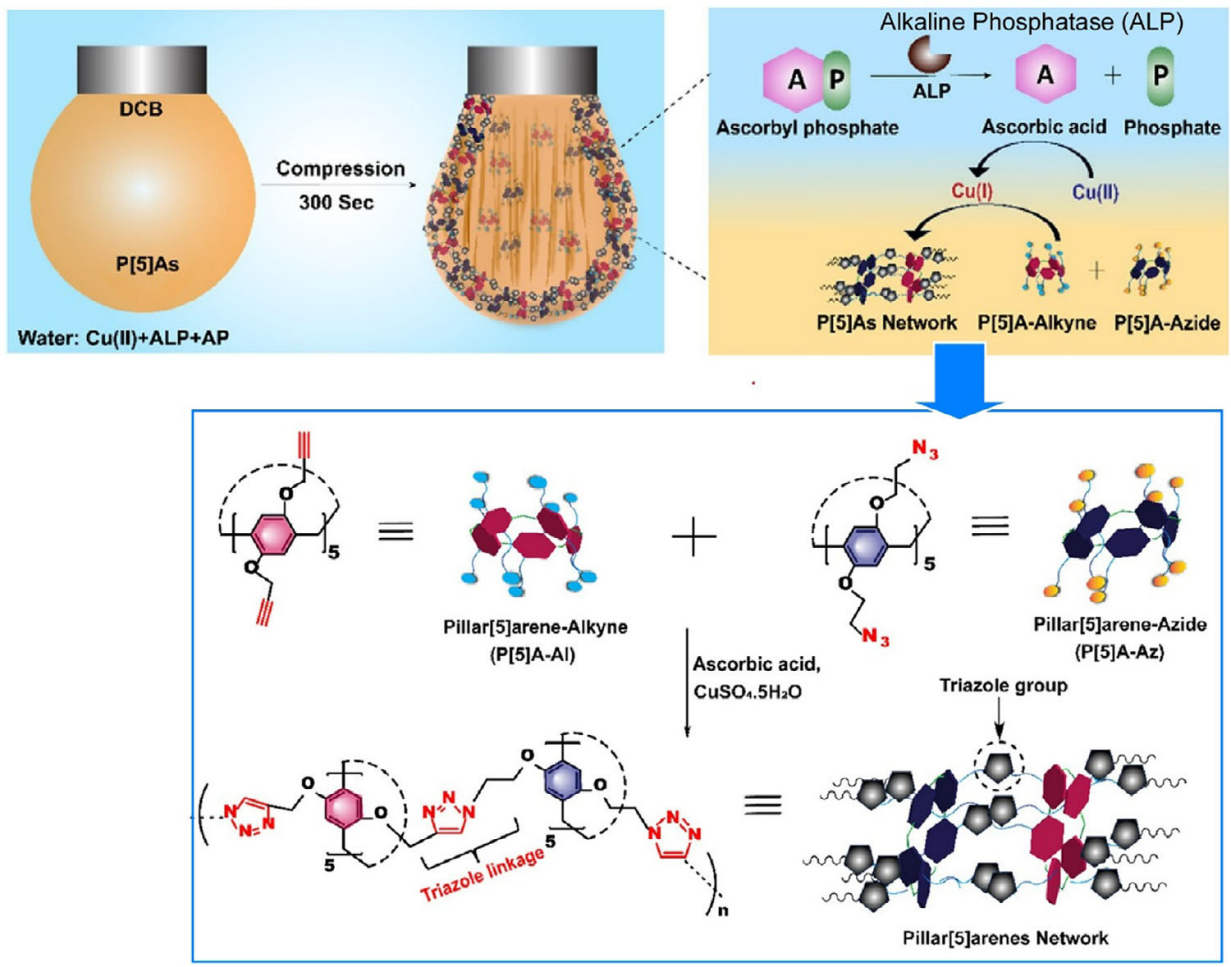
Self‐assembly and jamming of pillar[5]arene derivatives at the oil–water interface via a copper‐mediated click reaction Reprinted with permission from Ref. [86] Copyright 2025 American Chemical Society.

Attempts have also been made to couple liquid interface processes with biochemical reactions. Signal generation induced by enzymes at liquid–liquid interfaces can be a powerful strategy for controlling and detecting various processes. Patra and colleagues explored the self‐assembly and jamming of pillar[5]arene derivatives at the oil–water interface via a copper‐mediated click reaction (Figure 27).^[^ ^86^ ^]^ Introducing alkaline phosphatase as a trigger enables monitoring of enzyme activity through interfacial jamming and changes in surface coverage. The formation of pillar[5]arene networks at the droplet interface reduces interfacial tension and causes the droplets to adopt various non‐equilibrium shapes based on the interfacial jamming process. They introduced a signal amplification mechanism in which the dephosphorylation of dormant reductants by alkaline phosphatase triggers a click reaction at the interface. Furthermore, the sensitivity of this system to environmental changes can be tuned by inhibiting alkaline phosphatase with heavy metals and metal chelators. This system could provide a dynamic platform for translating molecular‐scale chemical reactions into detectable macroscopic changes. This paves the way for future applications in biochemical sensing at liquid–liquid interfaces, materials science, and environmental monitoring.

**Figure 28:**
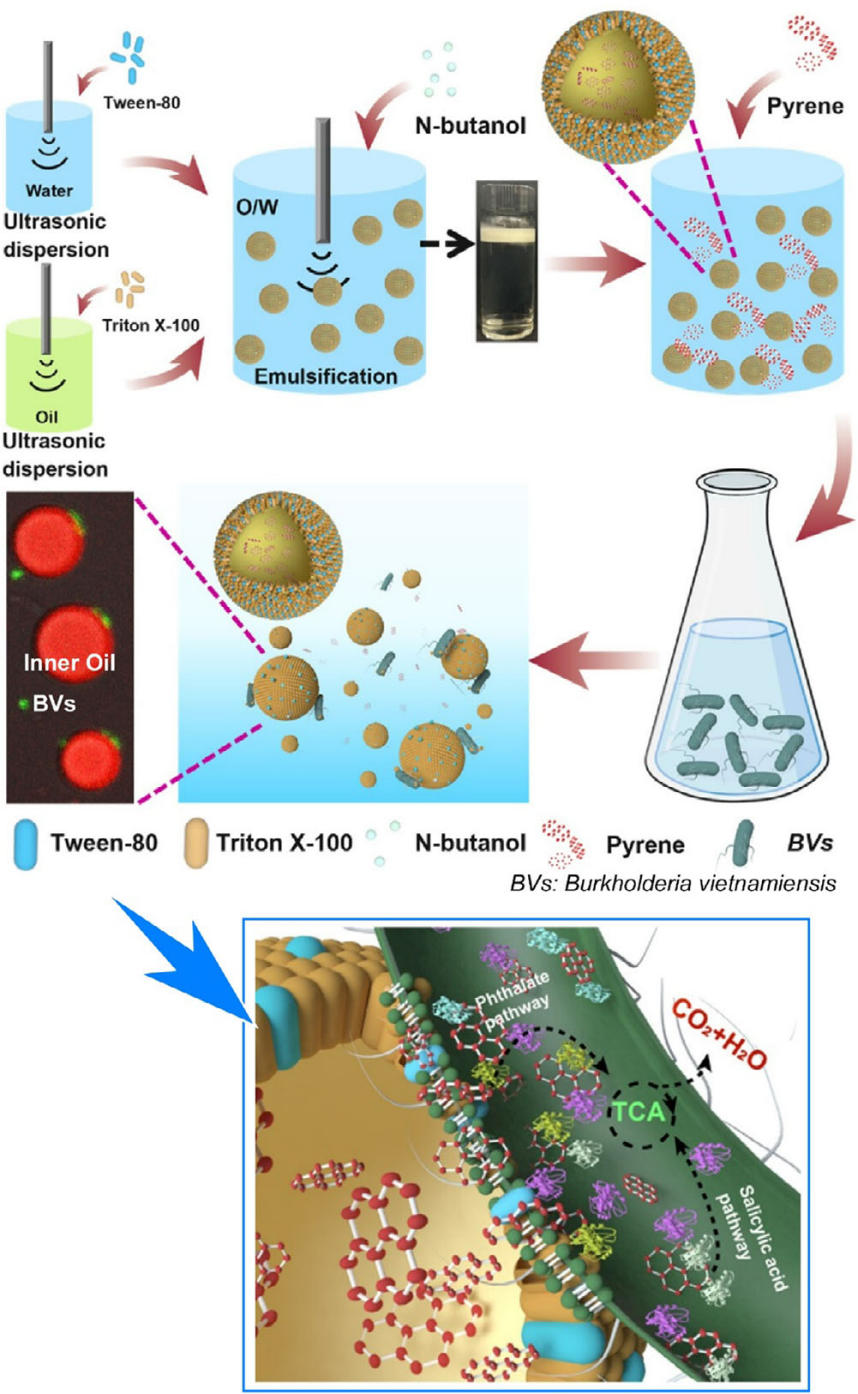
Preparation of an oil‐in‐water (O/W) macroemulsion bioreactor containing mixed surfactants, such as Tween 80 and Triton X‐100, as well as n‐butanol, corn oil, and Burkholderia vietnamensis bacteria, to enhance pyrene degradation efficiency. Reprinted with permission from Ref. [87] Copyright 2025 American Chemical Society.

The solubility of substances and molecules in immiscible liquids differs. Therefore, processes that overcome solubility in the solvent take place at the liquid–liquid interface. This process can be useful for the degradation of hazardous substances. High‐molecular‐weight polycyclic aromatic hydrocarbons have possible toxicities and are insoluble in aqueous solutions, which significantly limits their degradation efficiency in wastewater treatment and environmental remediation. To address this, Mi et al. designed an oil‐in‐water (O/W) macroemulsion bioreactor containing mixed surfactants, such as Tween 80 and Triton X‐100, as well as n‐butanol, corn oil, and *Burkholderia vietnamensis* bacteria, to enhance pyrene degradation efficiency (Figure 28).^[^ ^87^ ^]^ Pyrene was much more soluble in the prepared macroemulsion than in conventional surfactants due to the formation of an oil‐in‐water system. Due to pyrene's high solubility in the macroemulsion, it is easily adsorbed by the hydrophobic groups on the cell surface. Pyrene can easily penetrate the cell membrane and be transferred inside the bacteria. It is then degraded by a series of enzymes, including dehydrogenases, dioxidases, and isomerases. These enzymatic degradation reactions produce various by‐products, including 4,5‐pyrene dihydrodiol, dihydroxyphenanthrene, 1‐hydroxy‐2‐naphthoic acid, 2‐carboxybenzaldehyde, phthalic acid, and protocatechuic acid. These products are ultimately incorporated into the metabolic processes of microorganisms, providing them with energy via the tricarboxylic acid cycle. Designing and constructing reaction interface microenvironments in macroemulsions could be a viable approach for future biocatalysis and environmental remediation.

### 4.2 Cell Fate Manipulation

The aim of nanoarchitectonics is to build functional material systems from nanounits. This process is similar to the organization of advanced functional systems in living organisms. In other words, biological systems, such as cells, could be considered the ideal form of nanoarchitectonic products. The organization of cells can be considered a methodology for building more advanced functional systems. Various interfaces are used for this purpose. Liquid–solid interfaces are commonly employed,^[^ ^88^ ^]^ though research into manipulating cells at liquid–liquid interfaces is also emerging.^[^ ^89^ ^]^ The mechanical properties of the interface greatly influence cell proliferation and differentiation. Therefore, liquid–liquid interfaces are unique sites for controlling living cells.

**Figure 29:**
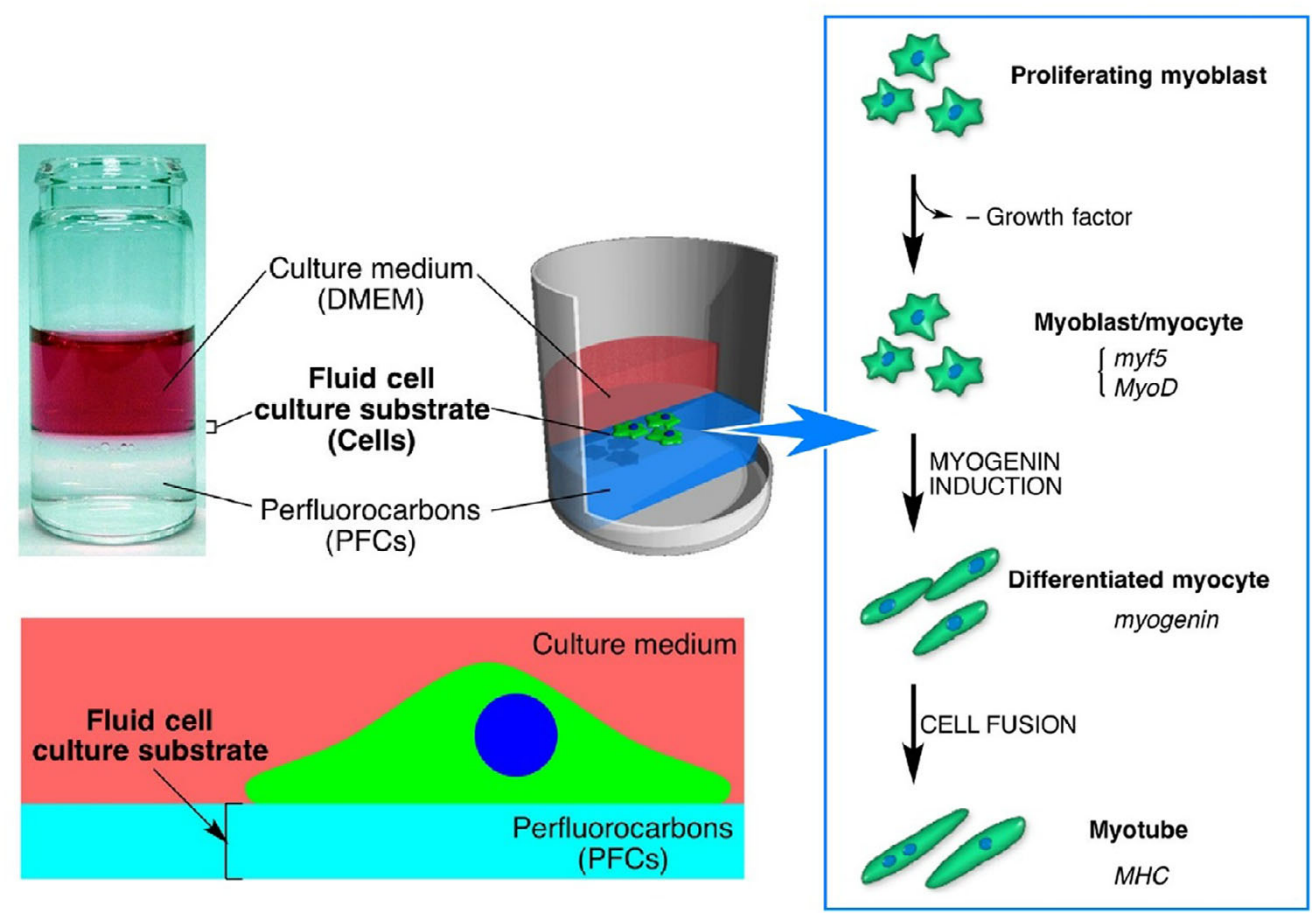
Control of muscle differentiation on a fluid substrate by using a water–perfluorocarbon interface as a scaffold, where C2C12 myoblasts were cultured and muscle differentiation was induced by reducing growth factor levels. Reprinted with permission from Ref. [90] Copyright 2017 American Chemical Society.

Cellular functions such as adhesion, proliferation, and differentiation depend on the stress relaxation properties of the viscoelastic substrate due to the physical microenvironment. The biological effects of fluid substrates, in which viscoelastic stress is virtually absent, have recently attracted attention. Minami et al. demonstrated control of muscle differentiation on a fluid substrate by using a liquid–liquid interface as a scaffold (Figure 29).^[^ ^90^ ^]^ For this purpose, C2C12 myoblasts were cultured in a fluid microenvironment at a water–perfluorocarbon interface. In this controlled in vitro culture, muscle differentiation was induced by reducing growth factor levels. In this case, the expression of myogenin, a gene in the myogenic regulatory factor family, was significantly reduced, whereas MyoD expression remained normal. The fluid microenvironment at the water–perfluorocarbon interface significantly reduced cell‐substrate interactions, thereby controlling cell differentiation within this culture system. A fluid microenvironment in which viscoelastic stress is virtually absent suppresses muscle differentiation. These results suggest that liquid–liquid interface culture systems could be useful in tissue engineering and mechanobiology. This approach also succeeded in transplanting cells cultured at such an interface using the LB method. Combining the interface culture system with the LB method makes it possible to investigate the effect of mechanical compression on cell function.

**Figure 30:**
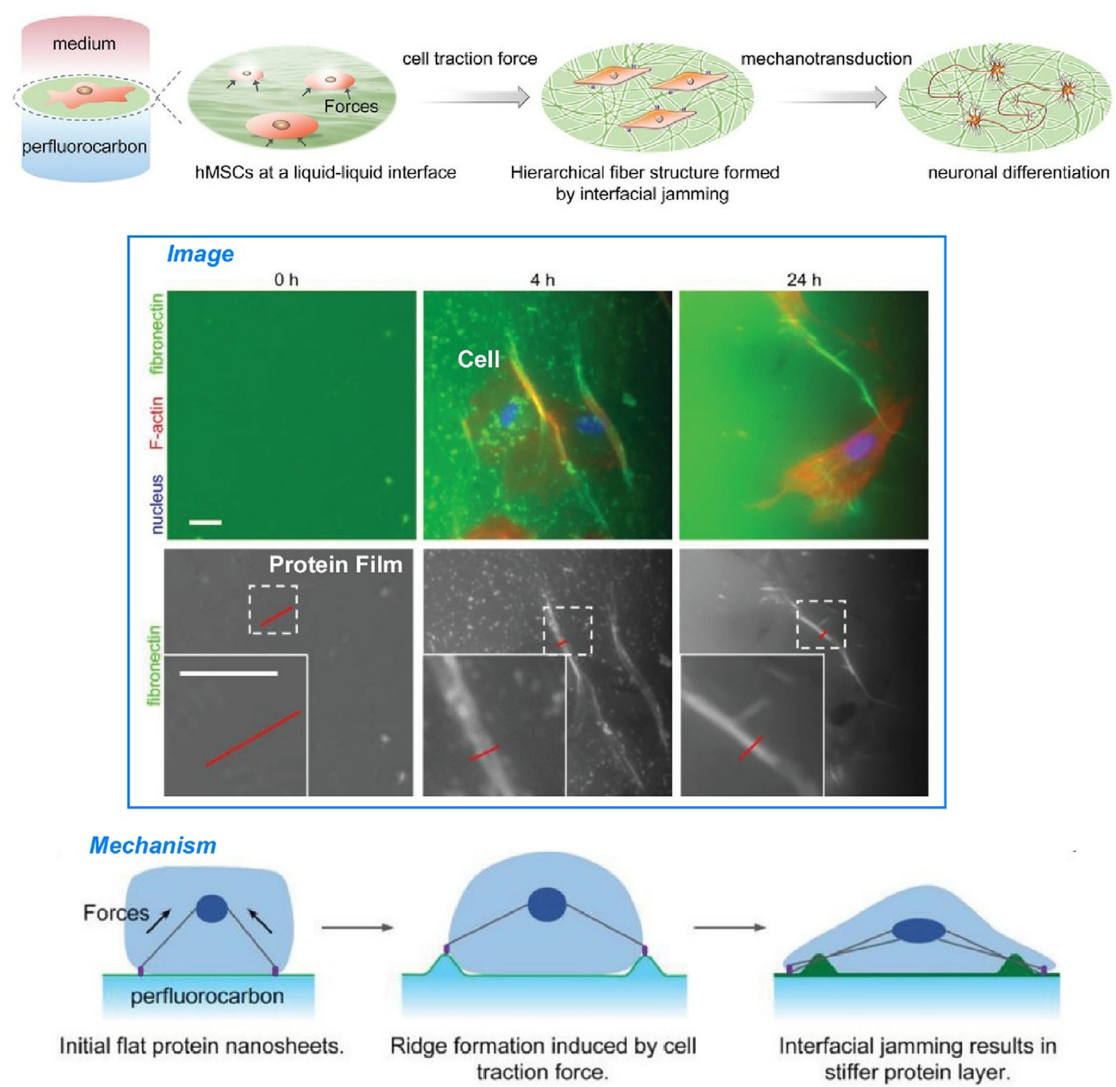
Control of human mesenchymal stem cell (hMSC) differentiation by an extracellular matrix mimic consisting of a protein monolayer formed at a liquid–liquid interface, where the protein monolayer serves as a tunable adaptive material that adapts dynamically to cell traction forces. Scale bars in images: 10 µm. Reprinted with permission from Ref. [91] Copyright 2019 Wiley‐VCH.

It is possible to mimic the native extracellular matrix in vivo and control cell fate at the liquid–liquid interface. Jia et al. investigated how human mesenchymal stem cell (hMSC) differentiation is controlled by an extracellular matrix mimic consisting of a protein monolayer formed at a liquid–liquid interface (Figure 30).^[^ ^91^ ^]^ This protein monolayer serves as a tunable adaptive material that adapts dynamically to cell traction forces. Hierarchical fiber structures of fibronectin assemblies form adjacent to hMSCs. In other words, the ultrastructural change from a protein monolayer to hierarchical fibers occurs due to interference with the cell interface. These elongated fibronectin fibers promote the formation of elongated focal adhesion structures, enhance the activation of focal adhesion kinase, and encourage the neural differentiation of stem cells via mechanotransduction signals. Consequently, cell traction forces lead to the spatial rearrangement of extracellular matrix proteins, which in turn affects stem cell fate. These results help to elucidate the feedback mechanisms linking dynamic extracellular matrix mechanics, biological signaling, and long‐term stem cell fate, and have broad implications for tissue engineering and regenerative medicine applications.

The physicochemical parameters of fluorocarbons and silicones used in liquid cell culture are narrow, and they may not be compatible with diverse cell culture environments. Ueki, Nakanishi, and their colleagues proposed that water‐immiscible ionic liquids could serve as cell culture liquids with tunable physicochemical properties and high solvation capacity.^[^ ^92^ ^]^ These ionic liquids are known as “designer solvents” due to the infinite combinations of chemical structures and ions. Customizing the structure of ionic liquids significantly expands the range of physicochemical parameters, such as polarity, viscosity, surface tension, and ionicity, compared to conventional liquids. Tetraalkylphosphonium‐based ionic liquids have been identified as non‐cytotoxic and are used for culturing hMSCs. Reducing the cationic charge distribution (i.e., ionicity) through alkyl chain extension enables cell spreading with mature focal contacts at the interface. Furthermore, this work fabricated ionic gels in which polymer networks were swollen with ionic liquids, taking advantage of the excellent solvent properties of ionic liquids. This demonstrates the controllability of bulk mechanics and interfaces that affect cell adhesion behavior. If this culture technique is established, it will dramatically improve culture efficiency compared to conventional 2D methods using plastic dishes. Cell resources can be recovered via a filtration process that does not require trypsin enzyme treatment, potentially facilitating the complete automation of the cell culture process. Using non‐flammable, non‐volatile, hydrophobic ionic liquids for this purpose would result in a recyclable, dry‐heat‐sterilizable dispersed phase.

**Figure 31:**
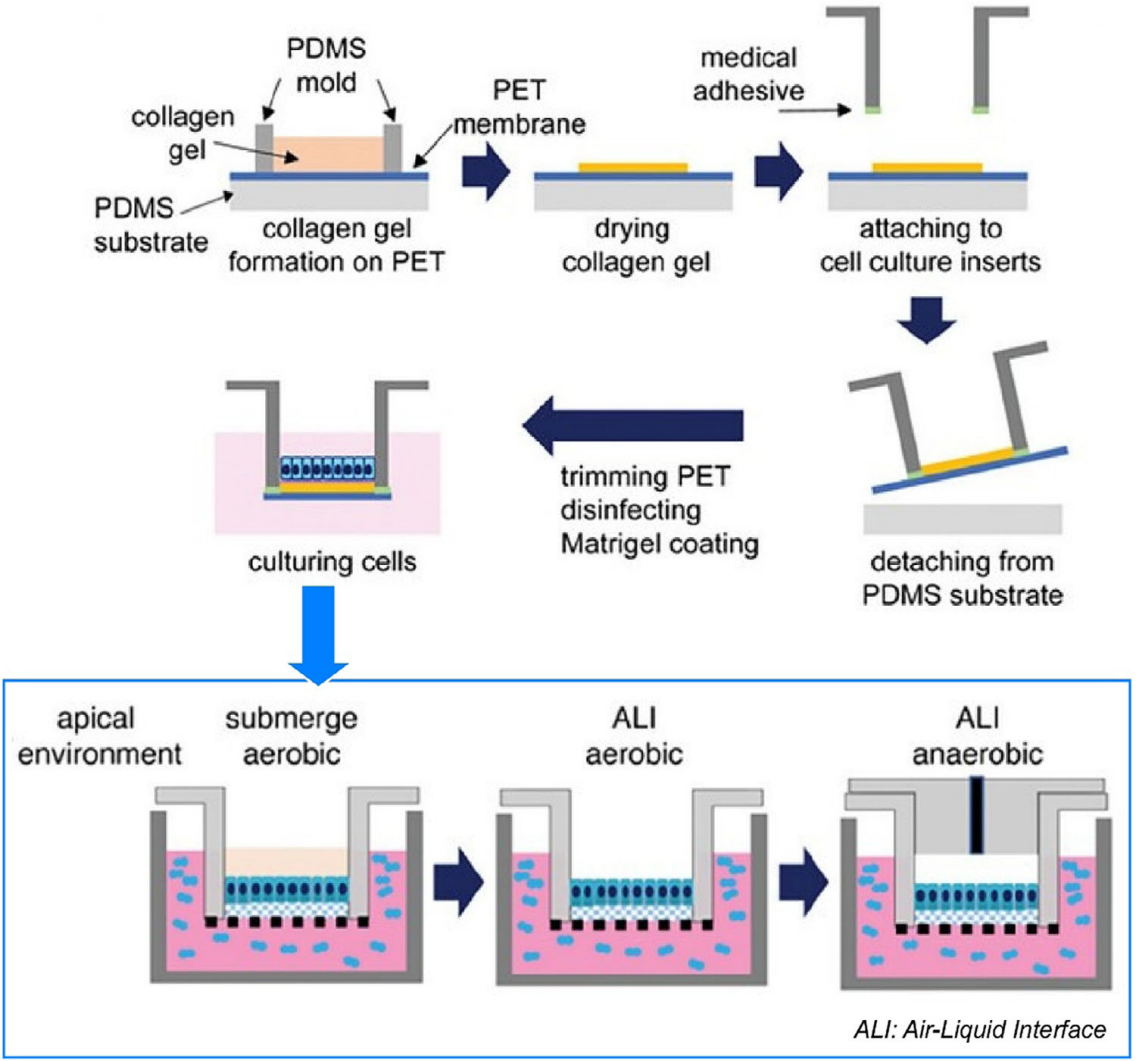
Cell‐derived functional mucus that closely resembles the in vivo colonic mucosa and apical microenvironment: fabrication of cell culture set‐up (top) and cell cultures under apical microenvironment (bottom). Reproduced under terms of the CC‐BY license from Ref. [93], 2024 Wiley‐VCH.

Gas–liquid interfaces are also used in cell research and organization. For example, such interfaces can be used as a system for forced cell multiplication. The co‐culture of enteric bacteria with primary human intestinal epithelial cells is useful for elucidating host‐colon bacterial interactions, as well as for testing and screening therapeutic drugs. Kim and Allbritton used anaerobic gas–liquid interface culture to construct a novel in vitro colonic microphysiological system with cell‐derived functional mucus that closely resembles the in vivo colonic mucosa and apical microenvironment (Figure 31).^[^ ^93^ ^]^ Primary human colonic epithelial cells were first cultured in a hanging basket format in submerged culture medium, then transferred to aerobic gas–liquid culture and finally to anaerobic gas–liquid culture. In the in vitro system, primary human colonic epithelial cells had a functional mucus layer with an average thickness of about 100 µm, as well as high cell viability (>98%). This methodology will be invaluable for future studies investigating the impact of resident and pathogenic bacteria on the colon. It may also provide a gut bacterial co‐culture platform that mimics the healthy colon microenvironment.

**Figure 32:**
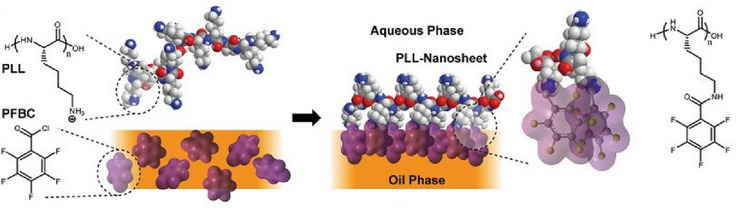
Macromolecular architecture of polymer nanosheets at liquid–liquid interfaces using poly(L‐lysine) with reactive co‐surfactants. Reproduced under terms of the CC‐BY license from Ref. [94], 2023 Wiley‐VCH.

Stem cells exert forces on the surrounding tissues. However, it is particularly challenging to separate toughness‐mediated cellular responses from other mechanosensory processes in the range of cell‐generated forces, especially when it comes to decoupling Young's modulus and shear modulus from toughness. Peng et al. demonstrated that the macromolecular architecture of polymer nanosheets can control interfacial toughness independently of the interfacial shear storage modulus, which controls the swelling of mesenchymal stem cells at liquid interfaces (Figure 32).^[^ ^94^ ^]^ Poly(L‐lysine) forms stiff nanosheets at liquid–liquid interfaces when combined with reactive co‐surfactants, such as pentafluorobenzoyl chloride. Interfacial rheology was employed to examine the adsorption and interfacial mechanical properties of nanosheets formed from poly(L‐lysine) with various molecular weights. Interfacial microrheology using magnetic tweezers revealed the local mechanical properties of the nanosheets, and in situ ellipsometry measured the thickness of these assemblies. Interfacial shear rheology was used to characterize the viscoelasticity and toughness of poly(L‐lysine) nanosheets assembled at liquid–liquid interfaces. The ability of cells to sense the mechanical properties of their surroundings is enabled by adhesion mechanisms involving integrin binding, actin assembly, and contractility. These mechanisms are mediated by adaptor proteins such as actin and vinculin, and interact with the nanoscale mechanics of the corresponding interface. The viscoelastic profile of the corresponding material is a crucial element sensed by cell adhesion and triggers downstream signaling. In this study, they demonstrate that interfacial toughness can modulate these processes further.

**Figure 33:**
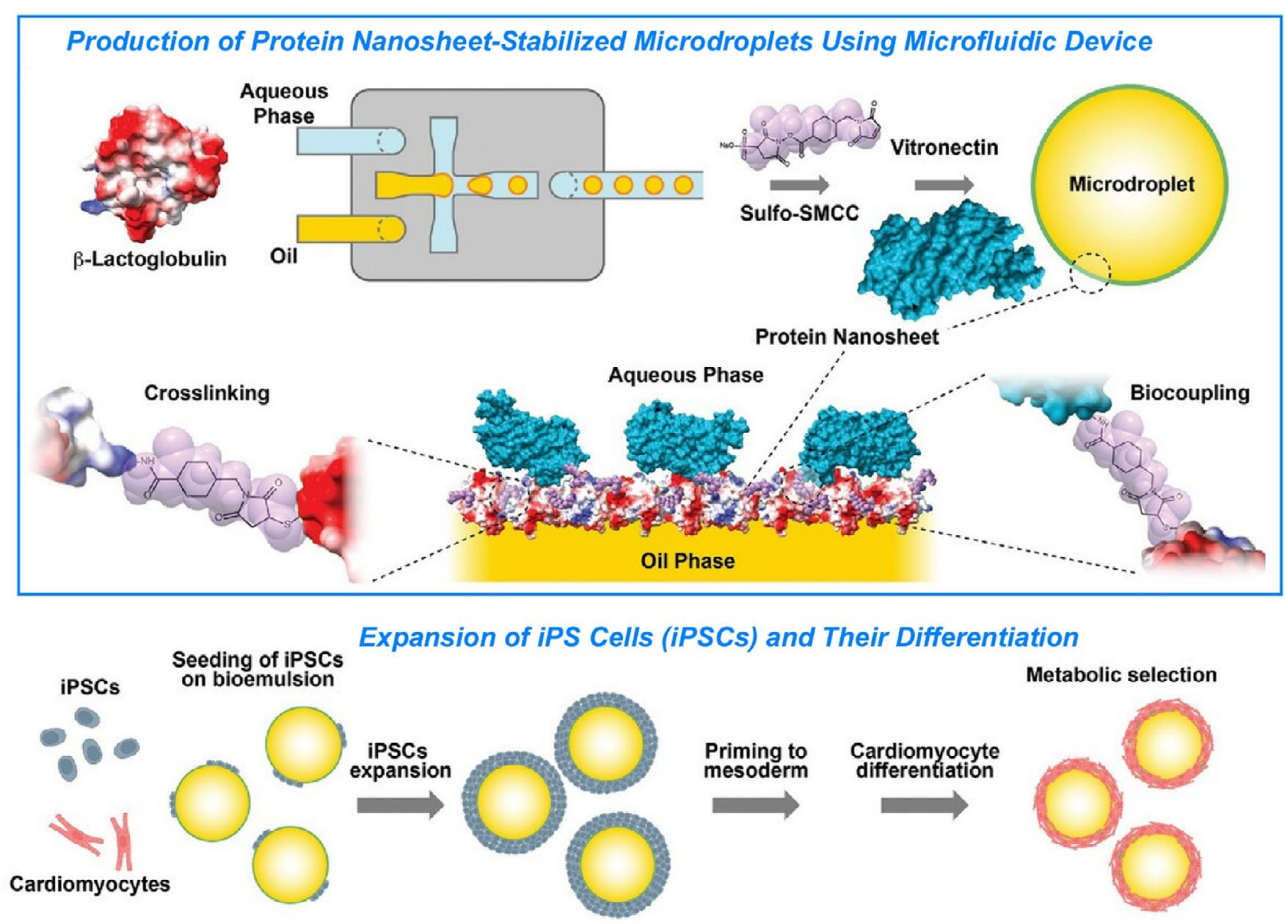
Protein nanosheets that stabilize liquid–liquid interfaces and enable induced pluripotent stem cells (iPSCs) to adhere, grow, and retain their stem cell properties: production of protein nanosheet‐stabilized microdroplets using a microfluidic device (top) and expansion of iPSCs and their differentiation (bottom). Reproduced under terms of the CC‐BY license from Ref. [95], 2024 Wiley‐VCH.

Advances in stem cell technology necessitate the development of new cell manufacturing pipelines that can be scaled up and parallelized. However, the soft mechanical properties of liquid–liquid interfaces generally prevent the growth and differentiation of induced pluripotent stem cells (iPSCs). Gautrot et al. describe the design of protein nanosheets that stabilize liquid–liquid interfaces and enable iPSCs to adhere, grow, and retain their stem cell properties (Figure 33).^[^ ^95^ ^]^ These nanosheets are assembled from the globular protein β‐lactoglobulin. Incorporation of the heterobifunctional crosslinker sulfo‐SMCC enables covalent bioconjugation with vitronectin, thereby modulating the interfacial mechanical properties and facilitating biofunctionalization. iPSCs cultured under these conditions retain their ability to differentiate into cardiomyocytes. The obtained results clearly demonstrate that local nanoscale mechanics associated with interfacial viscoelasticity are powerful signals that support the control and maintenance of pluripotency, as well as the commitment to specific differentiation conditions. In this study, they used a low‐viscosity, oil‐based substrate for iPSCs culture. However, the strong elastic interfacial shear mechanics generated by the assembly and cross‐linking of protein nanosheets enable resistance to the forces exerted by cells during colony formation. This work demonstrates that similar crosstalk between cell–cell and cell–matrix adhesion controls processes such as differentiation and motility on the surface of flexible hydrogels. Bioemulsions offer a unique opportunity to transform cell manufacturing and delivery processes and protocols.

**Figure 34:**
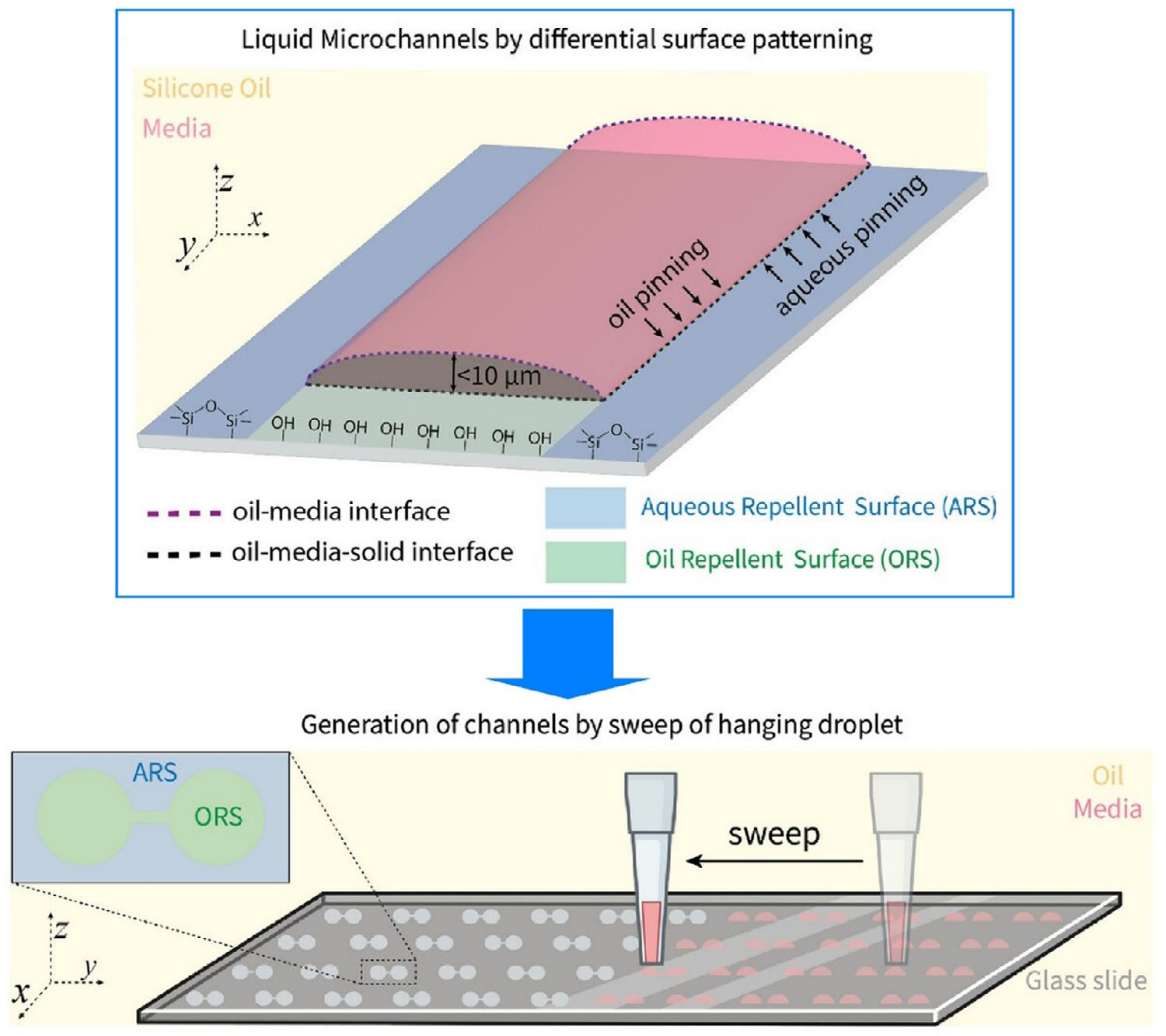
Preparation of microchannels with liquid–liquid interfaces that exert confining pressures similar to those exerted by cells in tissues using differential surface patterning of glass substrates to design microchannels on a scale comparable to that of a single cell bounded by a liquid–liquid interface. Reproduced under terms of the CC‐BY license from Ref. [96], 2025 Wiley‐VCH.

The control of cells at liquid–liquid interfaces has been further developed in combination with microfabrication techniques. Leukocytes carve out a path through the interstitial space by overcoming the mechanical pressure exerted by surrounding cells. Designing in vitro systems that model the deformable cellular environment encountered in vivo is an attractive prospect. Li, Huttenlocher, Beebe, and colleagues construct microchannels with liquid–liquid interfaces that exert confining pressures similar to those exerted by cells in tissues.^[^ ^96^ ^]^ They also derive material interfaces that reproduce the confining pressures exerted by the soft tissue environment (Figure 34). Using differential surface patterning of glass substrates, they designed microchannels on a scale comparable to that of a single cell (i.e., channel height < 10 µm), which are bounded by a liquid–liquid interface. They implemented a simple pattern that produced two hemispherical droplets connected by a single linear liquid channel. They investigated how primary neutrophils—the most abundant cells in the immune system, and a classic model of amoeboid cell migration—interact with liquid–liquid interfaces during chemotaxis. The interface exerts comparable pressure on the cells themselves. In liquid microchannels, larger interfacial deformations are required to provide space for migration. Increasing the confining pressure by adjusting the channel geometry affects motility. The interface is rigid enough to confine cells yet deformable in response to the forces generated by individual cells. Consequently, motile cells have great autonomy in controlling the degree of confinement and thus their morphology. Further elucidation of how immune cells sense and respond to physiologically relevant physical signals could facilitate the development of therapeutics that target immune cell migration mechanisms in human diseases.

As demonstrated in this section, the liquid–liquid interface facilitates dynamic organization. It also facilitates the association of substrates dissolved in two immiscible liquids. As this environment is confined to the interface, the resulting structures are often nanometer‐thick films. The phenomenon of assembly at an interface is universal, regardless of the type of molecule or material involved. Nanoarchitectonics at liquid interfaces have a wide range of applications, from simple molecules to complex biological systems, including single‐element zero‐dimensional molecules such as fullerenes, polymers, nanosheets, proteins, and cells. Previous examples have shown that organization can be formed using the same range of components, but this does not demonstrate the full potential of liquid interface nanoarchitectonics. It is expected that this technique will be used to develop complex functional materials, in which a wide variety of components are assembled and organized together at the interface from both liquid phases.

## 5 General Trends of Nanoarchitectonics Research

In the preceding sections, we have explored several themes and objectives, providing an overview of nanoarchitectonics and associated research. Even within these limited examples, the molecules, materials, and cells that are of interest are highly diverse, as are the related properties and functions. A wide range of phenomena is involved in terms of scale, ranging from atomic and molecular sizes to cellular and visible levels. The concept of nanoarchitectonics is very general and universal, and therefore applicable to a wide range of areas. In this section, we will briefly describe such general trends.

To do so, we will examine papers with the word “nanoarchitectonics” in the title to identify trends. Papers containing the term “nanoarchitectonics” in the title began to be published in 2003, with over 10 papers published per year in the 2010s and over 100 papers in the 2020s. These papers have also received a growing number of citations in recent years, and the fields they cover are wide‐ranging. Papers on the synthesis of functional materials^[^ ^97^ ^]^ and the creation of defined structures^[^ ^98^ ^]^—the basis of nanoarchitectonics—are constantly being published. In a recent review titled “Chemical programming for micro‐ and nanoarchitectonics of 3D/4D‐printed thermoelectric materials”, Sonigara and Pumera discuss the development of thermoelectric materials suitable for 3D/4D printing technology.^[^ ^99^ ^]^ Additionally, Yoon and Jackman, in their review paper titled “Medium‐chain fatty acids and monoglycerides: Nanoarchitectonics‐based insights into molecular self‐assembly, membrane interactions, and applications”, discuss the concept of medium‐chain fatty acids and monoglycerides as major organizers of phospholipid membranes and other membrane interactions from the perspective of bottom‐up nanoarchitectonics.^[^ ^100^ ^]^

Studies that utilize nanoarchitectonics‐based structure formation are also conducted in fundamental research. In addition to property explorations in basic sciences such as chemistry^[^ ^101^ ^]^ and biochemical research,^[^ ^102^ ^]^ nanoarchitectonics is also used in fundamental physics.^[^ ^103^ ^]^ In a review article titled “Nanoarchitectonics for regulating molecular conductance by multi‐channel structure”, Yang, Zhang, and colleagues discuss the methodology for studying molecular conductance properties and the factors that affect conductance.^[^ ^104^ ^]^ To this end, nanoarchitectonics at the molecular level is required to immobilize target molecules appropriately between electrodes or probes. The enhancement of molecular conductance is said to be due to factors such as the superposition law based on quantum interference, the charge‐induced self‐gating effect, and the promotion of additional conduction channels by space‐mediated transport.

Of course, the construction of nanoarchitectonics‐based functional structures is useful for a variety of applications. This concept is employed in many different fields, such as catalysis,^[^ ^105^ ^]^ sensing,^[^ ^106^ ^]^ device development,^[^ ^107^ ^]^ environmental technologies,^[^ ^108^ ^]^ fuel cells,^[^ ^109^ ^]^ solar cells,^[^ ^110^ ^]^ other energy‐related technologies,^[^ ^111^ ^]^ drug delivery^[^ ^112^ ^]^ and biomedical applications.^[^ ^113^ ^]^ Some examples of recent papers are given below. In the paper “Nanoarchitectonics of porous carbon derived from urea‐impregnated microporous triazine polymer in KOH activator for adsorptive removal of sulfonamides from water”, Ahmed and Jhung constructed highly porous carbon materials through the pyrolysis of covalently bonded organic polymers, successfully adsorbing and removing sulfamethoxazole and sulfachloropyridazine from water in the process.^[^ ^114^ ^]^ Similarly, in the paper “Lignite‐based nanoarchitectonics with N self‐doped and oxygen‐rich hierarchically porous activated carbons for high performance supercapacitors”, Luo et al. modified carbon materials and investigated their supercapacitor properties.^[^ ^115^ ^]^ In the paper “Iron N‐doped carbon nanoarchitectonics for C─H bond activation of methylarenes and esterification reactions”, Gawande and colleagues investigated the efficiency of iron nanoparticle catalysts hybridized with nitrogen‐doped carbon in C─H activation and esterification reactions.^[^ ^116^ ^]^ In the review paper, “Nanoarchitectonics for pentagon defects in carbon: Properties and catalytic role in oxygen reduction reaction”, Chen et al. discuss the fundamental principles governing the catalytic activity of the oxygen reduction reaction involving pentagons, combining experimental and theoretical insights into nanoarchitectonics.^[^ ^117^ ^]^

In the research paper titled “Supraparticle nanoarchitectonics with bright gold nanoclusters induced by host–guest recognition for volatile amine and meat freshness detection”, Peng et al. synthesized hybrids of 6‐aza‐2‐thiothymine‐capped gold nanoparticles and macrocyclic cucurbit[n]urils (CB[n]) (*n* = 7 and 8), demonstrating their potential for ammonia detection and food freshness monitoring through fluorescence analysis.^[^ ^118^ ^]^ In the review paper, “Two‐dimensional nanozyme nanoarchitectonics customized electrochemical bio diagnostics and lab‐on‐chip devices for biomarker detection”, Chang et al. extensively explore the latest advances in 2D nanozyme‐based electrochemical biosensors and lab‐on‐chip devices.^[^ ^107d^ ^]^ In the study titled “Nanoarchitectonics of high‐performance and flexible n‐type organic–inorganic composite thermoelectric fibers for wearable electronics”, Cai and colleagues fabricated PEDOT:PSS/PC‐Ag_2_Te nanowire composite fibers as thermoelectric fibers and explored their application in wearable electronics.^[^ ^119^ ^]^ In the paper titled “Liposomal‐based nanoarchitectonics as bispecific T cell engagers in neuroblastoma therapy”, Parra‐Nieto et al. explored the path to effective neuroblastoma therapy based on liposomes and protocells dual‐functionalized with *p*‐aminobenzylguanidine and fluorescein, which selectively bind synthetic targeting molecules.^[^ ^120^ ^]^

The papers presented here represent only a small fraction of the nanoarchitectonics papers published in 2025. The materials used, fabrication methods and target functions are all highly diverse. The concept of nanoarchitectonics is extremely broad, with no restrictions on target substances or applications. This is also true of the preceding concept of nanotechnology. Nanoarchitectonics involves constructing functional substances from nanounits, such as atoms and molecules. Given that all substances are composed of atoms and molecules, nanoarchitectonics can be considered a methodology ultimately involved in the production of all substances. If super unification theory is theory of everything as the ultimate principle of physics,^[^ ^121^ ^]^ nanoarchitectonics could be considered a method for everything in materials science.^[^ ^122^ ^]^

## 6 Emerging Topics

As outlined in previous sections, nanoarchitectonics approaches are highly diverse and offer significant potential. Furthermore, new approaches continue to be devised, thanks to the flexibility of the concept. The possibilities are enormous. In the final section, we will explore some emerging topics in more detail. The keyword here is “dynamic behavior”. Adding dynamic elements will further expand the possibilities of nanoarchitectonics. In this section, we will consider two topics: “vortex nanoarchitectonics at interfaces” and “controlled molecular conversion to structure‐defined materials”, exploring new possibilities.

### 6.1 Vortex Nanoarchitectonics at Interfaces

Due to the high degree of freedom of movement of the components located there, liquid interfaces provide a wide variety of nanoarchitectonics. Introducing an artificial flow into the interface creates a new mode of nanoarchitectonics that combines the action of external forces with spontaneous organization.^[^ ^123^ ^]^ For instance, stirring the liquid phase with a rotational force creates a vortex on the surface of the liquid, causing molecules to accumulate while being subjected to the perturbation. We will present several examples of liquid interface nanoarchitectonics under vortex flows.

**Figure 35:**
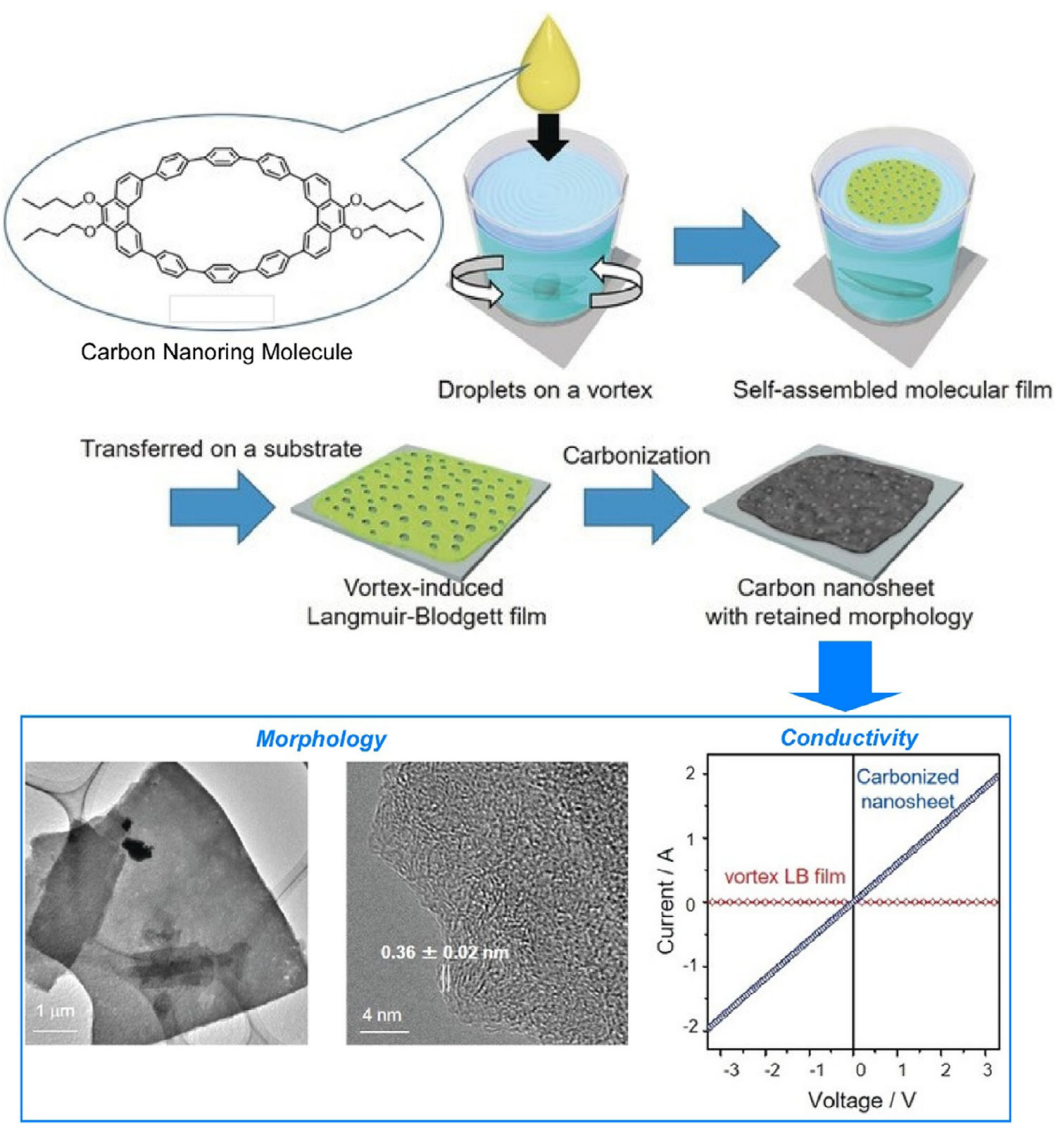
Bottom‐up carbon nanosheet fabrication using a carbon‐rich π‐conjugated, anisotropic, macrocyclic molecule: 9,9“,10,10″‐tetrabutoxycyclo[6]‐paraphenylene[2]‐3,6‐phenanthrenylene (a carbon nanoring molecule) that were spread on a dynamic air‐water interface with vortex motion to form molecular nanosheets through self‐organization, followed by carbonization with heat treatment at 850 °C: preparation process (top) and properties (bottom). Reprinted with permission from Ref. [124] Copyright 2018 Wiley‐VCH.

2D carbon nanomaterials are expected to have a variety of applications, including high‐performance energy storage devices and catalysts. To this end, it is important to be able to synthesize the required quantity using a simple process. The nanoarchitectonics of 2D carbon with a thickness at the nanometer level by wet processing is a very attractive research target. Mori et al. reported a new bottom‐up carbon nanosheet fabrication method that uses a carbon‐rich π‐conjugated, anisotropic, macrocyclic molecule: 9,9“,10,10”‐tetrabutoxycyclo[6]‐paraphenylene[2]‐3,6‐phenanthrenylene (a carbon nanoring molecule) (Figure 35).^[^ ^124^ ^]^ The carbon nanoring molecules were spread on a dynamic air‐water interface with vortex motion, and the molecular nanosheets were formed through self‐organization. It is thought that the elliptical carbon nanoring molecules are stacked under the force of the flow to form a 2D sheet. This molecular sheet was transferred to a silicon substrate using the LB method and carbonized by heat treatment at 850 °C for 3 h in a nitrogen gas flow. A large carbon nanosheet was obtained. The molecular sheet's shape before calcination was largely maintained, and a uniform molecular thin film with a thickness of less than 10 nm and an area of 1000 µm^2^ was successfully obtained. The carbon nanosheet has a mesoporous structure, with pores measuring 20–30 nm in diameter on its surface. A high‐resolution TEM image of the carbonized nanosheet revealed a turbostratic carbon structure, characterized by randomly stacked curved carbon atom layers. The distance between these layers was approximately 0.36 nm, which is close to the interplanar spacing of graphite. Furthermore, carbonization significantly improved the electrical conductivity. N‐doped carbon nanosheets can easily be produced by adding pyridine as a dopant to the initial carbon nanoring molecular solution, forming a thin film using the vortex method, and then carbonizing it. The amount of pyridinic nitrogen species doped into the resulting 2D nanosheet was high. It is therefore anticipated that N‐doped carbon nanosheets could be employed as highly efficient catalysts in the oxygen reduction reaction of high‐performance fuel cells. Only a very small quantity of carbon nanorings—just 1 ng—is needed to produce a 1 m^2^ nanosheet. Increasing the area makes it an industrially deployable technology. This groundbreaking technique has significant implications for the nanoarchitectonics of 2D carbon materials.

**Figure 36:**
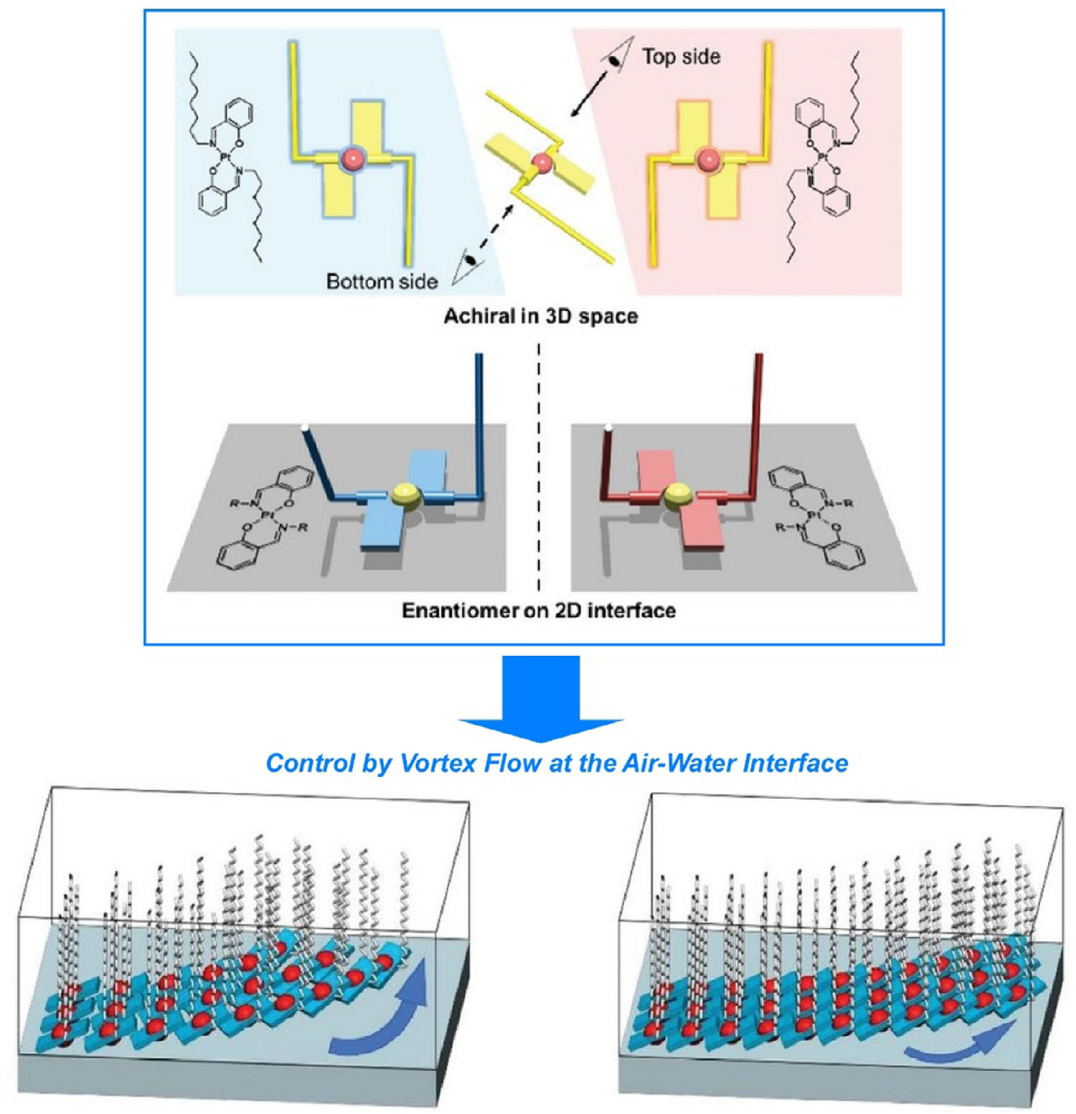
Precise control of chiral structures and properties through the nanoarchitectonics of complex molecules (trans‐bis(salicylaldiminato)platinum(II) complexes bearing hexadecyl groups) under vortex flow conditions at the air–water interface. Reprinted with permission from Ref. [125] Copyright 2022 Wiley‐VCH.

Circularly polarized luminescence is expected to have various applications, with properties such as chirality and luminescence intensity being controlled. However, it is challenging to control the circularly polarized luminescence precisely and reproducibly due to supramolecular chirality. Maeda et al. have reported precise control of circularly polarized luminescence through the nanoarchitectonics of complex molecules (*trans*‐bis(salicylaldiminato)platinum(II) complexes bearing hexadecyl groups) under vortex flow conditions at the air–water interface (Figure 36).^[^ ^125^ ^]^ In the absence of vortex flow, a microcrystalline/amorphous solid forms through thermodynamically controlled, three‐dimensional aggregation based on a lamellar arrangement of cross‐shaped molecular units. Intermolecular interactions and perfect stacking induce the dispersion of light energy in the excited state, resulting in the aggregates nearly non‐luminescent properties. Conversely, under suitable vortexing conditions, the molecules form 2D aggregates with chiral U‐shaped conformations at the air–water interface. The helical twisting force of the vortex flow imparts supramolecular chirality to these 2D domains. This is generated by a unidirectional twist in the stacking of the coordination planes, with the degree of twist being controlled by the speed of the vortex flow. The transfer of chirality from unidirectional vortex flow enables the polarity and intensity of the aggregates' chiroptical properties to be precisely controlled by altering the direction and speed of the vortex flow in the water layer. The chirality can be reversed by the opposite vortex flow rotation. Depending on the vortex flow conditions, the direction and emission intensity of the circularly polarized emission of the Pt(II) complex assemblies can be precisely tuned. In other words, as the vortex flow speed increases, the vortex flow‐induced emission also increases. This emission enhancement is attributed to a change in the stacking state of the coordination planes caused by the vortex flow speed. The faster the vortex flows, the greater the slippage and the more helical the 2D molecular arrangement formed. Therefore, the shallower the stacking of the coordination planes, the more the nonradiative decay is suppressed based on deeper stacking. This new nanoarchitectonics method shows that circularly polarized luminescence can be easily and precisely controlled by macroscopic action at the air–water interface.

**Figure 37:**
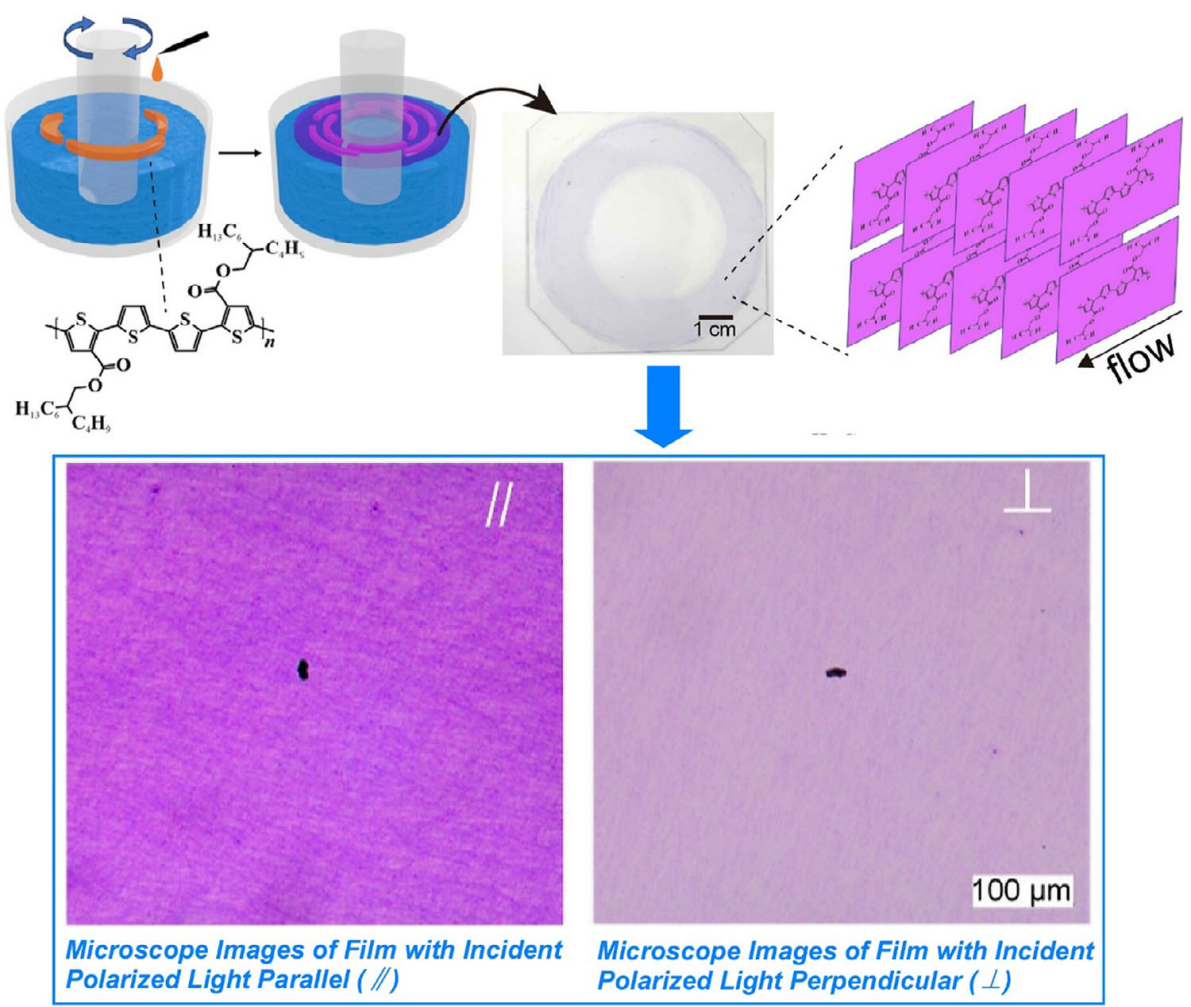
A circular flow orientation method for polymer semiconductor thin films in which a polymer semiconductor solution is dropped onto a glycerol liquid under moving in a circular motion within a container to give a thin film with orientation along the flow direction. Reprinted with permission from Ref. [126] Copyright 2025 American Chemical Society.

Nanoarchitectonics based on liquid flow at the interface can also be used to control the orientation of polymer semiconductors. Controlling the orientation of polymer semiconductors is fundamental to understanding and improving carrier transport properties. Polymer semiconductor thin films can be fabricated using simple solution processes, such as spin coating and casting. However, complex convection during solvent evaporation negatively affects orientation control in these methods. Fujioka et al. developed a circular flow orientation method for polymer semiconductor thin films.^[^ ^126^ ^]^ As illustrated in Figure 37, a polymer semiconductor solution is dropped onto a glycerol liquid that is moving in a circular motion within a container. A thin film then forms at the air‐liquid interface with a specific flow rate. The main chains of the resulting thin film are oriented along the flow direction, and the influence of convection during solvent evaporation is suppressed. This method utilizes the liquid flow at the air‐liquid interface efficiently to fabricate an oriented thin film with a 2D packing structure. The resulting thin film is a highly oriented, edge‐on ultrathin polymer semiconductor film. The molecular orientation of the resulting film is parallel to the liquid flow. In a field‐effect transistor using this thin film, the mobility was four times higher than in a device made using a spin‐coated thin film. This research provides a simple method to suppress convection effects during solution processing. It can be scaled up by adjusting the flow channel design and is expected to contribute to a wide range of oriented polymer semiconductor thin film applications.

The flow‐based nanoarchitectonics method presented here exploits the free movement and deformability of liquid interfaces. It can be used to create functionally superior structures, such as 2D nanocarbon thin films with controlled structure, supramolecular thin films with chiral optical properties, and polymeric semiconductor thin films with highly controlled orientation. These methods have one key feature in common: molecular‐level structure and orientation can be precisely controlled through visible movements, such as vortices on the surface of water. The ability to control molecular‐level structures with macroscopic movements suitable for mass production paves the way for nanoarchitectonics to be applied in industry.

### 6.2 Controlled Molecular Conversion to Structure‐Defined Materials

The rational and controlled manipulation of structures is an important theme in nanoarchitectonics. Molecular structures can be controlled through organic synthesis or surface‐based synthesis, which combines nanotechnology. Larger structures can be controlled through molecular assembly and associated material transformation. These approaches tend to be specific to the individual case. A more systematic example is the control of mesoscopic morphology and molecular framework‐level structure starting from the same molecule, fullerene C_60_. This demonstrates the diverse outcomes of nanoarchitectonics. Examples of this are presented in the following sections.

**Figure 38:**
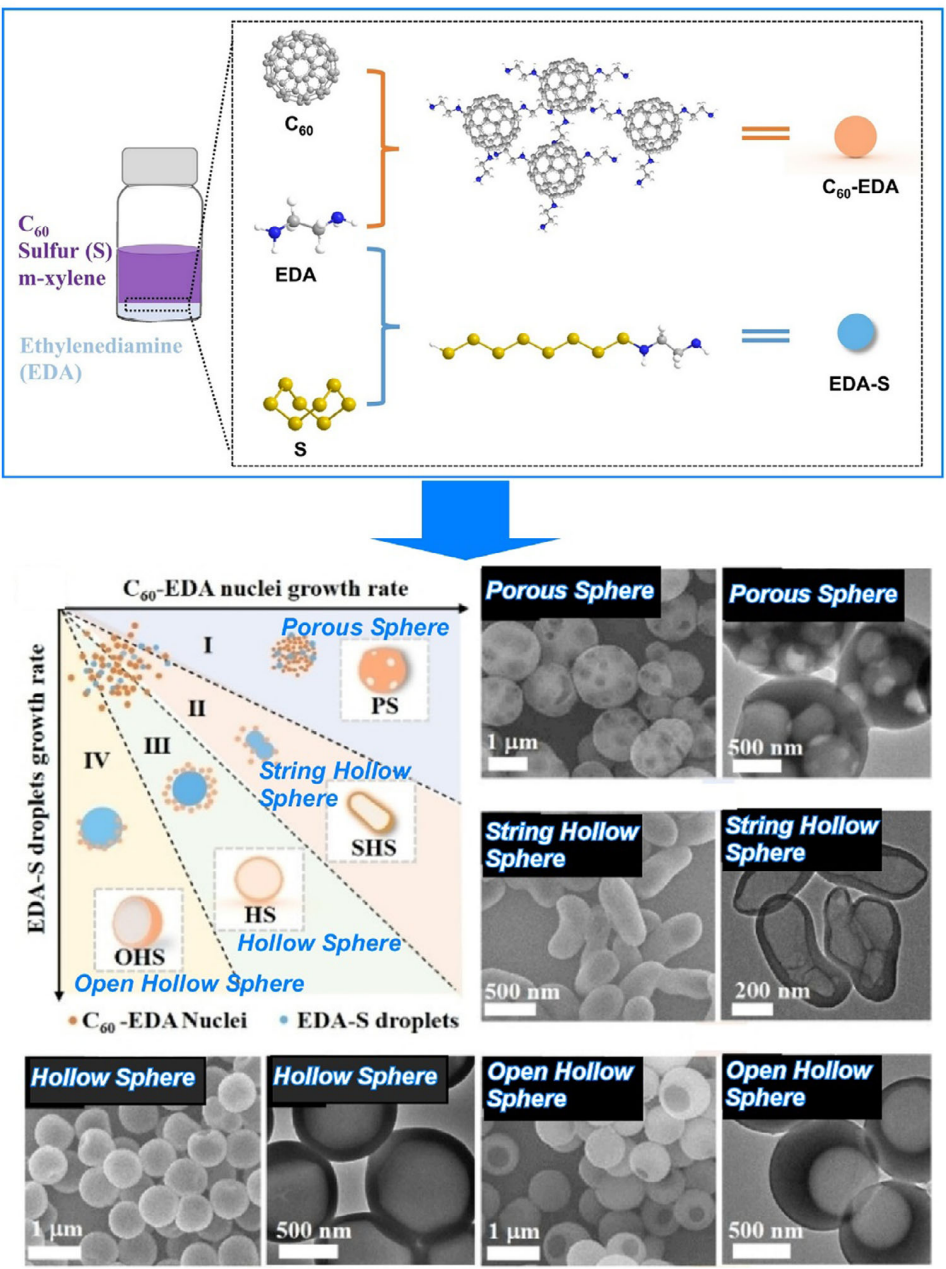
A methodology for creating hollow structures from fullerene (C_60_) using the kinetically controlled liquid–liquid interfacial precipitation (KC‐LLIP) method, where controlling the nucleation rate of C_60_‐EDA (ethylenediamine) and the growth of the template EDA‐S allows porous spheres, string‐like hollow spheres, hollow spheres, and open‐type hollow spheres to be synthesized. Reprinted with permission from Ref. [127] Copyright 2022 Wiley‐VCH.

Nanomaterials with hollow structures are expected to exhibit new functionalities in the field of materials engineering. It would be highly advantageous if hollow structures of various shapes could be easily produced by altering the conditions. Chen et al. demonstrated a methodology for creating hollow structures from fullerene (C_60_) using the kinetically controlled liquid–liquid interfacial precipitation (KC‐LLIP) method. Depending on the conditions, hollow structures of various shapes can be obtained, as shown in Figure 38.^[^ ^127^ ^]^ For this purpose, EDA was used as a covalent crosslinking agent for C_60_ molecules to form a C_60_‐EDA shell. At the same time, in situ generated EDA‐sulfur (EDA‐S) droplets were applied as an “egg yolk” and removed by washing after formation of the “egg yolk” shell structure, creating hollow structures. Controlling the nucleation rate of C_60_‐EDA and the growth of the template EDA‐S allows porous spheres, string‐like hollow spheres, hollow spheres, and open‐type hollow spheres to be synthesized. Isopropanol was used as an additive to control the difference in growth rate between the C_60_‐EDA and the EDA‐S. This method can easily be applied to large‐scale experiments and produces well‐defined C_60_ nanospheres with various controllable hollow morphologies that are suitable for technological applications. Additionally, annealing the C_60_ nanospheres is beneficial for developing nanocarbon materials of various shapes.

**Figure 39:**
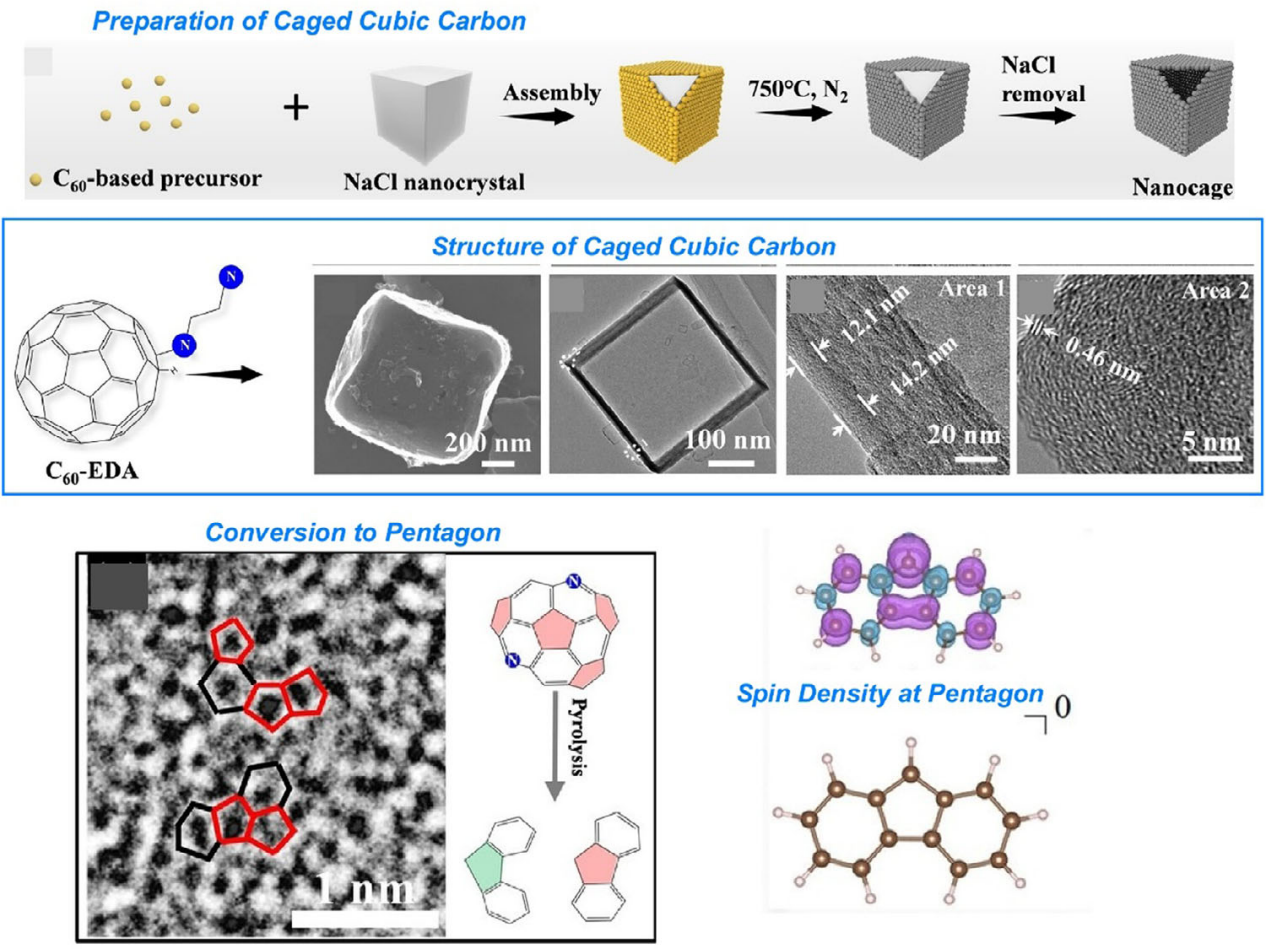
Fabrication of caged cubic carbon catalysts rich in pentagon structures, where bonded pentagon structures underwent changes during pyrolysis due to nitrogen doping, resulting in pentagon defects. Reproduced under terms of the CC‐BY license from Ref. [128], 2024 Wiley‐VCH.

Conversely, the nanoarchitectonics of structures smaller than fullerenes can be achieved through the heat treatment of fullerene materials. The interaction between electron spins and oxygen molecules in carbon catalysts significantly affects the performance of oxygen reduction reactions. A promising approach to developing high‐performance catalysts involves introducing five‐membered ring structures with spin into graphitic carbon. Chen et al. synthesized caged carbon catalysts rich in pentagon structures and investigated their catalytic activity in high‐performance oxygen reduction reactions (Figure 39).^[^ ^128^ ^]^ First, this approach synthesized caged cubic carbon catalysts rich in pentagon structures using C_60_ as a carbon source and NaCl as a template. The number of pentagon structures in the resulting material was increased through nitrogen doping and annealing treatment. High‐resolution microscopic observation revealed that the bonded pentagon structures underwent changes during pyrolysis due to nitrogen doping, resulting in pentagon defects. Especially, nitrogen doping is important for generating high‐density active pentagon sites. This process also increases the number of electron spins, thereby improving the catalytic activity. The obtained carbon catalyst exhibited exceptional activity in the oxygen reduction reaction in acidic electrolytes. Correlating the pentagon ring structure with the number of spins and catalytic activity revealed that the oxygen reduction reaction activity of the pentagon‐containing carbon catalyst originates from the spin localized in the pentagon ring. DFT results for the O_2_ adsorption process of the model molecule showed that the singly occupied molecular orbital (SOMO) of the pentagon‐containing molecule is characterized by a π orbital, which is energetically favorable and promotes efficient oxygen adsorption. These results suggest that the pentagon structure plays an important role in O_2_ adsorption and that the spin is localized at the carbon atom in the pentagon, which is expected to be involved in O_2_ adsorption as the initial step in the oxygen reduction reaction. This approach enables control of the carbon skeleton at the unit level through material‐level nanoarchitectonics. Consequently, sensitive physical properties such as the spin state, O_2_ adsorption and catalytic activity can be freely controlled.

In this section, we have demonstrated how structures can be formed and organized in a flexible manner, starting from a common unit known as a fullerene. By adjusting the reaction and kinetic factors of the assembly process, diverse carbon structures can be formed. Additionally, nitrogen doping and thermal processes can be used to control the refinement of the carbon skeleton (pentagon structure) in carbon materials made from fullerene. These examples demonstrate that, even when using the same starting material, the potential for nanoarchitectonics in structure formation can be significantly increased through careful consideration of factors such as setting conditions and adjusting processes.

## 7 Summary and Future Perspective

Entitled “From Inception to Innovation: 20 Years of Nanoarchitectonics”, this review provides an overview of the development of functional material systems using nanoarchitectonics and related methodologies. The term “nanoarchitectonics” was coined as the 21st century approached. Therefore, it is fair to say that it has a history of almost 20 years. The main issue at stake is whether innovation is being brought about. However, nanoarchitectonics is a comprehensive concept and it is almost impossible to cover it all systematically. Rather than chronicling the past 20 years of nanoarchitectonics in full, we have selected several representative topics and examined their trends.

In atomic switches, the movement of atoms and the formation of clusters are converted into device technologies. On‐surface synthesis involves observing and controlling organic synthesis using nanotechnology—a fusion of nanotechnology and organic chemistry—and reflects the concept of nanoarchitectonics. Even in these typical nanoarchitectonics approaches, the latest research is focused on artificial intelligence. In other words, innovation with artificial intelligence is also coming to fundamental nanoarchitectonics. When organizing materials, limiting geometry can sometimes make the approach easier. For example, limiting the direction of organization to a layered structure makes nanoarchitectonics research more focused, and many developments have been made in this area. Approaches related to 2D materials and LbL assembly are characterized by their potential for various practical applications. This gives nanoarchitectonics a sense of innovation with an eye on practical use. One trend is the deployment of nanoarchitectonics in flexible media, such as liquid interfaces. Nanoarchitectonics at liquid interfaces demonstrates broad applicability to a range of systems, from simple molecules to complex biological systems. These range from single‐element zero‐dimensional molecules, such as fullerenes, to polymers, nanosheets, proteins, and even living cells. Furthermore, it has been demonstrated that vortex‐like movement of the liquid surface can precisely control molecular structure and orientation. The ability to control molecular structure through macroscopic movements that can be used for mass production paves the way for nanoarchitectonics to be applied in industry. Even when using the same starting material, taking a detailed approach to setting conditions and adjusting the process shows that the potential of nanoarchitectonics for structural formation can be significantly increased. Recently, examples have been reported in which the carbon framework in a material has been controlled. The number of papers claiming to be nanoarchitectonics has increased significantly in recent years, as has the scope of its application. It could be said that nanoarchitectonics is a method for everything in materials science.

As can be seen from the above summary, the seeds of innovation in nanoarchitectonics are beginning to emerge. Based on these trends, let us briefly consider the areas that will be emphasized particularly in the future. One is the application of artificial intelligence. Several examples of the introduction of artificial intelligence have already emerged, and its importance will increase. Nanoarchitectonics research has expanded in recent years and is now being applied to a wide range of materials and functions. Beyond that, nanoarchitectonics with a huge variety of components together have to be considered with the aid of artificial intelligence. As models of future targets of nanoarchitectonics, biological tissues are composed of many functional molecules that are rationally assembled and perform advanced functions through their cooperation. We should aim to achieve something similar with our own materials. Artificial intelligence is needed to design such complex systems, select organizational methods and predict functions. Many research examples have demonstrated the compatibility of methods such as machine learning with chemistry and materials science.^[^ ^129^ ^]^ There is also a need to combine the fields of materials informatics and nanoarchitectonics.^[^ ^130^ ^]^ Further development of nanoarchitectonics, which is spreading to many material systems, requires artificial intelligence capable of handling complex systems. Integration of artificial intelligence and nanoarchitectonics has actively started. Inclusion of materials informatics strategies for nanoarchitectonics of functional nanoporous materials has been proposed.^[^ ^130a^ ^]^ Approaches and roadmaps with machine learning for nanoarchitectonics for functional nanoparticles have been indicated with condition optimization and automatic syntheses.^[^ ^130b^ ^]^ Very recently, artificial intelligence‐driven nanoarchitectonics for smart targeted drug delivery has been propose.^[^ ^130c^ ^]^ As seen in these recent examples, the introduction of artificial intelligence into nanoarchitectonics approaches has huge future potential in a wide range of target materials and functions.

Some nanoarchitectonics research approaches are reaching a highly practical stage, as demonstrated by the development of layered structures. Additionally, the example of liquid interface nanoarchitectonics using flow demonstrates that nanostructures, such as chiral structures, can be controlled by common macroscopic actions, such as stirring liquids. This further supports the idea that practical actions are being linked to nanoscience. In this context, the aim is to perform nanoarchitectonics using operations employed in many production processes. This involves the mass production or large‐area creation of nano‐level controlled structures. This is an unavoidable path for academic knowledge of nanoarchitectonics to be put to practical use. This is expected to pave the way for the industrialization of nanoarchitectonics.

## Conflict of Interests

The authors declare no conflict of interest.

## Data Availability

Data sharing is not applicable to this article as no new data were created or analyzed in this study.
